# Azetidine amino acid biosynthesis by non-haem iron-dependent enzymes

**DOI:** 10.1038/s41557-025-01958-x

**Published:** 2025-10-21

**Authors:** Yanan Du, Anyarat Thanapipatsiri, Jesús J. Blancas Cortez, Xavier E. Salas-Solá, Chi-Yun Lin, Amie K. Boal, Carsten Krebs, J. Martin Bollinger, Kenichi Yokoyama

**Affiliations:** 1 Department of Biochemistry, Duke University School of Medicine, Durham, NC USA; 2 Department of Chemistry, The Pennsylvania State University, University Park, PA USA; 3 Department of Biochemistry and Molecular Biology, The Pennsylvania State University, University Park, PA USA; 4 Department of Chemistry, Duke University, Durham, NC USA

**Keywords:** Enzyme mechanisms, Biosynthesis, Biocatalysis

## Abstract

Azetidine, a four-membered aza-cycle, is a crucial structure in many bioactive compounds and drugs. However, their biosynthesis is frequently enigmatic. Here we report the mechanism of azetidine amino acid (polyoximic acid) biosynthesis in the polyoxin antifungal pathway. Genetic, enzymological and structural experiments revealed that PolF is a member of haem-oxygenase-like dimetal oxidase and/or oxygenase (HDO) superfamily, and this enzyme alone is sufficient for the transformation of l-isoleucine (l-Ile) and l-valine to their azetidine derivatives via a 3,4-desaturated intermediate. Mechanistic studies of PolF suggested that a μ-peroxo-Fe(III)_2_ intermediate is directly responsible for the unactivated C–H bond cleavage, and the post-H-abstraction reactions, including the C–N bond formation, probably proceed through radical mechanisms. We also found that PolE, a member of the DUF6421 family, is an Fe and pterin-dependent oxidase that catalyses the desaturation of l-Ile, assisting PolF by increasing the flux of l-Ile desaturation. The results provide important insights into azetidine biosynthesis and the catalytic mechanisms of HDO enzymes in general.

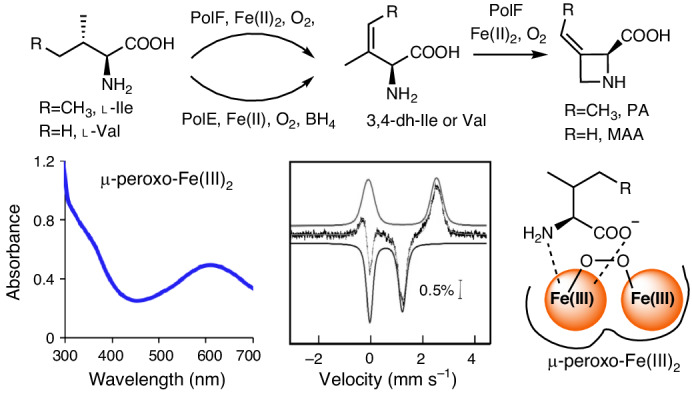

## Abstract (web summary)

Azetidine is a four-membered aza-cycle important in medicinal and organic chemistry. This study describes a mechanism of azetidine amino acid biosynthesis from l-isoleucine or l-valine by two non-haem Fe enzymes, PolF and PolE, in the polyoxin antifungal biosynthetic pathway.

## Main

**Extended Data Fig. 1:**
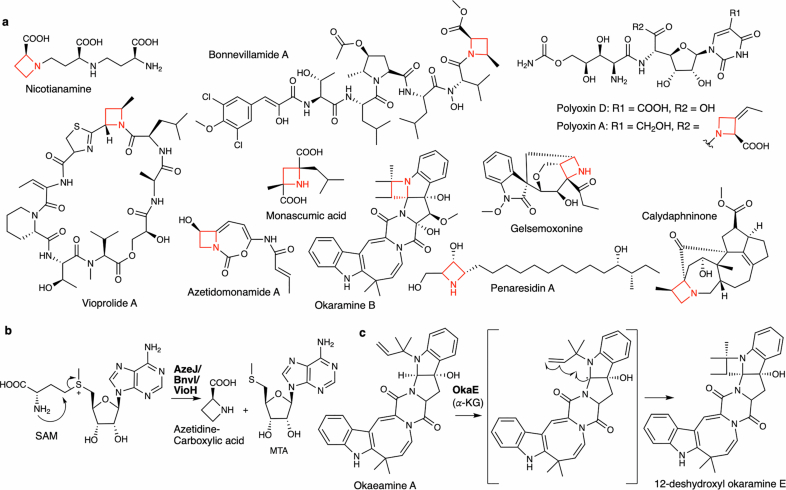
Azetidine ring containing natural products and known mechanisms of azetidine ring formation. (**a**) Azetidine ring containing natural products. (**b**) and (**c**) Known mechanisms of azetidine ring formation in the biosynthesis of natural products.

Azetidine is a four-membered nitrogen-containing saturated heterocycle. Due to its high ring strain (25.4 kcal mol^−1^)^1^, azetidine is a useful substrate for ring opening or expansion, metal-catalysed transformations and asymmetric reactions^2^. Azetidine is also found in many compounds with important bioactivity^3^ (Extended Data Fig. 1a). However, our understanding of the mechanism of biosynthesis of azetidine in natural products remains limited^4^. The best-characterized mechanism involves *S*-adenosyl-l-methionine (SAM) dependent enzymes, which catalyse an intramolecular nucleophilic cyclization of SAM to yield azetidine carboxylic acid and 5′-methylthioadenosine^5,6^ (Extended Data Fig. 1b). Another known mechanism occurs in the biosynthesis of okaramine, where an α-ketoglutarate (α-KG) and Fe-dependent oxygenase catalyses a radical-mediated oxidative C–C bond formation to produce the azetidine ring^7^ (Extended Data Fig. 1c). These precedents, however, require metabolically or chemically expensive precursors, limiting their biocatalytic applications.

Antifungal nucleoside polyoxin A contains an azetidine amino acid called polyoximic acid (PA)^8^. Early isotope-labelling experiments suggested that PA is derived from l-isoleucine (l-Ile), indicating a distinct biosynthetic mechanism from those previously reported^9^ (Extended Data Fig. 1b,c). In the polyoxin biosynthetic gene cluster, putative biosynthetic enzymes (PolE and PolF) have been implicated in PA biosynthesis^10^. PolF and PolE are proteins of unknown function in the DUF6202 and DUF6421 families, respectively. While another enzyme, PolC, a putative α-KG and Fe-dependent oxygenase, was also previously proposed to be involved in the PA biosynthesis^10^, recent functional elucidation of PolD and PolK, α-KG or Fe-dependent enzymes responsible for the biosynthesis of the nucleoside moiety, and the similarity of PolC to PolD and PolK, suggested that PolC is unlikely to be involved in the PA biosynthesis^11,12^. Although several mechanisms for PA formation have been proposed^13^, no experimental data have been provided to support these claims.

Here, we report the elucidation of the mechanism of PA biosynthesis. On the basis of the gene knockout experiments, in vitro functional characterization, crystal structures and spectroscopic characterization of the Fe_2_ cluster, we demonstrate that PolF is a member of the haem-oxygenase-like dimetal oxidases and/or oxygenase (HDO) family and catalyses the transformation of l-Ile and l-valine (l-Val) to their azetidine derivatives via a desaturated intermediate. The results also revealed that PolF uses a μ-peroxo-Fe(III)_2_ species to catalyse chemically challenging C–H activation (up to 101 kcal mol^−1^). Together with the crystal structure of PolF in complex with l-Ile, we propose the mechanism of C–N bond formation. We also found that PolE is an Fe-dependent and pterin-dependent oxidase that assists PolF by catalysing a specific desaturation of l-Ile, making the biosynthetic pathway both specific and efficient. The combined results elucidate the mechanism of enzymatic azetidine formation and substantially extend our understanding of the catalytic functions and mechanisms of two classes of non-haem iron enzymes.

## Results

### PolF is essential for PA biosynthesis

**Fig. 1:**
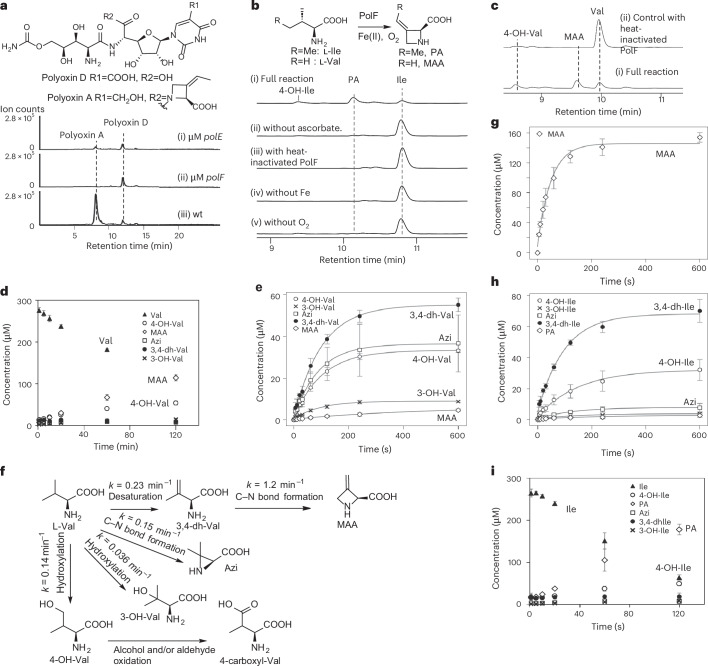
Characterization of the PA biosynthesis. **a**, LC–MS analysis of the fermentation broths of *S. cacaoi* and its mutants. Shown are the EICs for polyoxin A (EIC = 617.2053) and the EIC for polyoxin D (EIC = 522.1317). **b**, Transformation of l-Ile to PA by PolF. HPLC UV chromatograms (325 nm) of PolF assays containing 300 μM l-Ile, 30 μM apo-PolF, 100 μM Fe(II), 1 mM ascorbate and ~0.5 mM O_2_ (i). Also shown are control assays performed under the same conditions but without ascorbate (ii), with boiled PolF (iii), without Fe (iv) and without O_2_ (v). **c**, Transformation of l-Val to MAA by PolF. HPLC UV chromatograms (325 nm) of a PolF assay with 300 μM l-Val, 30 μM apo-PolF, 100 μM Fe(II), 1 mM ascorbate and ~0.5 mM O_2_ (i), and a control assay with boiled PolF (ii). See Supplementary Fig. 4 for the HPLC chromatograms of the entire retention time range for **b** and **c**. **d**,**e**,**g**, PolF reactions with l-Val (3,4-dh-Val) under multiple turnover (**d**) and single turnover conditions (**e**), and with 3,4-dh-Val under single turnover conditions (**g**). **f**, Reactions catalysed by PolF with l-Val as substrate. The rate constant of each step is calculated from the kinetic fittings of the data in **e** and **g**. See Supplementary Table 3 for details. **h**,**i**, PolF reactions with l-Ile under single turnover (**h**) and multiple turnover conditions (**i**). The products of the l-Ile products were characterized by LC–MS and a comparison with the l-Val reaction. The solid lines in **e**,**g**,**h** are a nonlinear curve fit of equation (1) (Methods). All the kinetic parameters are summarized in Supplementary Table 3. The kinetic experiments were performed in triplicate. Error bars represent one standard deviation calculated from the three replicates. Source data for **a–e**, **g–i** are provided. Source data

We first performed disruption of *polE* and *polF* genes in the polyoxin producer *Streptomyces cacaoi*. The two genes were individually deleted in-frame (Supplementary Fig. 1), and the mutants were cultured under polyoxin-producing conditions. The fermentation broth of each culture was analysed by liquid chromatography–mass spectrometry (LC–MS) (Fig. 1a). While the *polE* mutant produced a reduced but detectable amount of polyoxin A (~10% of wt), the *polF* mutant did not produce any measurable amount of polyoxin A (<1%). These results indicated that PolF is essential for PA biosynthesis, while PolE may increase the titre.

### PolF catalyses the transformation of l-Ile to PA

The amino acid sequence of PolF shows no homology to functionally characterized enzymes. However, when analysed for structural homology by Foldseek^14^, PolF resembles several characterized enzymes in the HDO superfamily, an emerging group of diiron-dependent enzymes that activate O_2_ to catalyse various oxidative reactions. Known HDO-catalysed transformations include oxidative C–C bond cleavage to form terminal alkene^15^, nitrile or methyl group^16–19^, N-oxidation^20–23^, methylene excision^21^ and desaturation^24^ (Supplementary Fig. 2). However, no HDOs characterized to date can construct the azetidine ring found in PA. Thus, we proposed that PolF is an HDO enzyme with a previously unseen catalytic function.

To investigate the catalytic function, we expressed and purified PolF from *E. coli* (Supplementary Fig. 3). PolF was initially isolated in a largely apo form. Since HDOs require 2 eq. of Fe(II), apo-PolF was incubated with excess (3 eq.) Fe(II) under anaerobic conditions. After removal of the unbound Fe(II) using a desalting column, the resulting PolF contained 1.5 eq. Fe(II). The less than 2 eq. Fe bound to PolF is consistent with the reported weak affinity of Fe to HDO enzymes in the absence of substrate^25^.

We then performed activity assays using l-Ile and apo-PolF reconstituted with excess Fe(II). The enzyme and substrate mixture was prepared anaerobically, and the reactions were initiated by the addition of an O_2_-saturated buffer. We subsequently derivatized the reaction products with dansyl chloride (DnsCl), and analysed by LC–MS. Consequently, we observed a product with a molecular weight of −4 Da compared with l-Ile (Fig. 1b(i) and Supplementary Fig. 4a). This product was identified as PA by a comparison with a PA authentic standard prepared from polyoxin A (Supplementary Fig. 5) and structural characterization by NMR^26^ (Supplementary Fig. 6). PA was not observed in reactions without Fe(II) or O_2_ or with heat-inactivated PolF (Fig. 1b(ii)–(v)). The dependence of PolF activity on ascorbate or dithiothreitol (Supplementary Fig. 7) is consistent with the need for an external reductant for the re-reduction of the diiron centre during the multiple turnovers of HDO enzymes. We also tested other transition metals and found that only the assays with Fe(II) produced PA (Supplementary Fig. 8). Thus, these results demonstrate that PolF is a non-haem Fe enzyme and catalyses the transformation of l-Ile to PA.

### Substrate specificity of PolF

The substrate specificity of PolF was studied using the 20 proteogenic amino acids. Most importantly, we found that the reaction with l-Val yielded a product with a −4-Da modification (Fig. 1c and Supplementary Fig. 4b). This product was isolated and structurally characterized by NMR as 3-methyene-azetidine-2-carboxylic acid (MAA) (Supplementary Fig. 9). Of the other 18 amino acids, l-leucine (l-Leu) and l-methionine (l-Met) yielded mostly hydroxylation products with minor desaturation products (Extended Data Table 1 and Supplementary Fig. 10). No other amino acids yielded detectable products, suggesting that PolF reacts selectively with medium-size aliphatic amino acids. The absence of azetidine products from l-Met or l-Leu suggests that the β-methyl group is critical for the azetidine formation. We also investigated l-Ile stereoisomers. l-*allo-*Ile, d-Ile and d-*allo-*Ile all yielded small but detectable amounts of azetidine products (Extended Data Table 1 and Supplementary Fig. 11), suggesting that the stereochemistries at C2 and C3 are important but not essential for the azetidine formation.

### How PolF forms azetidine

**Extended Data Fig. 2:**
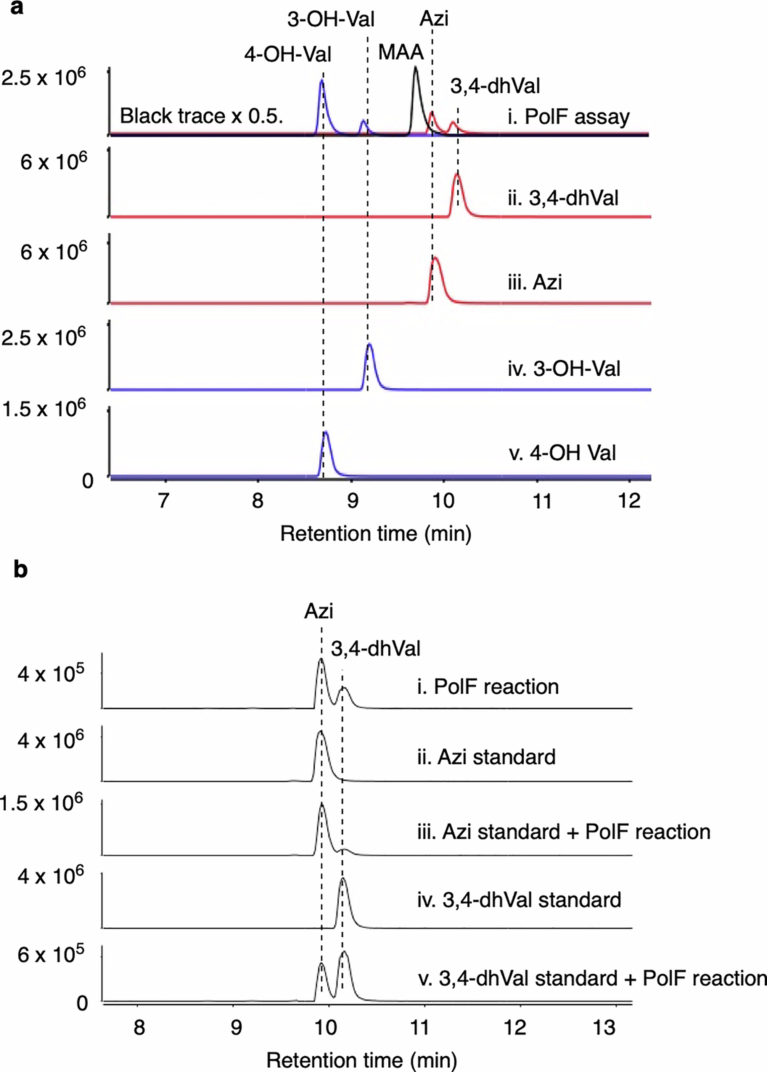
LC-MS analysis of PolF reaction with l-Val and its comparison with standards. (**a**) EIC of the PolF assay at 120 min (i), and authentic standards: 3,4-dhVal (ii), Azi (iii), 3-OH-Val (iv) and 4-OH-Val (v). EIC = 349.1217 (red), EIC = 367.1323 (blue) and EIC = 347.1060 (black). (**b**) EIC = 349.1217, reaction of l-Val with PolF (i), Azi standard (ii), co-injection of Azi standard and the PolF reaction (iii), 3,4-dhVal standard (iv), and co-injection of 3,4-dhVal standard and the PolF reaction (v). Source data are provided. Source data

To gain mechanistic insights, we looked for reaction intermediates. To this end, we first characterized the PolF reaction with l-Val because various analogues were commercially available. Under multiple turnover conditions, the PolF reaction with l-Val yielded MAA as the major product (Fig. 1d and Supplementary Fig. 12). However, careful LC–MS analysis revealed four additional l-Val derivatives with −2-Da and +16-Da modifications (Fig. 1d and Supplementary Fig. 12), which were identified as 3-hydroxyvaline (3-OH-Val), 4-hydroxyvaline (4-OH-Val), 3,4-dehydrovaline (3,4-dh-Val) and 3-dimethylaziridine-2-carboxylic acid (Azi) by comparing with authentic standards (Extended Data Fig. 2). The formation of Azi was unexpected and consistent with the ability of PolF to catalyse the C–N bond formation. To obtain further insights, we performed PolF assays under single turnover conditions, in which 2 eq. Fe(II) and no reductant were used (Fig. 1e). As a consequence, we found 3,4-dh-Val as the major product (0.23 min^−1^), followed by Azi, 4-OH-Val and 3-OH-Val (0.15 min^−1^, 0.14 min^−1^ and 0.036 min^−1^). These observations indicate that PolF catalyses three distinct reactions, desaturation, hydroxylation and C–N bond formation, on a single substrate (Fig. 1f).

To identify the intermediate of azetidine formation, we performed PolF assays using l-Val as substrate. As a consequence, we found that 3,4-dh-Val was quantitatively converted into MAA (Fig. 1g and Supplementary Fig. 13). The rate of transformation of 3,4-dh-Val into MAA (1.2 ± 0.4 min^−1^) was faster than the transformation of l-Val to 3,4-dh-Val (0.23 ± 0.04 min^−1^), suggesting that 3,4-dh-Val formation is the rate-determining step and explains the minimal accumulation of 3,4-dh-Val under multiple turnover conditions (Fig. 1d). 4-OH-Val was converted to 4-carboxy-Val (Fig. 1f and Supplementary Fig. 14). Azi was consumed very slowly without the formation of a detectable product, suggesting that Azi is degraded through ring-opening radical fragmentation or reacted with the enzyme or surrounding molecules (Supplementary Fig. 15). 3-OH-Val was not reactive at all (Supplementary Fig. 16). Overall, these observations demonstrate that PolF catalyses the azetidine MAA formation via 3,4-dh-Val as an intermediate.

Subsequently, we characterized the PolF reaction with l-Ile. Similar to the l-Val reaction, under single turnover conditions, the l-Ile reaction yielded four products with dh-Ile as the major product (Fig. 1h). Under multiple turnover conditions, PolF produced PA as the major product (Fig. 1i and Supplementary Fig. 17). The comparison of the PolF reactions with l-Val and l-Ile revealed that PolF is more specific and efficient with l-Ile as substrate than the reaction with l-Val on the basis of the faster rate of desaturation (0.23 min^−1^ versus 0.28 min^−1^; Fig. 1e versus Fig. 1h), the higher yield of the desaturation product (48% versus 70%; Fig. 1d versus Fig. 1h), and the faster rate of azetidine formation under multiple turnover conditions (0.030 min^−1^ versus 0.046 min^−1^; Fig. 1e versus Fig. 1i). These observations are consistent with l-Ile as the physiological substrate of PolF. Still, the overall product profile is very similar between l-Ile and l-Val, and the desaturated derivative served as the intermediate of azetidine formation. Therefore, for both l-Val and l-Ile substrates, PolF catalyses the azetidine formation via a conserved two-step mechanism through the 3,4-dehydro intermediate.

### PolF catalysis proceeds via μ-peroxo-Fe(III)_2_ intermediate

**Fig. 2:**
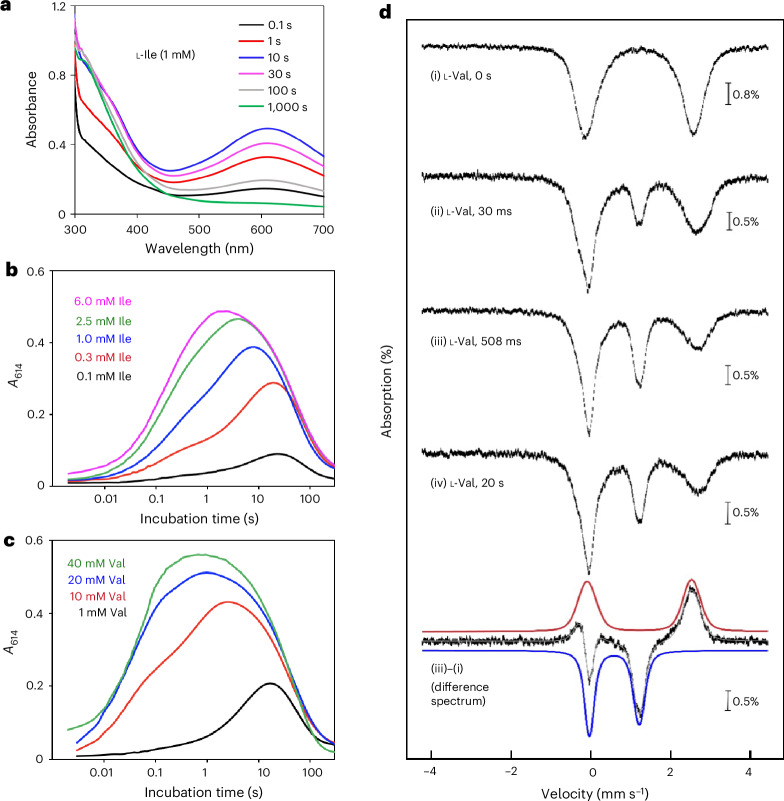
Substrate-triggered O_2_ activation by PolF. **a**, Absorption spectra acquired after a rapid mixing at 5 °C of an anoxic solution of 0.3 mM PolF, 1 mM l-Ile and 0.6 mM Fe(II) with an equal volume of O_2_-saturated buffer. **b**,**c**, Stopped-flow traces monitored at 614 nm. The reaction conditions were identical to **a** except that the substrate (l-Ile (**b**) or l-Val (**c**)) concentration was varied as shown in the figures. Each trace was fit to equation (2) to determine the rate constants summarized in Supplementary Table 4. **d**, Mössbauer spectra of the PolF reaction using l-Val as a substrate. Spectra were acquired at 4.2 K in a 53-mT magnetic field externally applied parallel to the propagation direction of the γ beam. The experimental spectra are depicted by vertical bars of heights reflecting the standard deviations of the absorption values during the acquisition of the spectra. Spectrum (i) is an anoxic solution of the reactant complexes (1.44 mM PolF, 2.88 mM ^57^Fe(II) and 40 mM l-Val). Spectra (ii)–(iv) are the reactions initiated by mixing the anoxic solution with O_2_-saturated buffer and incubated at 5 °C for the time shown in the figure. Shown at the bottom is the difference spectra (iii–i) overlaid with their simulations with quadrupole doublets, demonstrating the formation of μ-peroxo-Fe(III)_2_ (blue trace *δ* = 0.58 mm s^−1^ and Δ*E*_Q_ = 1.26 mm s^−1^) at the expense of the consumption of high-spin Fe(II)_2_ (red trace *δ* = 1.23 mm s^−1^ and Δ*E*_Q_ = 2.65 mm s^−1^). Source data for **a**, **b**, **c** are provided. Source data

**Fig. 3:**
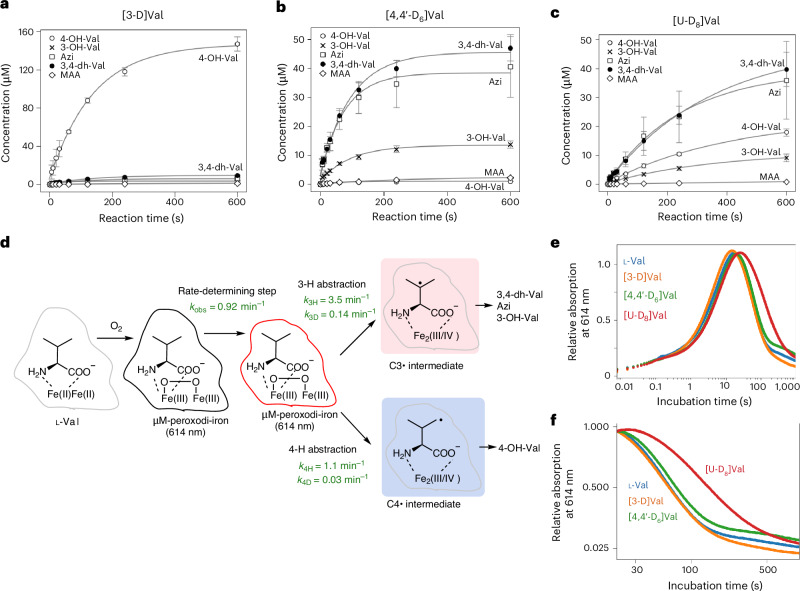
Mechanistic investigation of PolF catalysis using deuterated l-Val under single turnover conditions. **a**–**c**, Shown are the time course analysis of product formation with [3-D]Val (**a**), [4,4′-D_6_]Val (**b**) and [U-D_8_]Val (**c**). The solid lines are the nonlinear curve fit of the equation $P(t)={{A}_{0}-A{\rm{e}}}^{-kt}$ using the parameters in Supplementary Table 3. The identities of the deuterated products were confirmed by LC–MS (Supplementary Fig. 18). **d**, Mechanisms of PolF-catalysed oxidative transformations of l-Val. **e**, Stopped-flow analysis of the PolF reaction with deuterated l-Val. Shown are the stopped-flow traces at *A*_614nm_ to monitor the formation and decay of the μ-peroxo-Fe(III)_2_ intermediate. **f**, The decay of *A*_614nm_ analysed by stopped-flow analysis in **e**. The intensities of the stopped-flow traces in **e** and **f** are normalized by the maximum intensity of each trace (at ~10 s) and the relative absorbance at 614 nm is reported. The traces without normalization are shown in Supplementary Fig. 19. Each trace was fit to equation (2) to determine the rate constants summarized in Supplementary Table 4. The kinetic experiments were performed in triplicate. Error bars represent one standard deviation calculated from the three replicates. Source data for **a**, **b**, **c**, **e**, **f** are provided. Source data

**Fig. 4:**
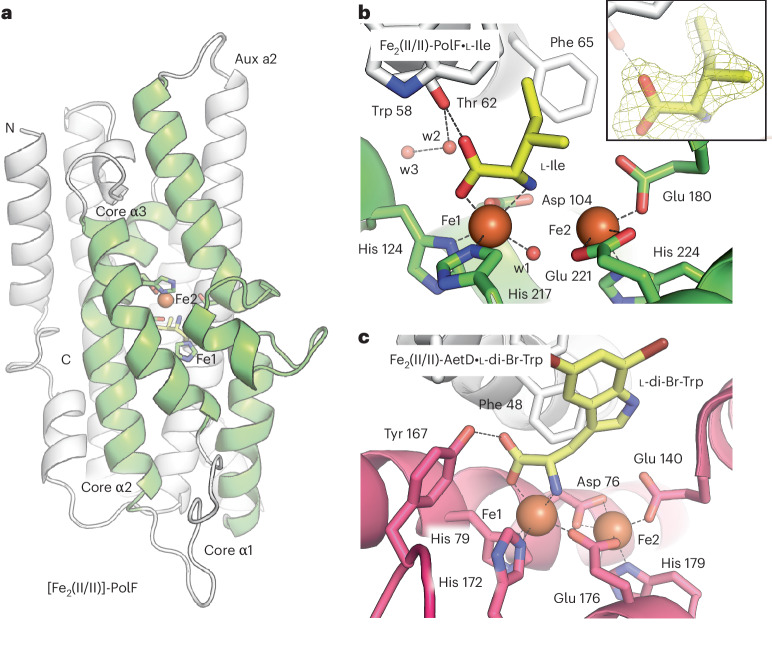
X-ray crystal structures of PolF. **a**, Overall structure of Fe_2_(II/II)•l-Ile•PolF. **b**, Zoomed-in view of the metal and substrate-binding sites in the active site of a representative chain (chain F). Inset shows a 2*F*_o_*–F*_c_ map (yellow mesh) for l-Ile contoured at 1.0σ. **c**, Comparative view of the substrate-binding site in AetD, an HDO that engages its amino acid substrate similarly to PolF (PDB accession code 8TWW). Selected amino acid side chains are shown as sticks, water molecules are shown as red spheres, iron ions are shown as orange spheres and coordination bonds and hydrogen bonds are shown as dashed lines. Source data

**Extended Data Fig. 3:**
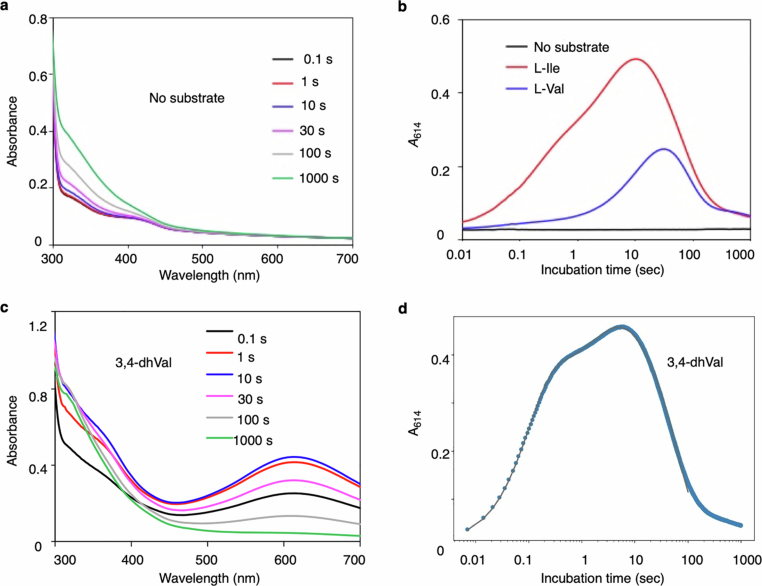
Stopped-flow analysis of O_2_ activation by PolF. (**a**) Absorption spectra acquired after a rapid mixing at 5 °C of an anoxic solution of 0.3 mM PolF and 0.6 mM Fe(II) with an equal volume of O_2_-saturated buffer. (**b**) Absorption kinetic traces monitored at 614 nm. An anoxic solution of PolF (0.3 mM), Fe(II) (0.6 mM, 2 molar equivalent), and substrate (1 mM l-Ile or 1 mM l-Val or no substrate) was mixed with an equal volume of O_2_-saturated buffer. (**c** and **d**) Stopped-flow analysis of O_2_ activation by PolF with 3,4-dhVal. Absorption spectra (**c**) and absorption kinetic traces monitored at 614 nm (**d**) acquired after a rapid mixing at 5 °C of an anoxic solution of 0.3 mM PolF and 0.6 mM Fe(II), 1 mM 3,4-dhVal with an equal volume of O_2_-saturated buffer. Source data are provided. Source data

To obtain further evidence for PolF as an HDO, we characterized the putative Fe_2_ cluster. In some mechanistically characterized HDO enzymes, the Fe binding and O_2_ activation are coupled to substrate binding^18,25,27^. Therefore, to investigate the substrate-triggered cluster assembly, we performed stopped-flow experiments. Mixing an anaerobic solution containing apo-PolF, l-Ile and 2 eq. Fe(II) with O_2_-saturated buffer resulted in the rapid development of an intense, transient visible absorption feature centred at ~614 nm (Fig. 2a). The formation of the 614-nm feature was l-Ile concentration-dependent (Fig. 2b) and was not observed in the absence of substrate (Extended Data Fig. 3a,b), suggesting the substrate-triggered O_2_ activation, similar to those reported for other HDO enzymes^18,25,27^. Formation of a similar absorption feature was observed with l-Val (Figs. 2c) and 3,4-dh-Val (Extended Data Fig. 3c,d). l-Val required a higher concentration to saturate the kinetics of the 614 nm feature formation (Fig. 2c). The observation of the same absorption feature with 3,4-dh-Val suggests that the azetidine ring formation proceeds through the same intermediate. The observed absorption feature is reminiscent of those associated with μ-peroxo-Fe(III)_2_ complexes in UndA (~550 nm), SznF (~629 nm), AetD (~625 nm) and BesC (~618 nm)^18,25,27^. This assignment is corroborated by freeze-quench Mössbauer spectroscopy, which reveals the accumulation of a quadrupole doublet with parameters typical of μ-peroxo-Fe(III)_2_ clusters (isomer shift *δ* = 0.58 mm s^−1^ and quadrupole splitting parameter Δ*E*_Q_ = 1.26 mm s^−1^, Fig. 2d)^28^. These observations are consistent with our proposal that PolF deploys a diiron cofactor and accumulates a μ-peroxo-Fe(III)_2_ complex on the addition of O_2_, as in other HDOs. Since PolF requires substrates (l-Ile, l-Val or 3,4-dh-Val) to trigger O_2_ activation, the enzyme belongs to a subset of HDOs that are substrate-triggered.

### PolF cleaves unactivated C–H bonds

While nearly all HDOs characterized to date accumulate a μ-peroxo-Fe(III)_2_ intermediate on reaction with O_2_, it remains ambiguous whether this intermediate is directly responsible for reaction with substrate. The involvement of a μ-peroxo-Fe(III)_2_ complex in HDOs that initiate reactions via H-atom abstraction is surprising because more potent high-valent Fe intermediates, such as Fe(IV)_2_ in methane monooxygenase, are generally implicated for this chemistry in other diiron enzymes^29^. Among HDOs, PolF targets an extremely challenging substrate, especially l-Val, because the transformation probably involves the abstraction of an unactivated C–H bond (up to 101 kcal mol^−1^)^30–32^. The characterization of l-Val reaction is also advantageous to the l-Ile reaction because l-Val isotopologues are accessible. Therefore, we characterized the H-atom abstraction step in the PolF catalysis using l-Val as substrate.

The rate of H abstraction is sensitive to substrate deuteration. Thus, we first investigated the effects of deuterated substrates on the PolF reaction kinetics and product profile using [3-D]Val, [4,4′-D_6_]Val and [U-D_8_]Val. When [3-D]Val was used, 4-OH-Val became the major product. All other products, including 3,4-dh-Val, the dominant product from l-Val, produced less than 10% of the product pool (Fig. 3a). When [4,4′-D_6_]Val was used, 3,4-dh-Val and Azi were produced as the major product, and 4-OH-Val formation was negligible (Fig. 3b). Finally, when [U-D_8_]Val was used, the product profile was similar to that of unlabelled l-Val, but the product formation rates were slowed by a factor of three (Fig. 3c). These observations suggest that 3-dh-Val, 3-OH-Val and Azi are formed via 3-H abstraction and 4-OH-Val is formed via 4-H abstraction. This analysis also shows that when 3-H or 4-H is replaced with D, PolF preferentially abstracts H from the unlabelled position to avoid the more challenging D abstraction, resulting in significant shifts in the product profile. These results provide experimental evidence that PolF initiates its reaction via cleavage of unactivated C–H bonds. Our findings also suggest that 3-H and 4-H are both readily accessible to the reactive intermediate(s), and the reaction outcome is determined by the relative rates of the C–H bond cleavage reaction between the two sites (Fig. 3d).

### KIE reveals tunnelling effects and the rate-determining step

Because the ratio of the products in the single turnover assays is determined by the relative rates of the H-abstraction step (Fig. 3d), the results from the l-Val isotopologue reactions described above can be used to determine the intrinsic 1° deuterium kinetic isotope effect (D-KIE) for the 3-H and 4-H abstraction steps by intramolecular competition. As detailed in the Supplementary Note 1, on the basis of the difference in the product ratio of the isotopologue reactions, we determined the D-KIE for 3-H and 4-H abstraction as 25 and 36, respectively. These values exceed the semiclassical limit of 1° D-KIE of ~7 and are comparable to those observed in other non-haem Fe enzymes that catalyse C–H activation reactions with a quantum-mechanical tunnelling effect^33,34^.

**Fig. 5:**
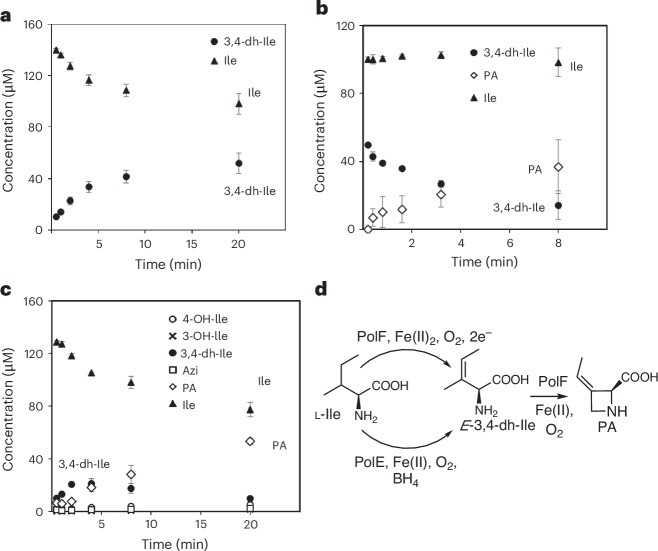
Functional characterization of PolE. **a**, Quantitative analysis of PolE time course assays with l-Ile. The reactions were quenched at 30 s, 1 min, 2 min, 4 min, 8 min and 20 min. **b**, Quantitative analyses of PolEF stepwise assay with l-Ile. PolE assay (500 μl) was performed with 150 μM l-Ile, 15 μM PolE, 100 μM Fe(II), 100 μM BH_4_, 1 mM ascorbate, 1 mM NADH, 0.5 μM QDPR and ~0.5 mM O_2_ at room temperature for 40 min. Then 15 μM PolF was added to reaction. The mixtures were incubated for 30 s, 1 min, 2 min, 4 min and 8 min. **c**, Quantitative analysis of PolEF coupled assays with l-Ile. The assay contains 150 μM l-Ile, 15 μM PolE, 15 μM PolF, 100 μM Fe(II), 100 μM BH_4_, 1 mM ascorbate, 1 mM NADH, 0.5 μM QDPR and ~0.5 mM O_2_. The reactions were quenched at 30 s, 1 min, 2 min, 4 min, 8 min and 20 min. **d**, PA biosynthesis by PolE and PolF. The experiments were performed in triplicate. Error bars represent one standard deviation calculated from the three replicates. Source data for **a, b, c** are provided. Source data

To obtain further kinetic insights, we performed stopped-flow experiments using l-Val isotopologues (Fig. 3e,f). Since the μ-peroxo-Fe(III)_2_ intermediate should be consumed on H-atom abstraction, the decay rate of the absorbance at 614 nm (*A*_614nm_) is potentially sensitive to D-KIE. When [3-D]Val and [4,4′-D_6_]Val were used, no significant KIE (1.0 and 1.2, respectively) was observed, consistent with the minimal D abstraction (Fig. 3e,f and Supplementary Table 4). On the other hand, when [U-D_8_]Val was used, a KIE of 3.1–3.6 was observed (Figs. 3e and 5f and Supplementary Table 4), which was comparable to the KIE of 3.2 on the basis of the product formation (Fig. 3c and Supplementary Table 3). This apparent KIE is significantly smaller than the intrinsic KIE of H abstraction (25 and 36). While the KIE of μ-peroxo-Fe(III)_2_ decay could be underestimated if there is a significant uncoupling of μ-peroxo-Fe(III)_2_ decay and substrate oxidation^25,35^, we eliminated such a possibility on the basis of the calculated coupling efficiency^25^ (CE_H_/CE_D_) of ~1.1. Thus, the discrepancy between the apparent and intrinsic KIE suggests the presence of a rate-determining step between μ-peroxo-Fe(III)_2_ formation and the H abstraction (Fig. 3d).

On the basis of the observed KIEs, we determined the rate constants for the 3-H and 4-H abstraction steps to be *k*_3H_ = 3.5 min^−1^ and *k*_4H_ = 1.1 min^−1^ (Supplementary Note 2). In general, the rate of H-atom abstraction by HDOs is uncharacterized or masked by a large uncoupling between the μ-peroxo-Fe(III)_2_ decay and H abstraction^25^. The results shown here represent a comprehensive kinetic characterization of the H-abstraction step by an HDO enzyme.

### μ-peroxo-Fe(III)_2_ and H abstraction in PolF

**Extended Data Fig. 4:**
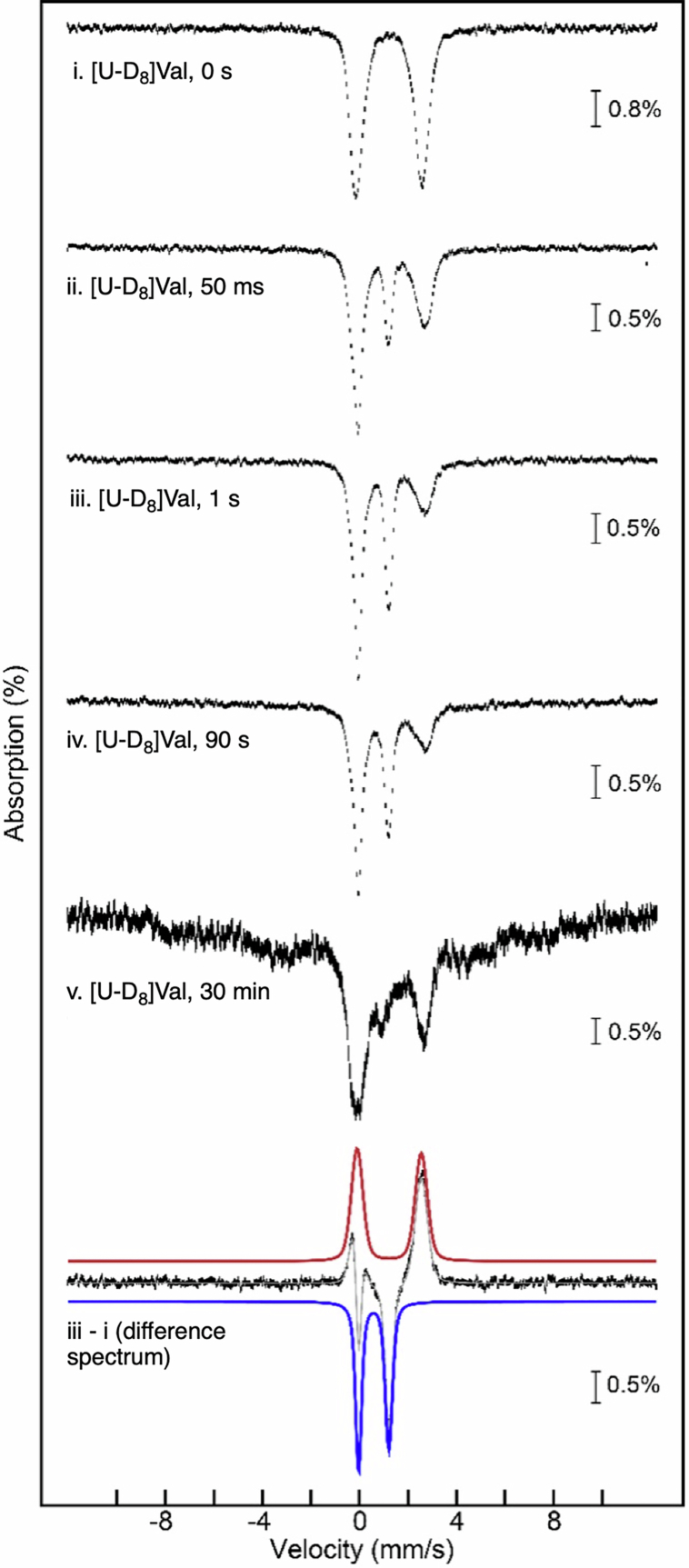
Mössbauer spectra of the PolF reaction using l-[U-D_8_]Val as a substrate. Spectra were acquired at 4.2 K in a 53-mT magnetic field externally applied parallel to the propagation direction of the γ beam. The experimental spectra are depicted by vertical bars of heights reflecting the standard deviations of the absorption values during the acquisition of the spectra. The top spectra (i) in each panel is that of an anoxic solution of the reactant complex (1.44 mM PolF, 2.88 mM ^57^Fe(II), and 40 mM [U-D_8_]Val). Spectra ii – v are the reactions initiated by mixing the anoxic solution with O_2_ saturated buffer and incubated at 5 °C for the time shown in the figure. Shown at the bottom is the difference spectra (iii - i) overlaid with their simulations with quadrupole doublets, demonstrating the formation of μ-peroxo-Fe(III)_2_ (blue trace: δ = 0.58 mm/s, and ΔE_Q_ = 1.26 mm/s) in expense of the consumption of high-spin Fe(II)_2_ (red trace: δ = 1.23 mm/s and ΔE_Q_ = 2.65 mm/s). Peak quantitation revealed 32, 51, and 49% of total Fe is associated with μ-peroxo-Fe(III)_2_. Spectrum v (30 min incubation) is dominated by the broad, magnetically split features associated with mononuclear high-spin Fe(III) ( ~ 80% of total intensity), in addition to the quadrupole doublet associated with the non-reactive Fe(II) complex(es) at δ = ~0 mm/s and ~3 mm/s (~20% of total intensity), suggesting the cluster destruction as observed in other HDO enzymes.

Characterization of H-abstracting species in HDO has been challenging due to the very small apparent KIE in AetD (1.4)^18^ or a large uncoupling of μ-peroxo-Fe(III)_2_ decay and substrate oxidation in BesC (CE_H_/CE_D_ = 2–12)^25,35^. Thus, the absence of uncoupling in PolF and the largest apparent substrate D-KIE reported for HDOs provide a unique opportunity to seek Fe_2_ species responsible for H abstraction. Our kinetic simulation using the rate constants determined above suggests that the PolF reaction with [U-D_8_]Val accumulates Fe_2_ intermediate responsible for the H abstraction ~50% of the total cluster at the 90-seconds time point (Supplementary Fig. 20). Thus, we characterized the PolF reaction with [U-D_8_]Val by stopped-flow (Supplementary Fig. 21) and Mössbauer spectroscopy (Extended Data Fig. 4). As a result, at 90 seconds, we observed the accumulation of μ-peroxo-Fe(III)_2_ to ~50% of total iron, and no evidence for the presence of any other intermediates. Since Fe(IV)_2_ and Fe(IV)Fe(III) clusters, such as Q in methane monooxygenase and X in ribonucleotide reductase^29,36,37^, have distinct Mössbauer parameters, and the formation of a Fe(IV)-containing cluster from μ-peroxo-Fe(III)_2_ involves, to the best of our knowledge, irreversible O–O bond cleavage, the observations disfavour the involvement of such species. Thus, we propose that, in PolF, μ-peroxo-Fe(III)_2_ is responsible for H abstraction and that the preceding slow step is a physical event (Fig. 3d, second step).

### Post-H-abstraction reactions proceed by radical mechanisms

To obtain insights into the mechanisms of l-Val reactions after the H-abstraction step, we characterized the substrate D-KIE of the transformation of C3• into 3,4-dh-Val. This reaction involves transfers of an electron and a proton that proceed either stepwise or in concert (proton-coupled electron transfer, PCET). These mechanisms can be distinguished by determining the D-KIE of 4-H^+^ transfer by the intramolecular competition KIE. Thus, as described in Supplementary Note 1, we determined D-KIE of 4-H^+^ transfer as 1.3. This value is too small to reflect a KIE of a pure proton transfer (~7 for semiclassical KIE), while it is consistent with the reported orthogonal PCET (1.2–2.1)^38–41^, where the proton and electron are transferred to different acceptors. Thus, these results indicate a PCET mechanism for the 3,4-desaturation step. The observation also suggests that the oxidation of C3• to a cation by a pure electron transfer without proton transfer would not be kinetically feasible. These results provide important insights into the mechanism of post-H-abstraction reactions by PolF.

### l-Ile coordinates the diiron cluster of PolF

**Extended Data Fig. 5:**
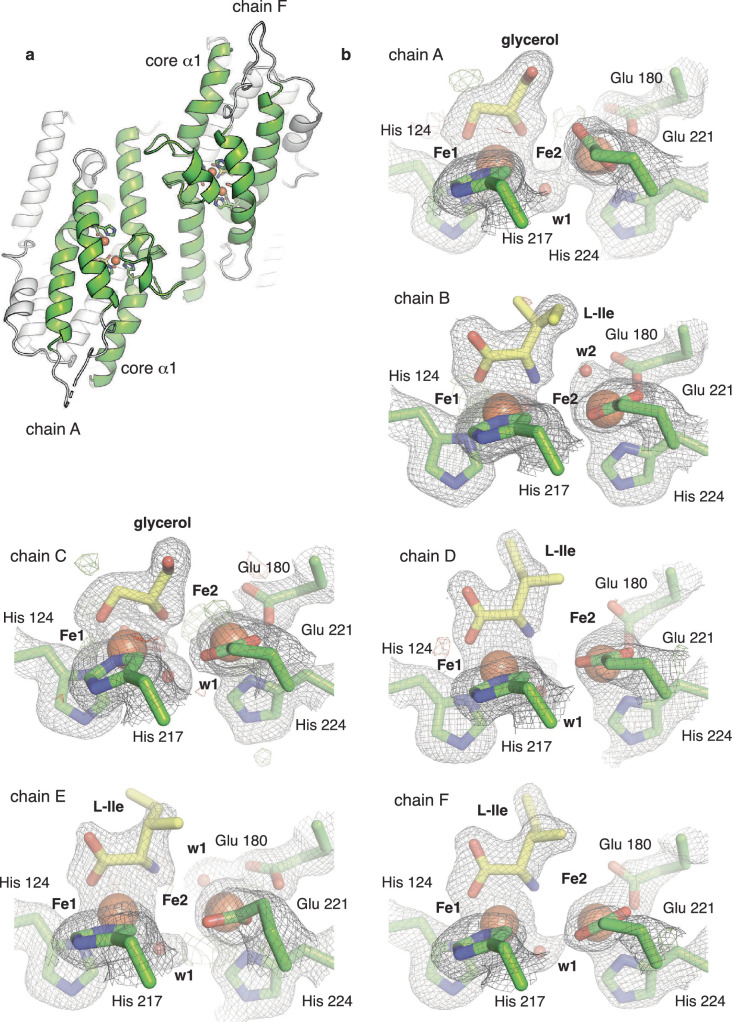
Views of the electron density map for PolF active site components in each of the six independent views provided in the asymmetric unit (ASU) of the Fe_2_(II/II)•l-Ile•PolF complex. (**a**) As in many other HDOs, PolF adopts a dimeric quaternary structure involving contacts between core α1 and aux α2. Interestingly, core α1 is interrupted by a long segment that adopts a loop-helix-loop structure. While other HDOs contain similar interruptions in this secondary structure, the PolF motif is the longest and most globular identified to date. In addition to composing the dimer, this segment also contacts core α3, potentially explaining why this component is ordered in all PolF crystals, even the mono-Zn(II) complex (Figure S23). (**b**) The ASU of Fe_2_(II/II)•l-Ile•PolF contains six copies, arranged as three of the dimers shown in panel **a**, offering six independent views of the metal-binding site. Selected amino acids are shown as sticks, water molecules are shown as red spheres, and iron ions are shown as orange spheres. 2*F*_o_-*F*_c_ maps are shown in gray mesh and contoured at 1.0σ. *F*_o_-*F*_c_ maps are shown in red/green mesh and contoured at ±3.0σ. All chains show strong metal ion (71-100%) occupancy. In some chains, the electron density associated with Fe1 can be best modelled as glycerol (chains A and C), present in both the protein storage buffer and cryoprotectant solution (10-20% of the solution). Glycerol is similar in size to l-Ile and we assessed both options to account for the extra electron density at Fe1. Attempts to model l-Ile in chains A and C resulted in negative difference density associated with the carboxylic acid motif and poor density for the ethyl moiety of the side chain, two distinctive features of the substrate that can be used to differentiate from glycerol. The remaining chains show strong density for the carboxylate of l-Ile but varying coverage of the side chain. The side chain is most well-defined in chains D and F. Interestingly, in chain B, the side chain models best in a different rotameric form. This difference could reflect a capacity to accommodate different rotamers. It could also reflect a mixture of l-Ile and glycerol in this chain. Chain F was judged to be the highest quality model of the l-Ile substrate with the smallest contribution from glycerol. Consequently, it is used as the representative view in all other figures.

**Extended Data Fig. 6:**
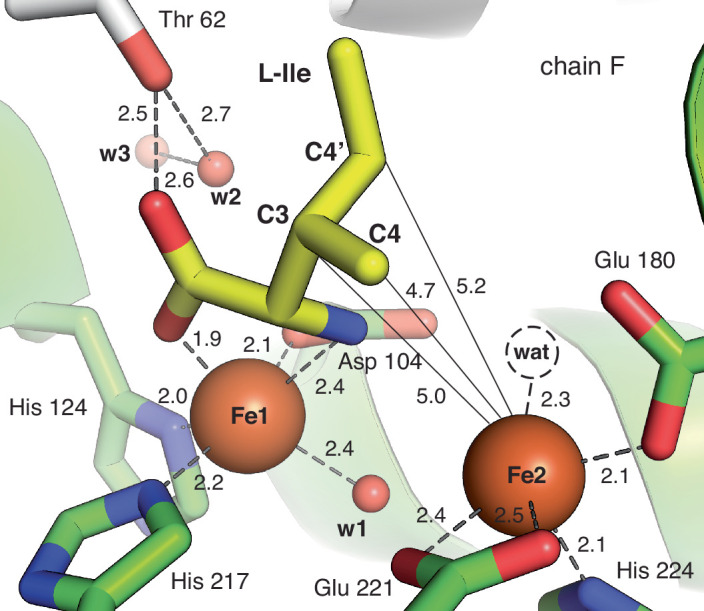
A map of key interactions and distances in a selected view (chain F) of the active site in the Fe2(II/II)•l-Ile•PolF complex. Chain F was selected because the electron density map for the l-Ile substrate is the most detailed and provides a basis to model the entire side chain with high confidence. Metal-ligand and hydrogen-bonding interactions are shown as dashed lines. Distances between Fe2 and potential C-H bonds targeted for H-atom abstraction are shown as solid lines. All distances are given in Å. A water molecule coordinated to Fe2 observed in other chains is shown as a dashed circle with representative bonding distance indicated. Unlike other Fe_2_(II/II)-HDO structures, PolF does not exhibit two adjacent open coordination sites on each metal ion that might delineate the position of a μ-1,2-peroxo complex, indicating that conformational changes might need to occur in the first coordination sphere to accommodate O_2_ addition. The coordination mode of l-Ile and observed positioning of its side chain locates C3 and C4/4’ within reasonable proximity of the diiron cluster for target by reactive intermediates. However, in this model, only C4/4’ are oriented appropriately to provide access to the relevant C-H bonds. Target of C3 would require rotation of the side chain or other conformational change. Experiments with deuterium isotopologs of l-Val indicate that all three carbon atoms can undergo HAT, but labeling at C4/4’ yields a more pronounced KIE on decay of the μ-1,2-peroxo complex, perhaps consistent with the more favorable positioning of the analogous atoms in the l-Ile complex.

To corroborate cofactor assignment and mechanistic proposals, we solved X-ray crystal structures of PolF in complex with Fe_2_(II/II) and the native l-Ile substrate. We initially solved a structure of apo-PolF, which we intended to be devoid of metal ions, but it was subsequently shown to be mismetallated by a Zn(II) centre in metal-binding site 1 (Supplementary Note 3 and Supplementary Figs. 22 and 23). To obtain structures with the catalytically relevant metal, we soaked the Zn(II)-bound crystals with Fe(II) and l-Ile, reasoning that the Zn(II) might be labile and readily displaced. The resulting X-ray diffraction datasets show high occupancy of both iron-binding sites, confirmed by anomalous diffraction data collection at the Fe X-ray absorption edge (Supplementary Fig. 22 and Extended Data Fig. 5). We also observed extra electron density associated with Fe1, which modelled well in certain chains as l-Ile (Fig. 4b and Extended Data Fig. 5). The l-Ile substrate directly coordinates Fe1 in a bidentate fashion (Extended Data Fig. 6), as observed in another substrate-triggered HDO, AetD^18,42^, which also modifies an amino acid substrate^17^. While a comparison of AetD and PolF substrate-bound complexes shows occupancy of a similar binding site (Fig. 4b,c), the rest of the PolF diiron coordination sphere differs significantly from that of AetD^18^ and other HDOs, such as SznF^43^. AetD contains a bridging carboxylate ligand and open or solvent-occupied coordination positions on each iron that project towards the substrate-binding cavity to delineate the possible site of the μ-peroxo intermediate. PolF is distinct in its lack of a bridging carboxylate. A coordinated water is bound to Fe1, but it is projected away from the substrate-binding site and axial to the l-Ile carboxylate. Fe1 is also coordinatively saturated with six metal-ligand interactions, while Fe2 is either four-coordinate or five-coordinate. This arrangement makes it challenging to envision how oxygen might add to the cofactor to yield the observed μ-1,2-peroxide complex without further conformational change in the first coordination sphere. Note that this proposed conformational change would occur before the aforementioned slow physical step that follows μ-1,2-peroxo complex formation and precedes H-atom abstraction from the substrate.

While we cannot predict the location of the peroxo ligand in the reactive intermediate state from the structure of the Fe_2_(II/II)•l-Ile•PolF complex, the binding mode of substrate projects the C3 and C4 atoms close to Fe2 of the diiron cofactor, 4.7–5.2 Å away from the site 2 metal (Extended Data Fig. 6), rationalizing the observed transformation of both positions in l-Ile and l-Val. The side chain of substrate projects into a hydrophobic pocket lined by both large aromatic and small aliphatic residues in PolF (Fig. 4b). The sole H-bonding contact in the substrate-binding pocket is Thr62, contributed by auxiliary helix 2, a common substrate-binding motif in HDOs. Thr62 is nearest to the l-Ile carboxylate and probably helps orient this ligating group. Thr62 also interacts with two water molecules in a short solvent channel that connects to the surface of the protein, providing a potential mechanism to shuttle protons in and out of the otherwise very hydrophobic substrate-binding site. By contrast, the coordinated α-amine of l-Ile remains completely untethered by second sphere H-bonding. The Fe1–N interaction is weaker as a consequence, with a longer distance and less well-defined electron density in some chains (Extended Data Figs. 5 and 6). This combination could promote the C4–N coupling of the second reaction by holding the N in close proximity to a C4 substrate carbon radical generated in the second round of chemistry.

### Desaturation of Ile by PolE assists PA production by PolF

The formation of multiple products in the PolF reaction with l-Ile is unusual. While PolE was not essential for the biosynthesis of PA-containing polyoxin A, the *ΔpolE* mutant produced reduced amounts of polyoxin A. Therefore, we proposed that PolE may have an assistant function to make the pathway more efficient or specific. While the DUF6421 family proteins, including PolE, show structural homology to Zn^2+^-dependent dipeptidyl-peptidases III (DPP III)^44^, the metal-binding motif, HExxGH^45,46^ and the catalytic His residue (His578 in yeast) of DPP III^44^, are not conserved. Instead, the putative metal-binding motif of DUF6421 (HD–ExxH) is homologous to that of the recently reported HExxH Fe and α-KG and Fe-dependent oxygenases^47^. However, the AlphaFold^48^ model of PolE did not show positively charged amino acid residues for the α-KG binding^47^. Instead, the two His residues in the HE/DxxH motif are clustered with Glu230, strictly conserved among DUF6421, which resembles that of Fe and pterin-dependent oxygenases (Supplementary Fig. 24). The reported Fe and pterin enzymes are aromatic amino acid hydroxylases^49^, mostly found in eukaryotes, and catalyse the activation of O_2_ to generate Fe^IV^-oxo intermediate^50^ while oxidizing tetrahydrobiopterin (BH_4_). The resulting Fe^IV^-oxo intermediate is used to catalyse hydroxylation of aromatic amino acids.

**Extended Data Fig. 7:**
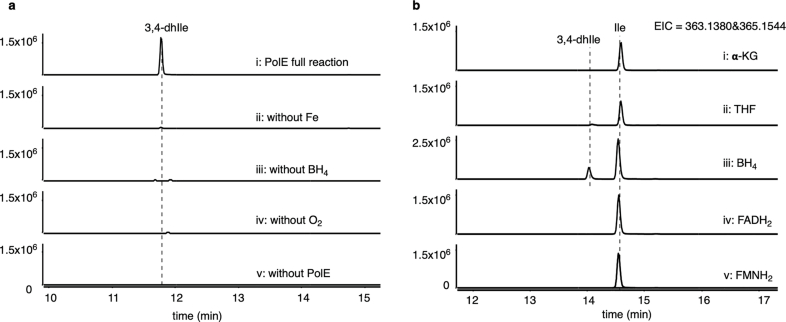
PolE activity assays. (**a**) LC-MS analysis (EIC at *m/z* 363.1373) of PolE assays. PolE assays were performed with 150 μM l-Ile, 15 μM PolE, 100 μM Fe(II), 1 mM BH_4_, 1 mM ascorbate and ~0.5 mM O_2_ (i). Also shown are controls without Fe (ii), without BH_4_ (iii), without O_2_ (iv), and without PolE (v). (**b**) LC-MS analysis of PolE assays with different cofactors (EIC at *m/z* 363.1373 and 365.1530). The assays were performed with 150 μM l-Ile, 15 μM PolE, 100 μM Fe(II), 1 mM ascorbate, and ~0.5 mM O_2_, with 1 mM α-KG (i), with 1 mM THF (ii), with 1 mM BH_4_ (iii), with 100 μM FAD and 1 mM NADH mimic (iv), and with 100 μM FMN and 1 mM NADH mimic (v). Source data are provided. Source data

On the basis of these considerations, we tested the catalytic function of recombinant PolE. When l-Ile was used as a substrate, PolE catalysed the desaturation of l-Ile in the presence of BH_4_ (Fig. 5a and Supplementary Fig. 25). The PolE reaction product was isolated and structurally characterized by NMR as *E*-3,4-dh-Ile (Supplementary Figs. 26 and 27). The observed activity was strictly dependent on Fe^2+^, O_2_ and BH_4_ (Extended Data Fig. 7). The activity was enhanced ~2–3 times in the presence of dihydropterin reductase (quinoid dihydropteridine reductase (QDPR), Supplementary Fig. 28). We also tested the functional significance of the HExxHx_n_E motif in PolE. While E203A-PolE was expressed as an insoluble protein, the other mutants were expressed as soluble proteins and exhibited no detectable l-Ile desaturation activity (Supplementary Fig. 29). These observations indicate that PolE is an Fe and pterin-dependent oxidase.

The PolE reaction with l-Ile was highly specific and yielded 3,4-dh-Ile as the only product (Fig. 5a). Furthermore, the observed activity was specific to l-Ile. While l-Val and l-Leu gave trace amounts of desaturated products (<5% of l-Ile reaction), no other proteinogenic amino acids served as substrates (Supplementary Fig. 30). Thus, in contrast to PolF, PolE catalyses a highly specific desaturation of l-Ile.

**Extended Data Fig. 8:**
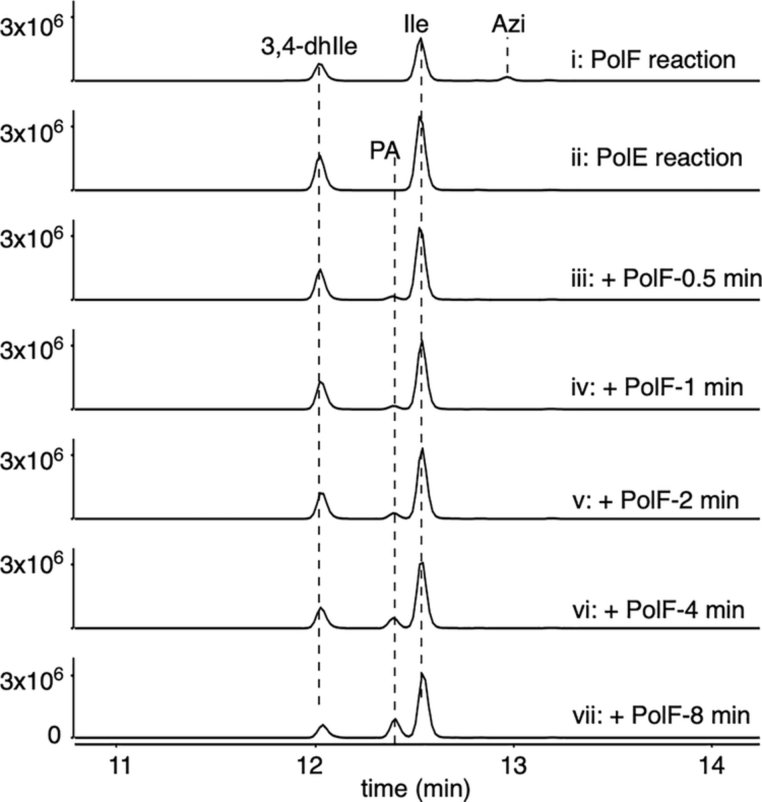
PolEF stepwise assay. LC-MS analysis of PolF single turnover reaction (i), PolE assay (ii), PolE and PolF stepwise assay (iii – vii). The PolE assay was performed with 150 μM l-Ile, 15 μM PolE, 100 μM Fe(II), 100 μM BH_4_, 1 mM ascorbate, 1 mM NADH, 0.5 μM QDPR and ~0.5 mM O_2_ at room temperature for 40 min. In the stepwise assays, 15 μM PolF was added to the PolE assay mix after the initial 40 min incubation. The reactions were quenched by equal volume of acetonitrile at 30 s (iii), 1 min (iv), 2 min (v), 4 min (vi) and 8 min (vii). Shown are EIC at *m/z* 363.1373 and 365.1530 (i) or at *m/z* 361.1217, 363.1373 and 365.1530 (ii - vii). Source data are provided. Source data

The 3,4-dh-Ile produced by PolE was indistinguishable from the dh-Ile produced by PolF (Extended Data Fig. 8) and was efficiently converted to PA by PolF (Fig. 5b and Supplementary Fig. 31). When PolE and PolF were co-incubated with l-Ile as the substrate under multiple turnover conditions (Fig. 5c and Supplementary Fig. 31), the reaction yielded PA with minimal formation of Azi and 4-OH-Ile. The rate of PA formation in the PolE•PolF coupled assay (0.16 min^−1^) was ~3 times faster than that in the PolF-only assay (0.052 min^−1^). On the other hand, the rate of l-Ile desaturation under the single turnover conditions is comparable between PolE and PolF, suggesting that the difference in rate of PA formation by PolF alone is probably limited by the slow re-reduction of the diiron cluster. The combined results indicate that in the presence of PolE, the PA formation from l-Ile becomes more efficient and specific. These observations are consistent with the results of the gene knockout experiments and suggest that while PolF is sufficient for PA biosynthesis, PolE makes the biosynthetic pathway more efficient and specific (Fig. 5d).

## Discussion

Azetidine is one of the most important structural motifs for medical and organic chemistry research^2,3^. The previously reported mechanisms of azetidine biosynthesis (Extended Data Fig. 1) require metabolically or chemically expensive substrates. In contrast, the current report elucidates a previously unseen mechanism of azetidine biosynthesis, which requires only one enzyme, PolF, with inexpensive amino acids (l-Ile or l-Val) as the substrates. Considering the simple nature of this enzymatic system, PolF could be a new biocatalyst for azetidine amino acid synthesis.

Our mechanistic study of PolF catalysis provides detailed insights into the mechanism of radical initiation by HDO enzymes. PolF showed a substantial apparent D-KIE of 3.1–3.6, one of the largest effects reported for HDOs, without any detectable uncoupling, making it an ideal system for characterizing the H-abstraction step. In addition, PolF catalyses a highly challenging C–H activation. Many enzymes in this family catalyse oxidative reactions that do not require C–H activation or cleavages of relatively weak C–H bonds. Since other non-haem Fe-dependent enzymes that catalyse unactivated C–H bond cleavage use high-valent Fe(IV)-containing cluster, the function of PolF raised a question of whether μ-peroxo-Fe(III)_2_ is responsible for the H abstraction. Our combined stopped-flow and Mössbauer spectroscopy results indicate that μ-peroxo-Fe(III)_2_ is responsible for H abstraction and eliminates kinetically and spectroscopically resolvable intermediates between μ-peroxo-Fe(III)_2_ and H abstraction. These observations constitute comprehensive experimental evidence that a μ-peroxo-Fe(III)_2_ is directly responsible for the chemically challenging C–H activation of an aliphatic methyl group (bond dissociation energy up to 101 kcal mol^−1^) in PolF.

The difference between the apparent and intrinsic D-KIE of the 3-H and 4-H abstraction (3.1–3.6 versus 25 and 36) suggested the presence of a slow rate-determining step preceding the H abstraction, which we assigned to be a physical (non-chemical) step. While the nature of this event requires further investigation, one possibility involves a re-orientation of the substrate so that the target C–H bond is appropriately presented to the μ-peroxo-Fe(III)_2_ for H abstraction. Alternatively, this event may be related to a structural transition of the active site or the Fe_2_ cofactor from the O_2_ activation to C–H activation. This physical step may be a part of the mechanism to activate the otherwise relatively weak oxidant, μ-peroxo-Fe(III)_2_. While the nature and catalytic function of conformational gating in PolF require further investigation, the results revealed a previously uncharacterized step in HDO catalysis.

**Fig. 6:**
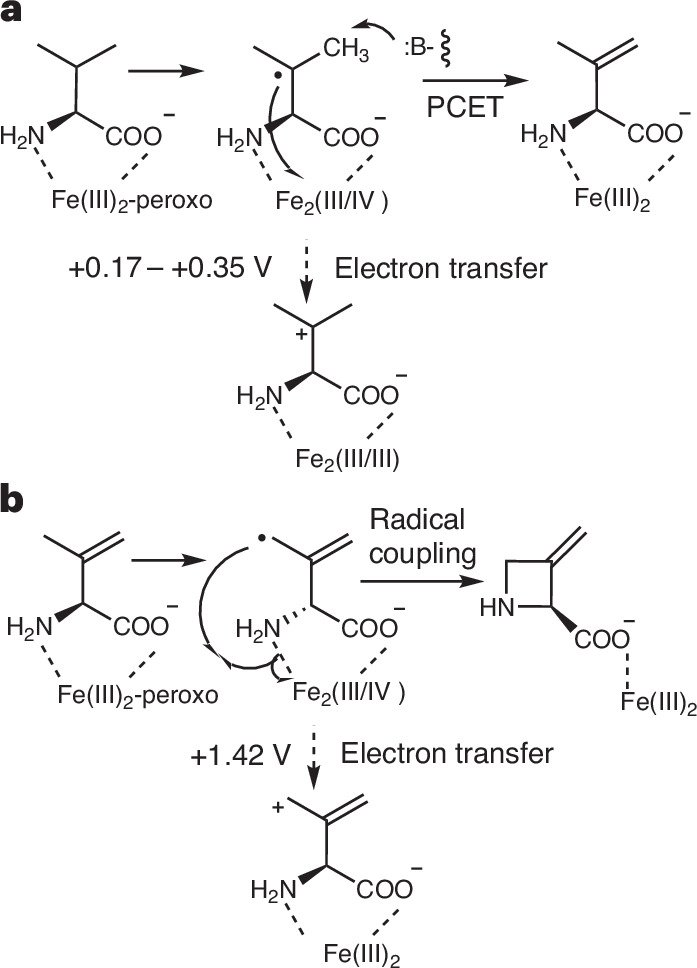
Mechanism of C–N bond formation. **a**, The proposed mechanism of 3,4-desaturation. **b**, The proposed mechanism of azetidine formation. The oxidation of C3• or allylic C4• to a carbocation (dashed arrows) is unlikely to be kinetically feasible.

The azetidine formation by PolF is a new catalytic function among HDO or any O_2_-activating Fe enzymes, while three-membered ring aziridine formation from l-amino acids is precedented by the α-KG/Fe-dependent enzyme, TqaL^51,52^. The proposed mechanism of TqaL involves H-atom abstraction to form a C3• intermediate, oxidation of C3• to a cation and a nucleophilic attack of 2-NH_2_ to form the C–N bond (Supplementary Fig. 32). For PolF, our KIE analysis suggests that the l-Val desaturation proceeds through PCET (Fig. 6a), suggesting that the oxidation of C3• to cation without a proton transfer (+0.17–+0.35 V versus NHE)^53,54^ is not kinetically feasible. Since the oxidation of an allylic radical is even more difficult (~+1.4 V versus NHE)^53,54^, the azetidine formation is unlikely to proceed through a cation intermediate. A recent characterization of an engineered flavoenzyme *Ac*HYAM suggested that a C–N bond can form between anillin nitrogen and benzyl radical through a nitrogen lone pair-assisted oxidation of a benzyl radical^55^ (Supplementary Note 4). In the Fe_2_(II/II)•l-Ile•PolF structure, the α-NH_2_ is coordinated to the diiron cluster. Thus, if the C–N bond formation in PolF proceeds by a nitrogen lone pair-assisted radical oxidation, the α-NH_2_ must dissociate from Fe to make the nitrogen lone pair available for interaction with the allylic radical. Thus, while we cannot eliminate such a possibility, we propose an alternative mechanism in which the allylic C4• adds directly to the α-NH_2_ (Fig. 6b). Further studies are needed to distinguish these possible catalytic mechanisms.

**Extended Data Fig. 9:**
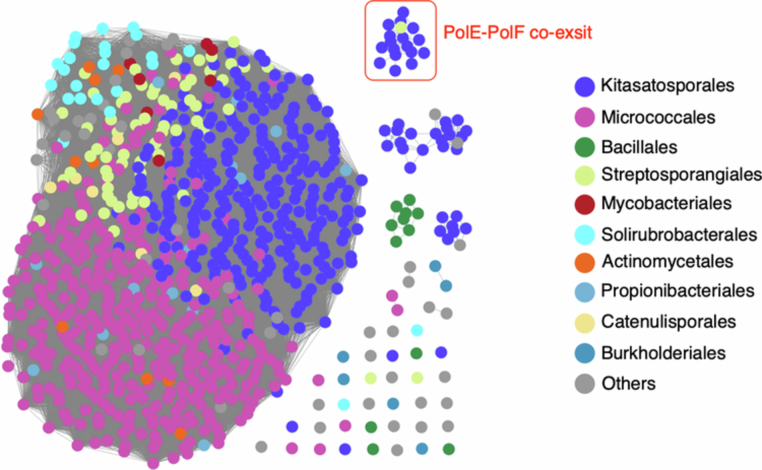
SSN of HE/DXXH Fe/pterin-dependent O_2_ activating enzyme family. The alignment score of 150 was used as the edge threshold.

We also found that PolE is an Fe/pterin-dependent oxidase and catalyses a highly specific desaturation of l-Ile. On the basis of the gene knockout and enzymological studies, we propose that PolE helps PolF catalysis by increasing the flux of l-Ile desaturation. PolE does not show sequence or structural homologies to the previously reported Fe/pterin-dependent enzymes. On the basis of position-specific iterative BLAST search on the National Center for Biotechnology Information (NCBI) database, we identified ~10,000 homologues of PolE, which are mostly annotated as DUF6421, suggesting this protein family is large in size. Analysis of DUF6421 UniRef90 sequences in the UniProt database (Extended Data Fig. 9) revealed that these proteins are found exclusively in bacteria, primarily in actinobacteria. Their multiple sequence alignment shows that they share the conserved HE/DXXHX_(21__–23)_E motif, probably responsible for the Fe and BH_4_ binding (Supplementary Fig. 33). Therefore, we propose that they form a new family of Fe and/or pterin-dependent enzymes. Many of these enzymes colocalize with other putative amino acid-modifying enzymes. Thus, these results indicate the presence of previously unappreciated, diverse pathways that produce unusual amino acids.

## Methods

### General

All reagents were purchased from commercial sources without further purification. NMR spectra were collected on an 800-MHz Varian NMR and a 700-MHz Bruker NMR housed within the Duke University NMR Spectroscopy facility. NMR spectra were processed and analysed with Mnova NMR Software v.14.0.0 (Mestrelab). Anaerobic sample preparation was performed in a glove box (MBraun) maintained at [O_2_] < 0.1 ppm. High-performance LC (HPLC) analyses and purifications were conducted on a Hitachi D-2000 Elite system consisting of an L-2130 pump, L-2300 column oven, L-2200 autosampler, L-2455 diode array equipped with a ODS Hypersil column (3 μm, 4.6 × 150 mm, Thermo Fisher Scientific). LC with high-resolution mass spectrometry (LC–HRMS) data were collected with an Agilent Technologies 1290 Series LC and an Agilent Technologies 6545 electrospray ionization quadrupole time-of-flight (ESI-Q-TOF) MS with dual ESI source and a HPH C18 column (2.7 μm, 2.1 × 100 mm, Agilent), a C18 column (1.8 μm, 2.1 × 50 mm, Agilent) or an ACQUITY BEH Amide column (1.7 μm, 2.1 × 100 mm, Waters). Size-exclusion column chromatography analyses were conducted on an AKTA purifier (GE Healthcare Life Sciences) with a Superdex 200 10/300 GL column (GE Healthcare Life Sciences, part number 17-5175-01). Stopped-flow experiments were performed at 5 °C using an SX20 stopped-flow spectrophotometer equipped with a photodiode-array detector (Applied Photophysics Ltd) housed in an MBraun chamber. All the proteogenic amino acids and DnsCl were purchased from Sigma-Aldrich. 3-OH-Val and 4-OH-Val were purchased from Ambeed. 3,4-dh-Val was purchased from A2Bchem. Ni-(*S*)-BPB-Gly was purchased from Ambeed. 3,4-dh-Val was purchased from A2Bchem. [1,3-D_6_]-2-bromopropane was purchased from AccelaChemBio. [3-D]Val, [U-D_8_]Val and ^13^C_6_-Ile were purchased from Cambridge Isotope Laboratories Restriction enzymes (NEB) were used according to the instructions provided by the manufacturers. QDPR was purchased from VWR. PCR amplification of DNA fragments was performed using Q5 High-Fidelity DNA polymerase (NEB).

### Analysis of metabolites of *S. cacaoi* wild type and mutants

Here 10 μl of spore suspension (10^8^–10^9^ CFU) of *S. cacaoi* strains were inoculated in tryptic soy broth medium total 10 ml) in 50-ml tube with 1–2 cm of spring coil and incubated at 28 °C with shaking at 225 rpm for 2–3 d. The resulting *Streptomyces* liquid cultures (0.5–2 ml) were then diluted in 50 ml of the fermentation medium N containing 100 mM PIPES buffer pH 6.0 in 250-ml baffled flasks containing stainless steel springs with the starting optical density at 600 nm of 0.1 (ref. ^11^). The cultures were incubated at 28 °C with shaking at 225 rpm. During the fermentation, the pH was monitored. The pH was at ~6.5 between days 1–5, and increased to ~7.0 on day 6–7. After 5–7 d of culture, the culture media were collected, cleared by centrifugation at 15,000*g*, 4 °C for 20 min to remove mycelia and analysed by LC–MS. Supernatant samples were made to 20% MeCN (final concentration), filtered through Sep-PakTM Plus C18 cartridges (Waters). The flow-through samples were collected and cleared once again by centrifugation at 15,000*g*, 4 °C for 10 min and the cleared supernatants were subjected to LC–MS or LC–MS/MS analysis. Polyoxins were chromatographed on a ACQUITY Premier BEH Amide 1.7-μm Vanguard Fit, 2.1 × 100 mm column at 40 °C using solvents A (10 mM NH_4_OAc pH 10.0) and B (acetonitrile), with a linear gradient of 15–50% for A for 30 min at a flow rate of 0.5 ml min^−1^. The elution was monitored by ultraviolet (UV) absorption at 260 nm as well as ESI-TOF MS. The MS data were analysed by MassHunter (Agilent Technologies).

### PolF activity assay

For the multiple turnover condition, the assay with l-Ile (or other l-amino acids) was performed at 25 °C by 1:1 mixing of a 100-μl solution containing apo-PolF (60 μM), substrate (600 μM), ascorbate (2 mM) and Fe^II^(NH_4_)_2_(SO_4_)_2_ (200 μM) in 50 mM HEPES-NaOH pH 7.6 with a 100 μl of O_2_-saturated buffer B (1 mM at room temperature). The mixture was incubated at 25 °C for 2 h, after which the reaction was quenched by mixing with 200 μl of acetonitrile. To an aliquot (60 μl) of the quenched reaction mixture was added 10 μl of 1 M borate (pH 8.0) and 10 μl of 20 mM DnsCl (in acetonitrile). After incubating at 25 °C for 1 h, the mixture was centrifuged and 2 μl of the supernatant was injected into and analysed by LC–MS equipped with a Poroshell HPH C18 column (2.7 μm, 2.1 × 100 mm, Agilent) at 40 °C using solvents A (10 mM NH_4_OAc pH 10.0) and B (acetonitrile): 0–3 min, 3% B; 3–6 min, 3–10% B linear gradient; 6–21 min, 10–60% B linear gradient; 21–23 min, 60–95% B linear gradient; 23–26 min, 95% B; 26–27 min, 95–3% B linear gradient; and 27–30 min, 3% B. The flow rate was set to 0.5 ml min^−1^. The elution was monitored by UV absorption at 325 nm, and ESI-TOF MS. The MS data were analysed by MassHunter (Agilent Technologies).

For the single turnover condition, the assays with l-Ile (or other l-amino acids) were performed at 4 °C by 1:1 mixing of a 100 μl of solution containing apo-PolF (300 μM), substrate (1 mM) and Fe^II^(NH_4_)_2_(SO_4_)_2_ (0.6 mM) in 50 mM HEPES-NaOH pH 7.6 with a 100-μl O_2_-saturated buffer B (1.8 mM at 4 °C). The mixture was incubated at 4 °C for 5 s, 10 s, 20 s, 30 s, 1 min, 2 min, 4 min and 10 min. At each time point, the reaction was quenched by mixing 15 μl of the reaction solution with 9.4 μl of 2% HClO_4_. This mixture was neutralized by adding 7.5 μl of 0.5 M KOH, followed by the addition of 1 μl of 1.19 mM [U-D_8_]Val (1.14 mM of l-Val was added for [U-D_8_]Val reaction) and 1 μl of 1.36 mM l-Thr as the internal standard. The resulting solution was flash frozen in liquid nitrogen to precipitate potassium perchlorate. After defrosting on ice, the solutions were centrifuged at 4 °C for 20 min to remove the potassium perchlorate and precipitated proteins. An aliquot of the supernatant (30 μl) was derivatized by adding 30 μl of acetonitrile, 10 μl of 1 M borate (pH 8.0) and 10 μl of 20 mM DnsCl. After incubating at 25 °C for 1 h, 2 μl of the supernatant was analysed by LC–MS described above. The products were quantified by comparing the peak area in the extracted ion chromatograms (EICs) with the internal standards. To calculate the rate of H-atom abstraction, the total amount of all the products was plotted against the incubation time and fit the data to equation (1).

1 $$P(t)={{A}_{0}-A{\rm{e}}}^{-kt}$$

To determine the rate constant of each product formation, the kinetic trace for each product was fit to equation (1). Then, the fraction of each product formation was calculated on the basis of the ratio of the amplitude of each product formation and the sum of the amplitudes of all the products. Then, the rate constant for the individual product formation was calculated by dividing the rate constant of all products by the fraction of each product formation.

### Stopped-flow experiments

Stopped-flow experiments were performed by an 1:1 mixing of solutions A and B: solution A (600 μl) constituted of 300 μM apo-PolF, 1 mM substrate and 0.6 mM Fe^II^(NH_4_)_2_(SO_4_)_2_ in anaerobic 50 mM HEPES-NaOH pH 7.6; and solution B (600 μl) was O_2_-saturated HEPES buffer (1.8 mM at 4 °C). Stopped-flow experiments were carried out at 5 °C in the MBraun glove box. The stopped-flow apparatus was equipped with a photodiode-array detector with a 1-cm path length and configured for single mixing. Time-dependent absorption spectra (1,000 per trial) were acquired with a logarithmic time over 1,000 s.

The *A*_614nm_-versus-time trace from each of these experiments showed a biphasic rise and monophasic decay, which are similar to BesC and SznF^25,56^. For fitting the kinetic data, we used an extended form of linear regression equation (1)^56^.

2 $$A(t)={A}_{0}+\,\Delta A\left(\frac{{k}_{1}}{{k}_{2}-{k}_{1}}\right)\left({{\rm{e}}}^{-{k}_{1}t}-{{\rm{e}}}^{-{k}_{2}t}\right)+\Delta A{\prime} \left(\frac{{k}_{1}{\prime} }{{k}_{2}-{k}_{1}{\prime} }\right)\left({\mathrm{e}}^{-{k}_{1}{\prime} t}-\,{\mathrm{e}}^{-{k}_{2}t}\right)$$

### Freeze-quenching and Mössbauer experiments

Preparation of freeze-quenched Mössbauer samples was performed by following previously published protocols^57^. The O_2_ stable and acidic ^57^Fe(II) stock solution was prepared from commercial ^57^Fe(0), as previously described^58^, and diluted to 20 mM in the anoxic chamber by mixing with 50 mM HEPES-NaOH (pH 7.6) buffer containing 150 mM NaCl and 10% glycerol. The reactant complexes, PolF•^57^Fe(II)_2_•[U-D_8_]Val or PolF•^57^Fe(II)_2_• l-Val, were prepared from anoxic solutions of 1.44 mM PolF, 2.88 mM ^57^Fe(II) and 40 mM l-Val or [U-D_8_]Val. These solutions were rapidly mixed at 5 °C with an equal volume of O_2_ saturated buffer (~1.8 mM) and allowed to incubate for the respective reaction times indicated in Fig. 2d and Extended Data Fig. 4. Following incubation, samples with reaction times from milliseconds to seconds were frozen by injection into cold (−150 °C) 2-methylbutane. The anoxic control (no dilution) and samples with reaction times of several minutes were pipetted into Mössbauer cells and quickly frozen on a liquid nitrogen-cooled metal block. Mössbauer spectra were recorded on a spectrometer from SEE Co. equipped with a Janis SVT-400 variable-temperature cryostat. The reported isomer shift is given relative to the centroid of the spectrum of α-iron metal at room temperature. External magnetic fields were applied parallel to the direction of propagation of the γ radiation. Simulations were performed using the software WMOSS from SEE Co. (www.wmoss.org, SEE Co.).

### X-ray crystallographic characterization of PolF

In an attempt to isolate the apo form of N-terminally His_6_-tagged PolF, the enzyme was overexpressed in minimal medium and purified by Ni-NTA affinity chromatography as previously described^56^. The eluted fractions were treated overnight at 4 °C with 10 mM EDTA in 20 mM HEPES, pH 7.6, 50 mM NaCl, 10% glycerol to remove adventitiously bound metal ions. PolF was also purified by anion-exchange chromatography using a HiPrep QFF column controlled by an AKTA Pure FPLC system (GE Healthcare) at 4 °C. Samples were loaded in 10 mM Tris, pH 8.0, 10 mM NaCl and 10% glycerol and eluted at 250 mM NaCl when a gradient of 10 mM–1 M NaCl (in 10 mM Tris, pH 8.0, 10% glycerol) was applied over 25 column volumes. PolF was subsequently subjected to size-exclusion chromatography by using a HiLoad 16/600 Superdex 200 gel filtration column at 4 °C. The mobile phase was 20 mM sodium HEPES (pH 7.6) buffer, 200 mM NaCl and 10% glycerol at 0.5 ml min^−1^. Purified protein samples were exchanged into a storage buffer of 20 mM HEPES, pH 7.6, 50 mM NaCl and 10% glycerol and flash frozen in liquid nitrogen for long-term storage at −80 °C. A tagless version of PolF was also generated by incorporation of a tobacco etch virus (TEV) cleavage site using standard site-directed mutagenesis protocols. After the Ni-NTA affinity chromatography step, pooled PolF fractions were treated overnight at 4 °C with TEV protease (1 mg:50 mg protein) in 20 mM HEPES, pH 7.6, 50 mM NaCl, 10% glycerol. The mixture was applied to a Ni-NTA column to remove the His-tag fragment. The eluent was subjected to EDTA treatment, dialysis and gel filtration chromatography, as described above.

His_6_-tagged PolF was initially crystallized in its intended apo form by using the hanging drop vapour diffusion method. Protein samples were diluted to 8 mg ml^−1^ in the aforementioned storage buffer and mixed with an equal volume of a precipitant solution containing 24.5% (*w/v*) PEG 4000, 0.2 M (NH_4_)_2_SO_4_ and 0.1 M tri-sodium citrate, pH 6.3. The crystals grew to full size within a week. Crystals were harvested in rayon loops, soaked for 2 min in perfluoropolyether cryogenic oil and flash frozen in liquid nitrogen.

For structure solution of the iron-bound and substrate-bound complex, TEV-cleaved PolF samples were degassed and equilibrated in an anoxic chamber (Coy Laboratory Products) before crystallization. Protein solutions were prepared at 8 mg ml^−1^ in storage buffer containing 15 mM l-Ile and mixed with an equal volume of a precipitant solution containing 15% (*w/v*) PEG 6000, 0.5 M LiCl and 0.1 M Tris, pH 8.5. Crystals appeared within 1 week. Iron was incorporated via transfer of the crystals to a cryoprotectant solution containing the precipitating solution supplemented with 10 mM Fe(NH_4_)_2_(SO_4_)_2_, 12 mM l-Ile and 15% (v/v) glycerol. Crystals were soaked for 2 min before mounting on rayon loops and flash frozen in liquid nitrogen.

Crystallographic datasets were collected at the highly automated macromolecular crystallography (AMX) beamline at the National Synchrotron Light Source II (NSLS-II) (Brookhaven National Laboratory) or at the Stanford Synchrotron Radiation Light Source and processed with AutoPROC^59^. The Fe_2_(II/II)•l-Ile•PolF native dataset exhibited anisotropy and was additionally processed with the STARANISO server^60^. Phases were obtained by molecular replacement using the Phenix software package^61^. An AlphaFold2^48^ model of PolF was used as the initial search model in solution of the apo-PolF structure. The apo-PolF X-ray structure was used as the search model in phasing the l-Ile•Fe_2_(II/II)-PolF structure. Subsequent manual model building was carried out in Coot^62^. Refinement procedures were performed in Phenix. Coordinates were analysed for Ramachandran outliers and other geometric parameters using Molprobity^63^ and the wwPDB validation server.

The apo-PolF crystals belong to the *P*2_1_2_1_2_1_ space group with six monomers in the unit cell (Supplementary Table 5). The final model of apo-PolF consists of residues 7–275 in chain A; 7–275 in chain B; 6–200, 202–275 in chain C; 5–223, 234–275 in chain D; 6–200, 204–275 in chain E; 9–201, 203–275 in chain F, and 284 water molecules. Although the original protein sample was intended to be devoid of metal ions, the model also contains six Zn(II) ions, one occupying the active site in each chain of the enzyme, with the metal ion identity verified by anomalous diffraction data collection at the Zn K-edge X-ray absorption energy peak.

The l-Ile•Fe_2_(II/II)-PolF crystals belong to the *P*2_1_ space group with six monomers in the unit cell (Supplementary Table 6). The final model of apo-PolF consists of residues 7–200, 203–275 in chain A; 7–275 in chain B; 7–200, 204–275 in chain C; 7–275 in chain D; 6–275 in chain E; 12–275 in chain F, 140 water molecules, 12 iron ions, 11 Zn ions, 2 glycerol molecules and 4 l-Ile molecules. The metal ion identities were verified by anomalous diffraction data collection at the Fe *K*-edge X-ray absorption energy peak, indicating that Fe(II) can displace the adventitiously bound Zn(II) at site 1 detected in the apo-PolF crystals.

### PolE activity assay

For the multiple turnover condition, the assay with l-Ile (or other l-amino acids) was performed at 25 °C by 1:1 mixing of a 100-μl solution containing PolE or mutants (30 μM), substrate (300 μM), ascorbate (2 mM) and Fe^II^(NH_4_)_2_(SO_4_)_2_ (200 μM), BH_4_ (2 mM) in 50 mM HEPES-NaOH pH 7.6 with a 100 μl of O_2_-saturated buffer B (1 mM at room temperature). The assays with QDPR were performed similarly with 200 μM BH_4_, in the presence of 2 mM NADH and 1 μM QDPR. The mixture was incubated at 25 °C for 40 min, after which the reaction was quenched by mixing with equal volumes of acetonitrile. To an aliquot (60 μl) of the quenched reaction mixture was added 10 μl of 1 M borate (pH 8.0) and 10 μl of 20 mM DnsCl (in acetonitrile). After incubating at 25 °C for 1 h, the mixture was centrifuged and 2 μl of the supernatant was analysed by LC–MS equipped with a Poroshell HPH C18 column (2.7 μm, 2.1 × 100 mm, Agilent) at 40 °C using solvents A (water with 0.3% formic acid) and B (acetonitrile with 0.3% formic acid): 0–2 min, 3% B; 2–4 min, 10% B linear gradient; 4–19 min, 10–60% B linear gradient; 19–20 min, 60–95% B linear gradient; 20–25 min, 95% B; 25–25.5 min, 95–3% B linear gradient; and 25.5–27 min, 3% B. The flow rate was set to 0.5 ml min^−1^. The elution was monitored by UV absorption at 325 nm, and ESI-TOF MS. The MS data were analysed by MassHunter (Agilent Technologies).

## Online content

Any methods, additional references, Nature Portfolio reporting summaries, source data, extended data, supplementary information, acknowledgements, peer review information; details of author contributions and competing interests; and statements of data and code availability are available at 10.1038/s41557-025-01958-x.

## Supplementary information

Supplementary Information Supplementary Notes 1–4, Methods, Tables 1–6, Figs. 1–33 and uncropped gels.

## Source data

Source Data Fig. 1 Fig. 1d,e,g,h,i statistical source data.

Source Data Fig. 2 Statistical source data for stopped flow.

Source Data Fig. 3 Fig. 3a–c statistical source data. Fig3e,f statistical source data for stopped flow.

Source Data Fig. 4 Electron density map and PDB file.

Source Data Fig. 4 Electron density map and PDB file.

Source Data Fig. 5 Fig. 4a–c statistical source data.

Source Data Fig. 1 Fig. 1a–c original HPLC figures.

Source Data Extended Data Fig. 2 Extended Data Fig. 2a,b original HPLC figures.

Source Data Extended Data Fig. 3 Extended Data Fig.3a–d statistical source data for stopped flow.

Source Data Extended Data Fig.7 Extended Data Fig. 7a,b original HPLC figures.

Source Data Extended Data Fig.8 Extended Data Fig. 8 original HPLC figures.

## Data Availability

The NCBI accession numbers of the PolF and PolE sequences used in this study are ABX24498 and ABX24499, respectively. Atomic coordinates and structure factors for the crystal structures reported in this work have been deposited to the Protein Data Bank (PDB) under accession no. 9MRI (PolF with Zn^2+^) and 9PRR (PolF with Fe^2+^ and l-Ile). We also used the AetD crystal structure (PDB ID 8TWW) for structural comparisons. Other relevant data supporting the findings of this study are available in the article or its Supplementary Information. Source data are provided with this paper.

## Extended data

**Extended Data Table 1:** PolF assays with various amino acids^a^

	L-Ile	L-Val	L-Leu	L-Met	L-*allo*-Ile	D-Ile	D-*allo*-Ile	L-ABA
**Azetidine (-4 Da; PA or MAA)**	58 ^b^	39 ^b^	0	0	6 ^b^	7 ^b^	6 ^b^	0
**Desaturation (-2 Da)**	6 ^c^	4 ^b^	5 ^c^	11 ^c^	15 ^c^	3 ^c^	12 ^c^	30 ^c^
**Hydroxylation (+16** **Da)**	20 ^c^	22 ^b^	13 ^c^	18 ^c^	20 ^c^	27 ^c^	12 ^c^	17 ^c^

^a^The table shows the yield (%) of detected products after 1 h incubation of assays with substrates (Supplementary Fig. 10 and 11). The yields were calculated based on the integration of UV peak areas for the observed products.

^b^The identities of azetidines (PA and MAA) and l-Val derivatives (see Fig. 3) were confirmed by comparing the retention time and molecular weight with authentic standards. The authentic standards were purchased from a commercial source (3,4-dh-Val) or isolated and structurally characterized by NMR from large-scale assays (PA and MAA).

^c^The structures of the other products were deduced from the observed molecular weight (-2 Da for desaturation or +16 Da for hydroxylation).
