# Supplementary material for: Azetidine amino acid biosynthesis by non-haem iron-dependent enzymes
Source: Nat Chem. 2025 Oct 21;18(3):492–501. doi: 10.1038/s41557-025-01958-x (PMC12614342; doi:10.1038/s41557-025-01958-x)
Supplement: Supplementary file 1 — Supplementary Notes 1–4, Methods, Tables 1–6, Figs. 1–33 and uncropped gels. [file 41557_2025_1958_MOESM1_ESM.pdf]

nature chemistry

Article https://doi.org/10.1038/s41557-025-01958-x

Azetidine amino acid biosynthesis by non-haem iron-dependent enzymes

In the format provided by the
authors and unedited

Table of Contents

Supplementary Notes **3**

Note S1. Determination of intrinsic D-KIE by intramolecular competition. 3

Note S2. Determination of the rate constants of the H-abstraction steps. 4

Note S3. X-ray crystal structure of Zn(II)-bound PolF. 5

Note S4. Comparison with other C-N bond formation reactions. 6

Supplementary Methods **7**

Construction of *polE* and *polF* in-frame deletion mutants. 7

Cloning of PolF. 8

Expression and purification of PolF and PolE (Mutants). 8

Hydrolysis of polyoxin A. 9

Large-scale PolF reaction with L-Ile (L-Val) to purify PA (MAA). 9

[4,4'-D6]Val synthesis 10

Large scale PolE reaction with L-Ile to purify dh-Ile (^{13}C-dh-Ile). 10

Supplementary tables **12**

Table S1. Oligonucleotide primers used in this study. 12

Table S2. Plasmid DNA used in this study. 13

Table S3. Kinetic parameters from non-linear curve fitting of the single-turnover product formation kinetics data. 14

Table S4. Kinetic parameters from non-linear curve fitting of stopped-flow kinetics data. 15

Table S5. Data collection and refinement statistics for x-ray structures of Zn(II)-PolF. 16

Table S6. Data collection and refinement statistics for x-ray structures of L-Ile•Fe_2(II/II)-PolF. 17

Supplementary Figures **18**

Figure S1. Deletion mutation of the polE, and polF genes. 18

Figure S2. Examples of reactions catalyzed by HDOs 19

Figure S3. His-tagged PolF and PolE (mutants) purification. 20

Figure S4. Full retention time range of the chromatograms in Fig. 2b and Fig. 2c. 21

Figure S5. HPLC-MS characterization of PA. 22

Figure S6. NMR characterization of Dns-PA at 800 MHz in d6-DMSO 25

Figure S7. HPLC UV chromatograms (325 nm) of PolF assays with different reductants. 26

Figure S8. HPLC UV chromatograms (325 nm) of PolF assays with different metal ions. 27

Figure S9. NMR characterization of Dns-MAA at 800 MHz in d6-DMSO 29

Figure S10. HPLC UV chromatograms (325 nm) of PolF assays with proteogenic amino acids. 31

Figure S11. HPLC UV chromatograms (325 nm) of PolF reaction with Ile stereoisomers. 32

Figure S12. HPLC analysis of a time course of PolF reaction with L-Val under multiple turnover conditions. 33

Figure S13. HPLC analysis of a PolF reaction with 3,4-dhVal. 34

Figure S14. HPLC analysis of a PolF reaction with 4-OH-Val. 35
Figure S15. Characterization of a PolF reaction with Azi. 36
Figure S16. HPLC analysis of a PolF reaction with 3-OH-Val. 37
Figure S17. HPLC analysis of a PolF reaction with L-Ile under multiple turnover conditions. 38
Figure S18. LC-MS analysis of products from PolF reaction with L-Val and its isotopologs. 40
Figure S19. Stopped-flow analysis of O_2 activation by PolF. 41
Figure S20. Kinetic simulation of the PolF reaction with [U-D_8]Val under the conditions for the Stopped-Flow experiments: 0.3 mM PolF and 1 mM [U-D_8]Val. 42
Figure S21. Stopped-flow analysis of the PolF reaction with deuterated L-Val. 43
Figure S22. Anomalous diffraction electron density difference maps of PolF. 44
Figure S23. Views of the $2F_o$-F_c electron density maps of PolF. 45
Figure S24. Active sites of PolE AlphaFold model and pterin/Fe dependent hydroxylase. 46
Figure S25. LC-MS analysis of the PolE time course assay. 47
Figure S26. NMR characterization of Dns-3,4-dhIle(13C) at 700 MHz in d6-DMSO. 50
Figure S27. NMR characterization of Dns-dh-Ile at 700 MHz in d6-DMSO. 51
Figure S28. LC-MS analysis of PolE assays with QDPR. 52
Figure S29. LC-MS analysis of PolE assays with different PolE mutants. 53
Figure S30. LC-MS analysis of PolE assays with proteogenic amino acids. 54
Figure S31. Time course analysis of PolE/PolF coupled assay. 55
Figure S32. Proposed mechanism of Aziridine formation by TqaL. 56
Figure S33. Sequence logo of DUF6421. 57
References 58
Uncropped gels 59

Supplementary Notes

Note S1. Determination of intrinsic D-KIE by intramolecular competition.

D-KIE for the 3- and 4-H abstraction steps: We first determined the amounts of products formed through 3-H/D abstraction ($P_{3H/D}$: the sum of 3,4-dhVal, 3-OH-Val, and Azi) and the product from 4-H/D abstraction ($P_{4H/D}$: 4-OH-Val) using the amplitudes of the single turnover kinetics data (Fig. 3a-c, Supplementary table 3). Based on the assumption that the ratio of $P_{3H/D}$ and $P_{4H/D}$ is determined by the relative rates of H/D-atom abstraction at positions 3 and 4, we can treat this ratio ($P_{3H/D}/P_{4H/D}$) as the ratio of the rate constants, $k_{3H/D}/k_{4H/D}$:

L-Val: $P_{3H}/P_{4H} = k_{3H}/k_{4H} = 3.0$ (1)

[3-D]Val: $P_{3D}/P_{4H} = k_{3D}/k_{4H} = 0.12$ (2)

[4,4′-D$_6$]Val: $P_{3H}/P_{4D} = k_{3H}/k_{4D} = 107$ (3)

[U-D$_8$]Val: $P_{3D}/P_{4D} = k_{3D}/k_{4D} = 4.3$ (4)

Then, the D-KIE for 3-H abstraction (k_{3H}/k_{3D}) can be calculated by (1)/(2) = 25 or (3)/(4) = 25. The very good agreement of these two ratios from two sets of reactions demonstrates the feasibility of this approach. Similarly, the D-KIE for the 4-H abstraction (k_{4H}/k_{4D}) was calculated to be ~36 based on (4)/(2) = 36 and (3)/(1) = 36, which again agreed very well with each other.

D-KIE of 4-H^+ transfer during the transformation of C3• into 3,4-dhVal: Among the products formed from the C3• intermediate (3,4-dhVal, 3-OH-Val, and Azi), 3,4-dhVal is the only product that requires the 4-H^+ transfer. Consequently, we calculated the ratio of 3,4-dhVal and 3-OH-Val + Azi (P_{dh}/P_{3OA}) in the reactions with L-Val and [4,4′-D$_6$]Val. Then, by dividing P_{dh}/P_{3OA} of L-Val by that of [4,4′-D$_6$]Val, we determined the KIE of 4-H^+ transfer as 1.3.

Note S2. Determination of the rate constants of the H-abstraction steps.

Based on the observed KIEs, we determined the rate constants for the H-atom abstraction steps. Since the [U-D_8]Val reaction showed a D-KIE in both μ-peroxo-Fe(III)$_2$ decay and product formation, D abstraction is the rate-determining step in this reaction. Thus, we used the rate constant of the μ-peroxo-Fe(III)$_2$ decay in the [U-D_8]Val reaction and the proportion of the products formed from 3- and 4-D abstraction (4-OH-Val vs the sum of 3,4-dhVal, 3-OH-Val, and Azi), we determined the rate constants of the 3- and 4-D abstraction as k_{3D} = 0.14 min^{-1}, and k_{4D} = 0.03 min^{-1}, respectively. The rate constants of 3- and 4-H abstraction were determined using the KIE values (25 and 36, respectively) as k_{3H} = 3.5 and k_{4H} = 1.1 min^{-1}.

Note S3. X-ray crystal structure of Zn(II)-bound PolF.

We solved an initial structure of PolF that we intended to be apo and devoid of metal ions, but that was shown to be mismetalled by a mono-Zn(II) center in metal-binding site 1. The metal identity was confirmed by x-ray fluorescence analysis of apo PolF crystals. Anomalous diffraction datasets collected at the Zn x-ray absorption edge also show a clear peak in the difference map at the site 1 metal binding site (Supplementary Fig. 26). Interestingly, while most other HDOs exhibit extensive disorder in their apo or mono-metallated states[1–3], our Zn(II)•PolF complex remains fully helical in the vicinity of the metal binding site (Fig. 4a). However, our stopped-flow absorption experiments show clear evidence of two populations of metal-bound protein upon mixing with O_2 and substrate, suggesting that PolF likely uses a conformationally assisted iron cofactor loading mechanism, like most other HDOs characterized to date[2,4].

Note S4. Comparison with other C-N bond formation reactions.

Radical-mediated C-N bond formation has been investigated in synthetic reactions[5]. However, most of these reactions proceed by addition of a nitrogen-centered radical to an sp^2-hybridized carbon. Recently, a different mechanism of C-N coupling was reported in photoinitiated reactions by an engineered flavoenzyme *Ac*HYAM, in which an intramolecular C-N bond formation step occurs between a benzyl radical and aniline nitrogen[6]. In DFT analysis, the interaction of the aniline nitrogen lone pair and the benzyl radical, assisted by π-π interaction, was proposed to significantly lower the oxidation potential of the radical, allowing the concerted formation of the C-N bond and radical oxidation. While the PolF substrates, L-Ile/L-Val, do not have aromatic rings essential for C-N bond formation by *Ac*HYAM, if the enzyme active site assists the nitrogen lone pair-radical interaction through steric effects, a similar mechanism may be possible. However, the coordination of α-NH_2 to the Fe_2 cluster makes the nitrogen lone pair unavailable for interaction with C4•. On the other hand, the radical coupling mechanism can proceed with α-NH_2 coordinated to the Fe_2 cluster. Thus, while we cannot eliminate the possibility that α-NH_2 dissociates from the Fe_2 cluster as the reaction progresses to catalyze the lone pair-assisted radical oxidation, our current data are more consistent with the radical coupling mechanism. More study is needed to distinguish these possible mechanisms.

Supplementary Methods

Construction of *polE* and *polF* in-frame deletion mutants.

PCR-based homologous recombination and temperature sensitive plasmids were used to create *Streptomyces cacaoi* deletion mutants as previously described[7]. The 5′ flanking regions of *polE* and *polF* were amplified with primers KA192F/KA192R, and KA194F/KA194R (Supplementary Table 1) to generate a 1,324-bp and 1,457-bp DNA fragments, respectively. The 3′ flanking regions of *polE* and *polF* were amplified with primers KA193F/KA193R, and KA195F/KA195R to generate a 1,545-bp, and 1,381-bp DNA fragments, respectively. Then, the PCR products of 5′- and 3′-flanking regions of each gene were ligated with pKC1139 digested with *EcoR*I and *Hind*III using In-Fusion Cloning kit (Takara Bio) to yield pKC1139*ΔpolE*, and pKC1139*ΔpolF*. The resulting plasmids were verified by sequencing using primers KA199F, KA200F, KA201F, KA202F, KA213, KA47F, and KA47R for *ΔpolE*, KA203F, KA204F, KA205F, KA214F, KA215F, KA47F, and KA47R for *ΔpolF*. The plasmids were then introduced into *E. coli* ET12567/pUZ8002[8,9] by a $CaCl_2$ transformation and was grown at 30 °C for 2 d. The conjugation between *S. cacaoi* WT with *E. coli* ET12567/pUZ8002 carrying pKC1139*ΔpolE*/*F* was carried out as described in the general *Streptomyces* genetics protocol[10]. Then 0.5 mL of overnight cultures of *E. coli* ET12567/pUZ8002/pKC1139 *ΔpolE, and ΔpolF* were transferred to 50 mL of fresh LB containing 25 µg/mL of apramycin, 25 µg/ml of kanamycin and 25 µg/ml of chloramphenicol, incubated with shaking at 30 °C, 220 rpm until the OD_{600} reaches 0.4. The cells were collected by centrifugation at 4,000 xg for 5 min at 4 °C, washed twice with 10 mL LB at 4 °C, and resuspended with 5 mL of LB. Meanwhile, 10 µL of *S. cacaoi* spore suspension (10^8–10^9 CFU) was added to a mixture of 250 µL of 2xYT and 250 µL TSB:YEME (a mixed media containing all the ingredients for TSB and yeast extract-malt extract (YEME) media with additions of 5 mM $MgCl_2$ and 0.5% w/v glycine) at 45 °C for 30 min. The spore suspension was then cooled down to room temperature, and was mixed with 500 µL of *E. coli* ET12567/pUZ8002/pKC1139 *ΔpolE*, and *ΔpolF* and let stand at room temperature for 10 min. The mixture was spun down at 6,000 xg for 3 min. The resulting pellet was resuspended with 200–250 µL of the supernatant and plated on MS agar and incubated at 28 °C for 20-21 h. The conjugation plates were then overlaid with 25 µg/mL of apramycin and 25 µg/ml of nalidixic acid and were incubated at 28 °C for 3–5 d until the ex-conjugants were observed. Then, the plates were transferred to 37 °C for 2 d, and the ex-conjugants were streaked on fresh MS agar containing 25 µg/mL of apramycin and 25 µg/mL of nalidixic acid and incubated at 37 °C for 5 d to promote the loss of the temperature sensitive plasmid. The resulting single crossover ex-conjugants were re-streaked for two rounds on MS agar containing 25 µg/mL of nalidixic acid, incubated at 37 °C for 5 d to initiate the double crossover during the elimination of *E. coli*. Then, single colonies were grown in TSB overnight and spread on the MS agar for 7 d until they were fully sporulated. The spores were then collected, diluted, and plated on the MS agar to select for the double crossover ex-conjugants, which were tested for apramycin sensitivity by patching the colonies, in order, on TSB agar with and without apramycin, respectively, and incubated at 37 °C for 5 d. Mycelia of apramycin sensitive ex-conjugants were scraped and used for *Streptomyces* colony PCR o screen for the deletion genotype using primers

KA192F/KA193R (*ΔpolE*), and KA232F/KA232R (*ΔpolF*) (Supplementary table 1), respectively[11]. The amplified PCR fragments corresponding to the size of *polE* and *polF* deletion genotype were sequenced using primers for *S. cacaoi* colony PCR described above.

Cloning of PolF.

PolF was expressed as an N-terminal His-tagged protein. To this end, the *polF* gene was PCR amplified under standard conditions in the protocol for Q5 High-Fidelity DNA polymerase (NEB) from the gDNA of *S. cacaoi* using 28b-polF-NdeI-F and 28b-polF-NdeI-R primers (Supplementary Table 1). The PCR products were digested with NdeI and HindIII and were cloned into the NdeI and HindIII sites of pET-28b using T4 DNA ligase to yield pET-28b-PolF (Supplementary Table 2).

Expression and purification of PolF and PolE (Mutants).

PolF was expressed in *E. coli* BL21(DE3) harboring pET-28b-PolF plasmid. A single colony was grown in LB medium (8 mL) with 50 mg/L kanamycin and incubated at 37 °C, 200 rpm overnight. The entire overnight culture was then used to inoculate 1.5 L of LB medium containing the same antibiotics, which was grown at 37 °C, 220 rpm. When OD_{600} reached 0.6 – 0.8, protein expression was induced with 0.1 mM IPTG. Then, the culture was cooled to 15 °C and incubated at 220 rpm for 20 h. The cells were harvested by centrifugation, washed with buffer A (50 mM Tris pH 7.6, 150 mM NaCl, 10% glycerol), frozen with liquid nitrogen, and stored at -20 °C. Typically, 2.5 - 3.0 g of wet cell paste was obtained per liter of culture.

PolE(Mutants) was expressed in *E. coli* BL21(DE3) harboring pET-28b-PolE(mutants) and pGro7 plasmid. A single colony was grown in LB medium (8 mL) with 50 mg/L kanamycin and 25 mg/L chloramphenicol and incubated at 37 °C, 200 rpm overnight. The entire overnight culture was then used to inoculate 1.5 L of LB medium containing the same antibiotics, which was grown at 37 °C, 220 rpm. When OD_{600} reached 0.3 – 0.5, chaperone protein expression was induced with 5 mg/mL L-arabinose. When OD_{600} reached 0.6 – 0.8, protein expression was induced with 0.1 mM IPTG. Then, the culture was cooled to 15 °C and incubated at 220 rpm for 20 h. The cells were harvested by centrifugation, washed with buffer A (50 mM Tris pH 7.6, 150 mM NaCl, 10% glycerol), frozen with liquid nitrogen, and stored at -20 °C. Typically, 2.5 - 3.0 g of wet cell paste was obtained per liter of culture.

For PolF (PolE) purification, the cell pellet of *E. coli* BL21(DE3)/ pET-28b-PolF (*E. coli* BL21(DE3)/ pET-28b-PolE/pGro7) was suspended in five volumes of Buffer A, homogenized by a Dounce homogenizer, and lysed by two passages through a French pressure cell operated at 14,000 psi. The lysate was clarified by centrifugation (21,000 x g, 20 min, 4 °C), and the supernatant was incubated with Ni-NTA agarose resin (Genesee Scientific, 20 mL equilibrated in Buffer A supplemented with 40 mM imidazole) for 1 h at 4 °C. The resin was subsequently packed into a column and was washed with 10 column volumes (CV) of buffer A supplemented with 40 mM imidazole. The bound proteins were eluted with buffer A supplemented with 200 mM imidazole. Fractions containing PolF were confirmed by 12.5% SDS-PAGE and combined. Then, the purified PolF was treated with

10 eq. ethylenediaminetetraacetic acid (EDTA) (pH 8.0) at 4 °C for 1 h. The resulting solution was passed through a Sephadex G-25 column pre-equilibrated with buffer B (50 mM HEPES-NaOH pH 7.6, 150 mM NaCl, 10% glycerol) to remove imidazole and EDTA. The protein concentration was determined by UV absorption at 280 nm. The purified apo-PolF had < 0.1 eq. of Fe per monomer as judged by ferrozine assay[12]. Apo-PolF solution was degassed on a Schlenk line by three sets of 10 cycles of evacuation (~ 10 sec). and refill (~ 5 min, with argon gas). The resulting degassed protein solution was flash-frozen in liquid nitrogen and stored in – 80 °C for further use.

To reconstitute the diiron cofactor, 3 eq. of $Fe^{II}(NH_4)_2(SO_4)_2$ was added dropwise to PolF solution over 10 min in the MBraun glove box at 10 °C. The resulting mixture was incubated at 10 °C for 1 h. The protein was then desalted by a Sephadex G-25 column and stored in buffer B. The resulting holo-PolF had 1.5 ± 0.3 eq. of Fe per PolF monomer as judged by ferrozine assay[12].

Hydrolysis of polyoxin A.

An aliquot (100 µL) of the supernatant of the wt *S. cacaoi* fermentation broth containing ~0.5 mM polyoxin A was mixed with 100 µL of 10 mM NaOH. The reaction mixture was boiled at 100 °C for 2 h. Then, the solution was neutralized by 6 M HCl. The resulting solution was concentrated to 100 µL by lyophilization. An aliquot (30 µL) of the supernatant was derivatized by adding 30 µL acetonitrile, 10 µL of 1 M borate (PH 8.0) and 10 µL of 20 mM DnsCl at 25 °C for 1 h. An aliquot (30 µL) of PolF reaction with L-Ile was also derivatized in the identical manner. The derivatized solutions were centrifuged (14000 xg, 10 min), and 2 µL of each supernatant was analyzed by LC-MS and chromatographed on an Eclipse Plus C18 column (1.8 µm, 2.1 x 50 mm, Agilent) at 50 °C using solvents A (0.3 % formic acid (FA) in water) and B (0.3 % FA in acetonitrile): 0-2 min, 3% B; 2-5 min, 3-25% B linear gradient; 5-25 min, 25-45% B linear gradient; 25-26 min, 45%-90% B linear gradient; 26-28 min, 90% B; 28-28.5 min, 90%-3% B linear gradient; and 28.5-29 min, 3% B. The flow rate was set to 0.5 mL/min. The elution was monitored by UV absorption at 325 nm and ESI-TOF MS. The MS data were analyzed by MassHunter (Agilent Technologies).

Large-scale PolF reaction with L-Ile (L-Val) to purify PA (MAA).

A large scale PolF reaction (80 mL) was performed aerobically with 300 µM L-Ile (L-Val), 1 mM ascorbate, 100 µM $Fe^{II}(NH_4)_2(SO_4)_2$, 30 µM PolF, and 50 mM HEPES-NaOH pH 7.6 for overnight at 25 °C. The reaction mixture was passed through a 10 kDa concentrator (Millipore) to remove the protein. The flow-through was lyophilized to dry. The PA (MAA) containing mixture was dissolved in 10 mL water and 10 mL acetonitrile, derivatized by adding 1. 5 mL of 1M borate (PH 8.0) and 1.5 mL of 20 mM DnsCl at 25 °C for 3 h. The reaction mixture was evaporated to remove all acetonitrile, dissolved in 50 mL water, and neutralized by 0.1 M HCl. Dns-PA (Dns-MAA) was extracted with ethyl acetate (60 mL x 3). The collected organic layer was evaporated. The resulting Dns-PA (Dns-MAA) was dissolved in 1.5 mL acetonitrile and purified by HPLC equipped with an ODS Hypersil

column (3 µm, 4.6 x 150 mm, Thermo Fischer Scientific) at 50 °C using solvents A (water) and B (acetonitrile). Chromatography was performed at a flow rate of 1.5 mL/min with a linear gradient of 0 - 40% solvent B over 25 min. The elution was monitored by UV absorbance at 325 nm. The peak eluted at 14 -16 min was collected. ~ 0.8 mg of Dns-PA (1 mg of Dns-MAA) was obtained for NMR analysis.

[4,4'-D6]Val synthesis.

This compound was synthesized by following the reported patent, as shown in the scheme below[13]. Compound 1 (Com1, 500 mg) was dissolved in 10 mL anhydrous toluene and evaporated to dry. Then, 3.6 mL dry dimethylformamide (DMF) was added under argon. The solution was cooled to 0 °C for 5 min. Subsequently, 50 mg ground NaOH was added to the solution with vigorous stirring for 5 min, followed by a dropwise addition of 125 µL 2-bromopropane-d6 at 0 °C. After stirring for 30 min at 0 °C, the temperature was brought to room temperature and stirred for 2 h. After that, the solution was cooled on ice for 5 min and an acetic acid solution (75 µL of glacial acetic acid in 1 mL of water) was added dropwise. Compound 2 (Com2) precipitated out during the addition of acetic acid. The mixture was filtered, washed with water, then dried. Com 2 was obtained as red powder (540 mg). For the hydrolysis, 540 mg Com2 was dissolved in a mixture of 3.4 mL methanol, and then was added 4.8 mL of 6 M HCl and 1.2 mL water. The mixture was boiled under reflux for 30 min. Then the reaction mixture was evaporated, and was added 10 mL water. The precipitate was removed by filtration and washed with 5 mL water 3 times. The collected aqueous phase was neutralized by 25% ammonium hydroxide solution. The remaining chiral ligand in the solution was extracted by methylene chloride (3 x 30 mL). To purify the resulting [4,4'-D_6]Val, the aqueous layer was applied to a column packed with Dowex 50Wx8 100-200 H Fine Mesh Ion Exchange Resin (Amberchrom, H^+ form, 30 mL). Then, the resin was first washed by 150 mL water, followed by 60 mL of 5% ammonium hydroxide solution. [4,4'-D_6]Val was eluted after 30 mL of 5% ammonium hydroxide solution. The fractions containing [4,4'-D_6]Val were lyophilized to yield white powder. The sample was derivatized by DnsCl and quantified using LC-MS by adding 1 mM L-Val as an internal standard. This procedure yielded 52.9 mg of [4,4'-D_6]Val with overall 43 % yield from Com1. LC-HRMS (ESI-TOF) m/z $[M+H]^+$ calculated for $C_{17}H_{17}D_6N_2O_4S^+$: 357.1750; found: 357.1758.

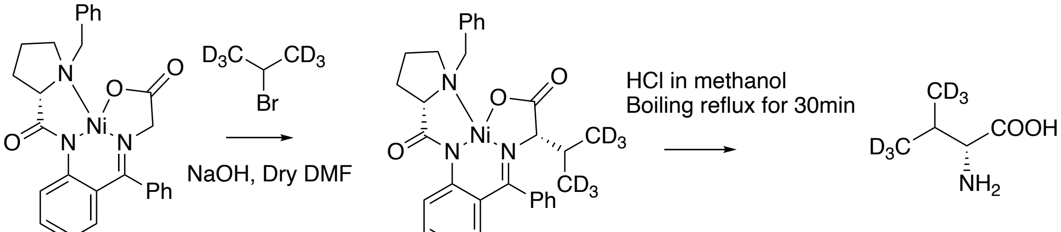

Com1

Com2

[4,4'-D_6]-Val

Large scale PolE reaction with L-Ile to purify dh-Ile (^{13}C-dh-Ile).

A large scale PolE reaction (80 mL, 40 mL for $^{13}C_6$-Ile) was performed aerobically with 150 µM L-Ile, 1 mM ascorbate, 100 µM $Fe^{II}(NH_4)_2(SO_4)_2$, 15 µM PolE, 0.5 uM QDPR, 1 mM NADH and 50 mM HEPES-NaOH pH 7.6 for 2 h at 25 °C. The reaction mixture was passed through a 10 kDa concentrator (Millipore) to remove the protein. The flow-through was lyophilized to dry. The Dh-Ile containing mixture was dissolved in 15 mL water and 15 mL acetonitrile, derivatized by adding 2 mL of 1M borate (PH 8.0) and 2 mL of 20 mM DnsCl at 25 °C for 3 h. The reaction mixture was evaporated to remove all acetonitrile, dissolved in 30 mL water, and neutralized by 0.1 M HCl. Dns-dh-Ile was extracted with ethyl acetate (30 mL x 3). The collected organic layer was evaporated. The resulting Dns-Dh-Ile was dissolved in 1.5 mL acetonitrile and purified by HPLC equipped with an HPH C18 column (3 µm, 4.6 x 150 mm, Agilent) at 40 °C using solvents A (0.1 % FA in water) and B (acetonitrile). Chromatography was performed at a flow rate of 1.0 mL/min with a linear gradient of 20 - 95% solvent B over 17 min. The elution was monitored by UV absorbance at 325 nm. The peak eluted at 12-13 min was collected. ~ 0.7 mg of Dns-dh-Ile (0.3 mg of ^{13}C labeled Dns-dh-Ile) was obtained for NMR analysis.

Supplementary tables

Table S1. Oligonucleotide primers used in this study.

Oligomer	Sequence 5′-3′	Description
28b-polF-NdeI-F<br>28b-polF-HindIII-R	aaaaaaCATATGaccgtgaacgctttggccctgtcc<br>aaaaaaAAGCTTtcaggcgtggccgtcgcgcagtgc	Cloning of *polF*
28b-polE-NdeI-F<br>28b-polE-HindIII-R	aaaaaaCATATGgacgacatgaacccggtgttcgcc<br>aaaaaaAAGCTTtcagacgcccaggcgctccttgac	Cloning of *polE*
PolE-H202A-F<br>PolE-H202A-R	tgcggctcGCGgagtaccaccaccggcagggcga<br>tggtactcCGCgagccgcacccaccacgaggacgc	Site mutation of PolE
PolE-E203A-F<br>PolE-E203A-R	gctccacgCgtaccaccaccggcagggcgaca<br>ggtggtacGcgtggagccgcacccaccacgagg	Site mutation of PolE
PolE-H206A-F<br>PolE-H206A-R	agtaccacGCGcggcagggcgacatgcccgtgc<br>ccctgccgCGCgtggtactcgtggagccgcacc	Site mutation of PolE
PolE-E230A-F<br>PolE-E230A-R	cctcgaagCgctgcgcgtcgacgtctccggcatc<br>cgcgcagcGcttcgaggcctgccagcggcttgc	Site mutation of PolE
KA192F<br>KA192R<br>KA193F<br>KA193R	GATCCGCGGCCGCGCGCGATGCGAGGCGACCTTGATACGAGCCAG<br>TCAGACGCCCAGGCGCTCCTTGCATCAGTTCTCCTGCGGCCGGGTGC<br>CAAGGAGCGCCTGGGCGTCTGACC<br>GACATGATTACGAATTCGATGCCCGTCCCGCATCAGGCCGTTG	*polE* gene knock out and confirmation
KA194F<br>KA194R<br>KA195F<br>KA195R	GATCCGCGGCCGCGCGCGATGGCCGACAAGGCCATCACCGCCATC<br>GTTCCTTTCGCCTGCGGGGGCACACACTCCAAAGGGGACGGGGC<br>CCCCCGCAGGCGAAAGGAACTTCC<br>GACATGATTACGAATTCGATCCACACGACCAGGATGCGCGACCC	*polF* gene knock out
KA199F<br>KA200F<br>KA201F<br>KA202F<br>KA213F	CTCTACACCGTCGTCCCCTC<br>CACGTGGACGGGGACTTC<br>GACCACGAGCTGCTGGCC<br>CAAGCCGATCCTGCACCG<br>CTCGTGTTCCGGATCGAC	pKC1139/Δ*polE* sequencing
KA203F<br>KA204F<br>KA205F<br>KA214F<br>KA215F	AGAACCAGCTCGACGGCC<br>GAAGGAGATCGACGCCGC<br>CAAGGCCGAGACCATGCAC<br>CTGACGTACGAGACCCTGC<br>TCGACCTGGAAGGCGCAG	pKC1139/Δ*polF* sequencing
KA47F<br>KA47R	GCTGGCACGACAGGTTTCCCG<br>CGCCATTCAGGCTGCGCAACTG	pKC1139 5’ and 3’ ends sequencing
KA232F<br>KA232R	GGTCAACAACCAGCGGAGCAGG CTCGCCGTCGAAGAACCGCAG	Δ*polF* in-frame deletion confirmation

Table S2. Plasmid DNA used in this study.

Plasmids	Relevant genotype/Description	reference
pET-28b-PolF	pET28b containing cloned *polF* coding sequence	This study
pET-28b-PolE	pET28b containing cloned *polE* coding sequence	This study
pET-28b-PolE-H202A	pET28b-PolE with H202 mutant to Ala	This study
pET-28b-PolE-E203A	pET28b-PolE with E203 mutant to Ala	This study
pET-28b-PolE-H206A	pET28b-PolE with H206 mutant to Ala	This study
pET-28b-PolE-E230A	pET28b-PolE with E230 mutant to Ala	This study
pKC1139[14]	*aac(3)IV, E. coli-Streptomyces* shuttle plasmid containing a *Streptomyces* temperature-sensitive origin of replication	
pIJ10257[15]	HygR, *ϕ*BT1 *attP-int* derived integration vector for the conjugal transfer of DNA from *E. coli* to *Streptomyces* spp. containing 330-bp *ermE*p* (*Kpn*I-*Pst*I) with ribosome binding site and multicloning site from pIJ8723 cloned into pMS81 cut with *Kpn*I-*Nsi*I	
pKC1139/Δ*polE*	A derivative of pKC1139 containing left and right flanking regions of *polE* for *polE* markerless in-frame deletion	This study
pKC1139/Δ*polF*	A derivative of pKC1139 containing left and right flanking regions of *polF* for *polF* markerless in-frame deletion	This study

Table S3. Kinetic parameters from non-linear curve fitting of the single-turnover product formation kinetics data.[a]

	Kinetic parameters based on product formation			
Substrates [b]		A_0	A	k_1 (min^{-1})
L-Val	All products (H-atom abstraction)[c]	143 ± 21	136 ± 27	0.55 ± 0.1
	4-OH-Val	33 ± 1	32 ± 3	0.59 ± 0.1
	3-OH-Val	9.0 ± 0.6	8.6 ± 1.0	0.62 ± 0.2
	3,4-dhVal	55 ± 4	54 ± 7	0.59 ± 0.1
	Azi	36 ± 17	35 ± 18	0.72 ± 0.2
L-Ile	All products (H-atom abstraction)[c]	118 ± 13	107 ± 17	0.46 ± 0.1
	4-OH-Ile	32 ± 8	30 ± 7	0.39 ± 0.1
	3-OH-Ile	3.7 ± 0.6	3.5 ± 0.7	0.42 ± 0.1
	3,4-dhIle	68 ± 9	64 ± 10	0.63 ± 0.2
	Azi(Ile)	7.8 ± 3.3	7.6 ± 3.3	0.55 ± 0.2
3,4-dhVal	MAA	147 ± 8	139 ± 9	1.2 ± 0.38
[3-D]Val	All products (H-atom abstraction)[c]	170 ± 12	157 ± 24	0.40 ± 0.01
	4-OH-Val	147 ± 11	140 ± 19	0.44 ± 0.1
	3-OH-Val	3.6 ± 0.4	2.3 ± 0.8	0.74 ± 0.4
	3,4-dhVal	9.4 ± 0.9	8.9 ± 1.4	0.44 ± 0.04
	Azi	5.9 ± 1.7	5.6 ± 2.0	0.52 ± 0.05
[4,4'-D_6]Val	All products (H-atom abstraction)[c]	115 ± 29	99 ± 26	0.43 ± 0.2
	4-OH-Val	0.95 ± 0.04	0.84 ± 0.06	0.69 ± 0.03
	3-OH-Val	13 ± 1	12 ± 1	0.61 ± 0.02
	3,4-dhVal	46 ± 5	42 ± 6	0.62 ± 0.05
	Azi	39 ± 13	36 ± 12	0.93 ± 0.4
[U-D_8]Val	All products (H-atom abstraction)[c]	125 ± 25	122 ± 27	0.17 ± 0.02
	4-OH-Val	23 ± 3	23 ± 3	0.15 ± 0.02
	3-OH-Val	11 ± 2	11 ± 2	0.17 ± 0.03
	3,4-dhVal	48 ± 8	48 ± 8	0.17 ± 0.01
	Azi	40 ± 18	39 ± 19	0.23 ± 0.03

[a] Kinetic parameters were determined by non-linear curve fitting of the equation to individual product formation kinetic data in Fig. 1 and 3.
[b] Substrate concentrations were 1 mM.
[c] The kinetic parameters for the total products are determined by fitting the above equation to the kinetics data of the sum of all the products.

Table S4. Kinetic parameters from non-linear curve fitting of stopped-flow kinetics data. [a]

Substrates [b]	A formation	k_1 (min^{-1}) formation	A' formation	k_1' (min^{-1}) formation	k_2 (min^{-1}) decay
L-Val (1 mM)	0.020	840	0.24	8.6	0.92
L-Val (40mM)	0.18	782	0.20	119	0.86
L-Ile	0.21	360	0.30	18	0.71
[3-D]Val	0.025	1395	0.30	11	0.88
[4,4'-D_6]Val	0.019	1253	0.26	7.5	0.79
[U-D_8]Val (1 mM)	0.028	1093	0.28	8.3	0.30
[U-D_8]Val-(40mM)	0.27	1125	0.21	216	0.24
3,4-dhVal	0.35	588	0.13	16	0.96

[a] Kinetic parameters were determined by non-linear curve fitting of equation 2 in Methods to the individual stopped-flow traces in Fig. 5ef, Supplementary Figures 28, 29 and 31.

[b] Substrate concentrations were 1 mM unless specified.

Table S5. Data collection and refinement statistics for x-ray structures of Zn(II)-PolF.

	Zn(II)-PolF native	Zn(II)-PolF anomalous
**Data collection**		
Space group	$P2_12_12_1$	$P2_1$
Wavelength (Å)	0.92012	0.7790
Cell dimensions		
a, *b*, *c* (A)	86.586, 142.849, 161.13	75.783, 139.547, 82.138
α, β, γ (°)	90.0, 90.0, 90.0	90.0, 108.457, 90.0
Resolution (A)	33.71-2.32 (2.37-2.32)	50.00-4.04 (4.11-4.04)
R_{merge}	0.217 (2.099)	0.186 (0.384)
R_{pim}	0.082 (0.789)	0.056 (0.113)
$I / \sigma I$	7.1 (1.1)	13.9 (8.8)
$CC_{1/2}$	0.996 (0.362)	0.975 (0.982)
Completeness (%)	100 (100)	82.58 (82.54)
Redundancy	7.9 (8.1)	11.3 (11.3)
**Refinement**		
Resolution (A)	33.71-2.32	
No. reflections	87415	
R_{work} / R_{free}	0.2115 / 0.2448	
No. atoms		
Protein	12284	
Ligand/ion	18	
Water	349	
B-factors		
Protein	51.74	
Ligand/ion	70.30	
Water	53.75	
R.m.s. deviations		
Bond lengths (A)	0.004	
Bond angles (°)	0.717	
Molprobity clashscore	4.32 (99th percentile)	
Rotamer outliers (%)	0.25	
Ramachandran favored (%)	99.11	

*Values in parentheses are for highest-resolution shell.

Table S6. Data collection and refinement statistics for x-ray structures of L-Ile•Fe_2(II/II)-PolF.

	L-Ile•Fe_2(II/II)-PolF native	L-Ile•Fe_2(II/II)-PolF anomalous
**Data collection**		
Space group	$P2_1$	$P2_1$
Wavelength (Å)	0.9197	1.7437
Cell dimensions		
a, *b*, *c* (A)	78.59, 143.63, 81.45	77.09, 140.65, 84.48
α, β, γ (°)	89.98, 109.78, 90.03	89.99, 110.98, 90.03
Resolution (A)	34.16-2.06 (2.7-2.06)	79.90-3.43 (3.74-3.43)
R_{merge}	0.229 (1.162)	0.056 (0.109)
R_{pim}	0.096 (0.503)	0.024 (0.064)
$I / \sigma I$	7.6 (1.9)	21.5 (9.6)
$CC_{1/2}$	0.973 (0.670)	0.999 (0.981)
Completeness (%)	91.7 (57.2)	67.7 (15.3)
Redundancy	6.8 (6.4)	6.3 (3.9)
**Refinement**		
Resolution (A)	34.16-2.06	
No. reflections	51276	
R_{work} / R_{free}	0.1959 / 0.2346	
No. atoms		
Protein	12416	
Ligand/ion	35	
Water	140	
B-factors		
Protein	30.30	
Ligand/ion	53.09	
Water	34.48	
R.m.s. deviations		
Bond lengths (A)	0.003	
Bond angles (°)	0.60	
Molprobity clashscore	23.79 (99th percentile)	
Rotamer outliers (%)	0.50	
Ramachandran favored (%)	99.0	

*Values in parentheses are for highest-resolution shell.

Supplementary Figures

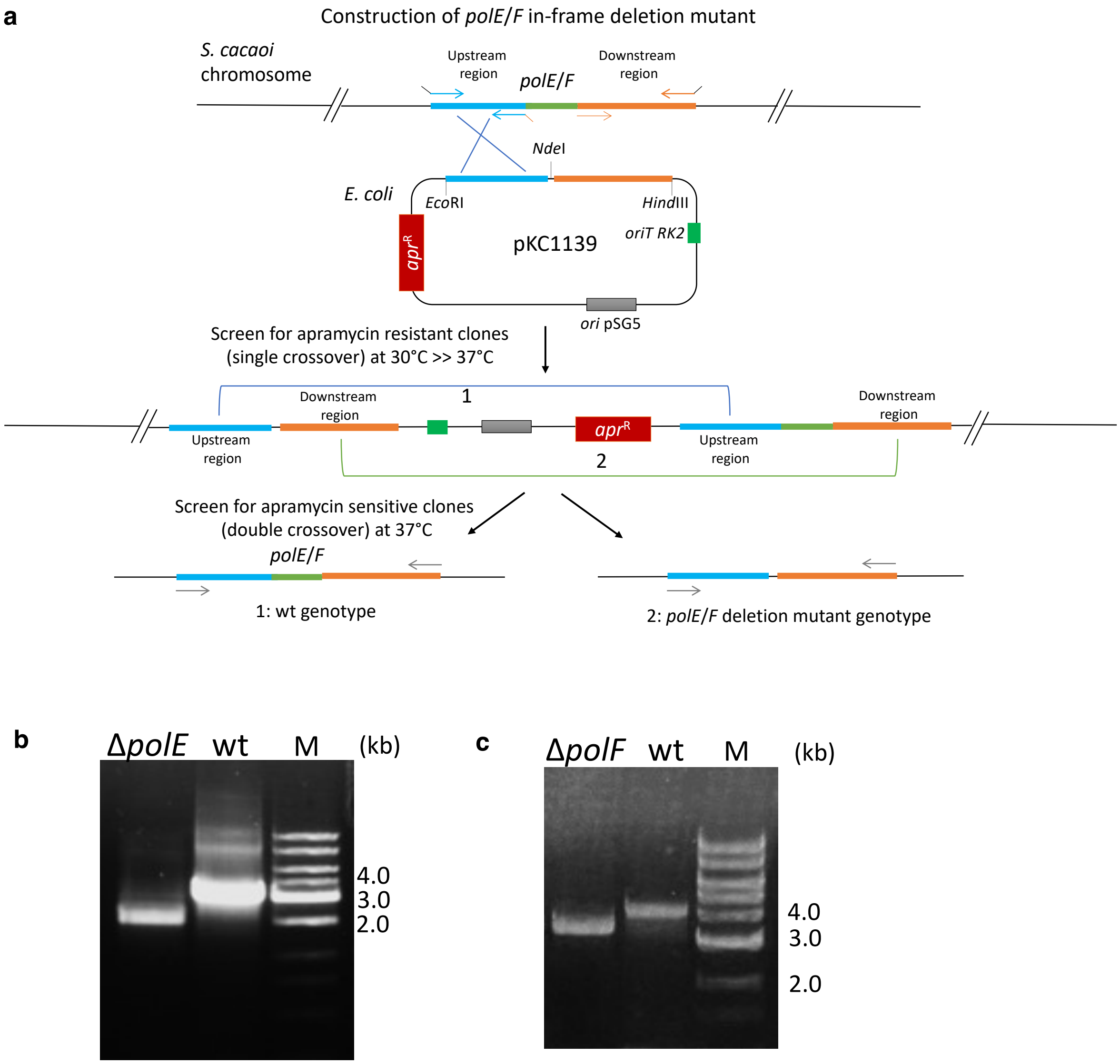

Figure S1. Deletion mutation of the polE, and polF genes. (**a**) A schematic representation of markerless in-frame deletion of *polE* and *polF.* Blue and orange arrows indicate primers used to amplify the upstream 5′ flanking fragments and downstream 3′ flanking fragments of the gene to be deleted, respectively, from the *S. cacaoi* genomic DNA to generate the pKC1139/Δ*polE/F* constructs. Each primer was designed according to the In-Fusion cloning protocol (Takara Bio) to obtain a complementary 5′ overhang of the DNA fragments that the amplicons will be assembled with. The gray arrows indicate a pair of verification primers used to amplify and distinguish the wt and *polE/F* mutant genotypes. The scale bar is not shown as the lengths of the DNA fragments are different, between 1.3 – 1.6 kb, depending on the target *pol* gene. (**b** and **c**). PCR verification of *polE and polF* in-frame deletion. PCR products from *S. cacaoi* Δ*polE* (2,780 bp) and wt (3,958 bp) using primers KA192F/193R (b), and PCR products from *S. cacaoi* Δ*polF* (3,419 bp) and wt (4216 bp) using primers KA232F/R (c). PCR products were subsequently sequenced and confirmed their identities.

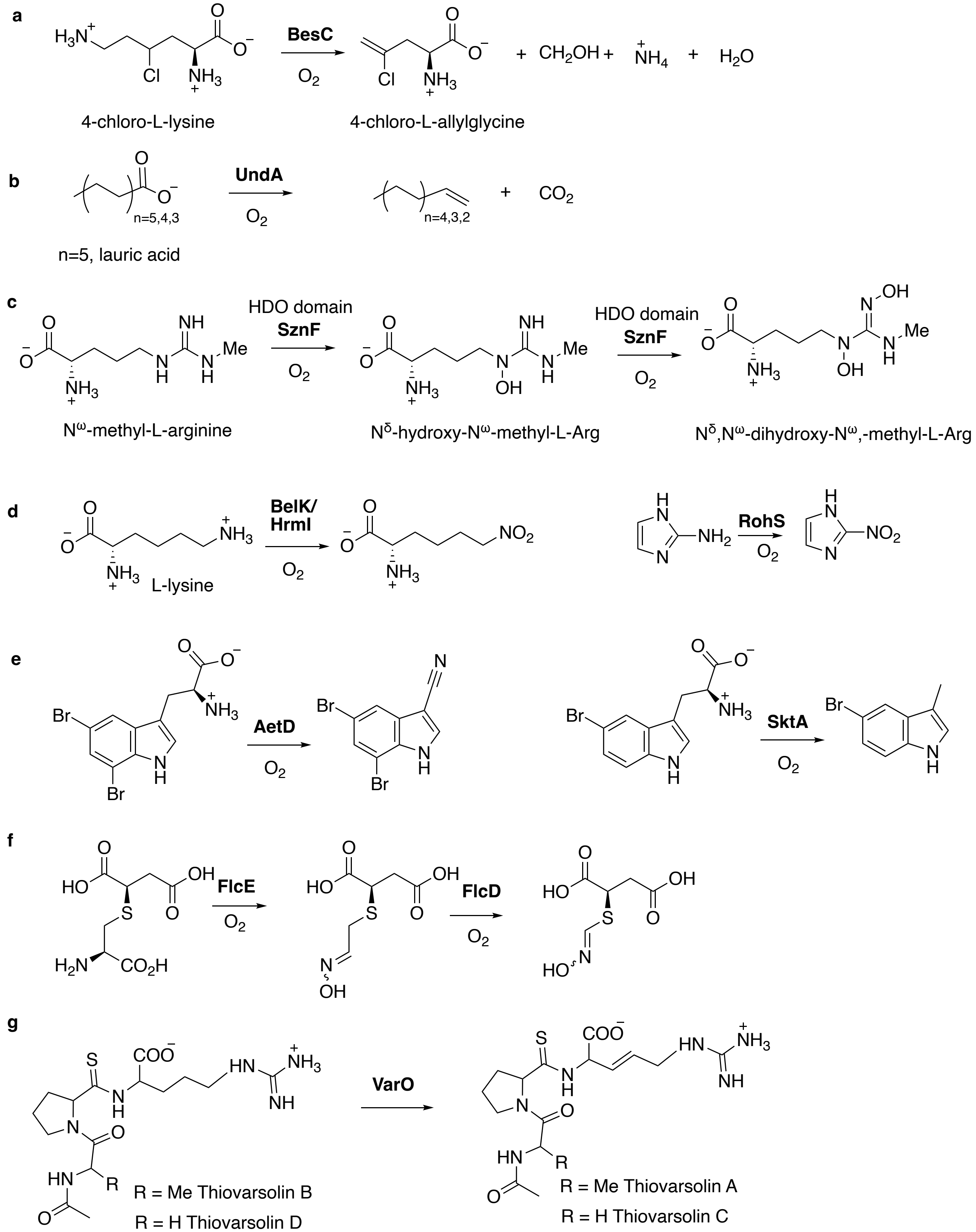

Figure S2. Examples of reactions catalyzed by HDOs.

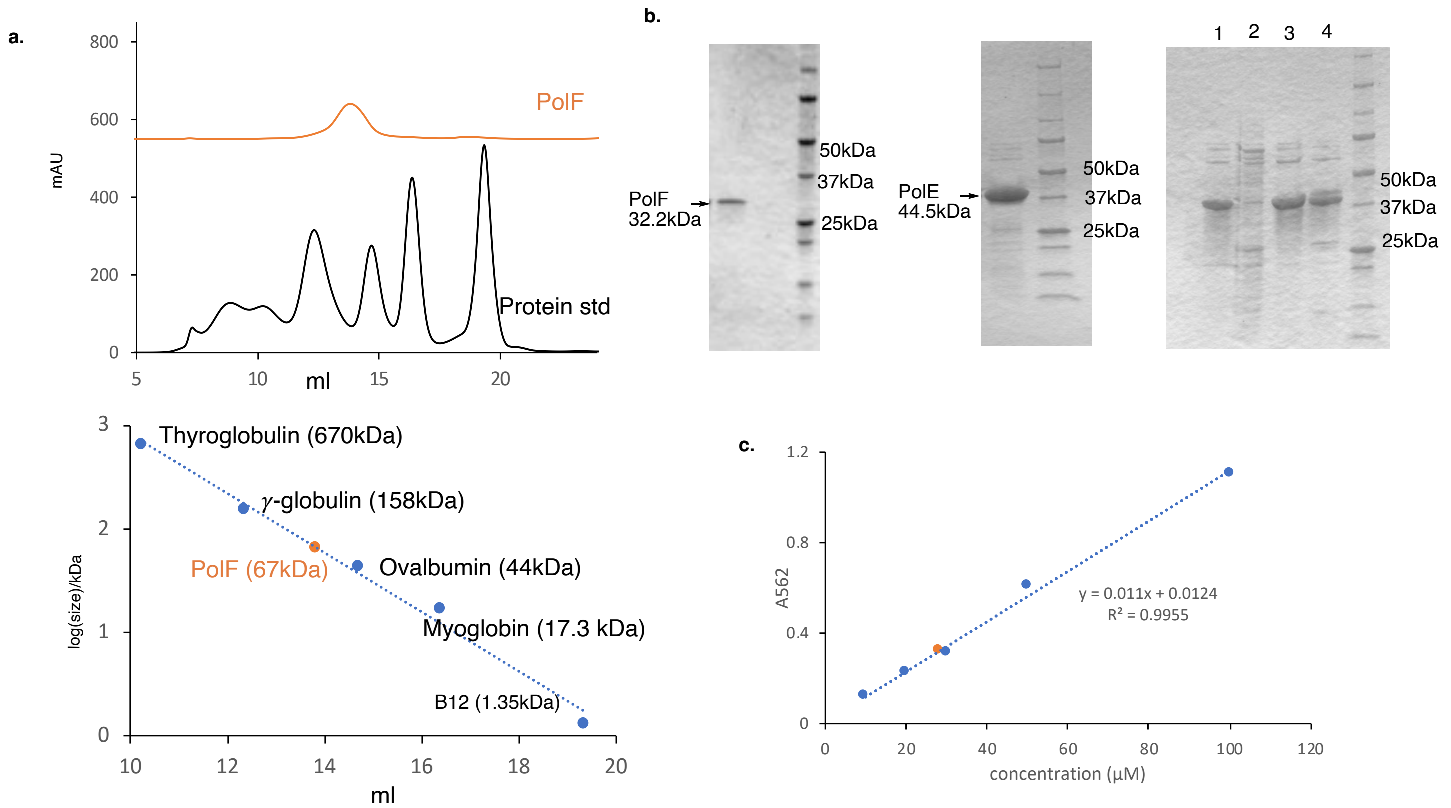

Figure S3. His-tagged PolF and PolE (mutants) purification. **a.** Size-exclusion chromatography of reconstituted PolF. PolF exists as a dimer in solution. **b.** SDS-PAGE (12.5%) of purified 6xHis-tagged PolF (32.2 kDa), PolE (44.5 kDa) and PolE mutants (1. H202A, 2. E203A, 3. H206A, 4. E230A). **c.** Quantitation of Fe(II) in the reconstituted PolF by ferrozine assays. The results showed 28.3 μM Fe in 19.2 μM PolF, suggesting 1.5 Fe per PolF monomer.

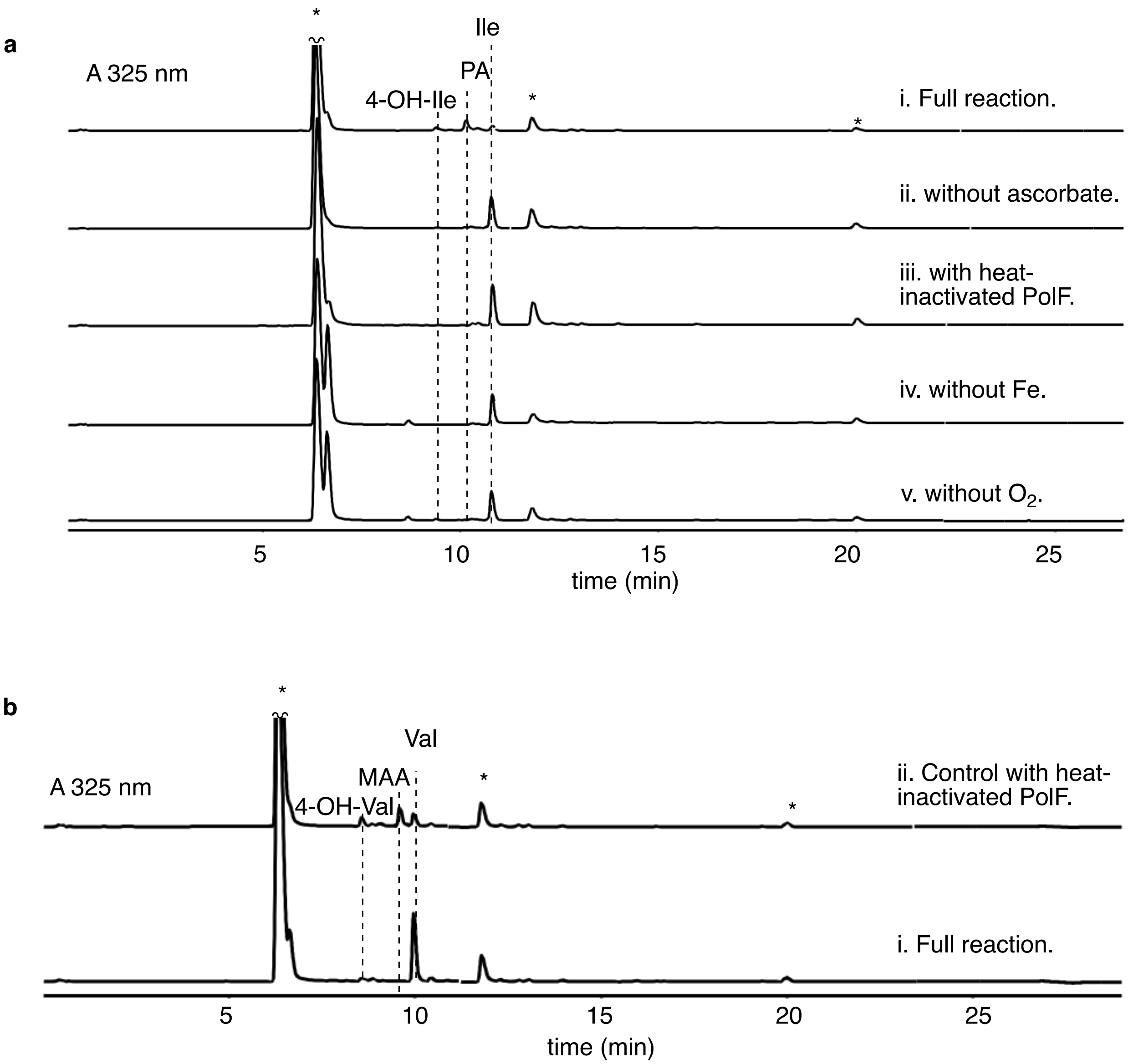

* These peaks are from DnsCl or Dns derivatives unrelated to the PolF activity.

Figure S4. Full retention time range of the chromatograms in Fig. 2b (a) and Fig. 2c (b).

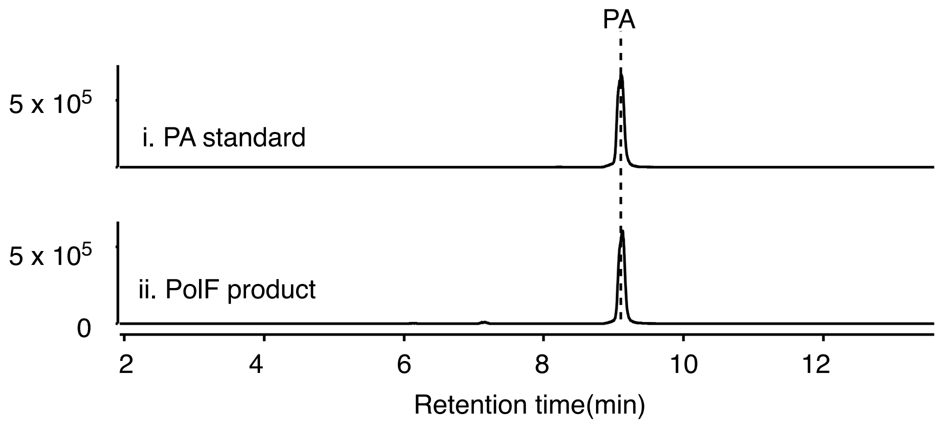

Figure S5. HPLC-MS characterization of PA. (i) PA standard from hydrolyzing crude polyoxin A. (ii) PolF reaction with L-Ile. Shown are the extracted ion chromatography (EIC) of PA at m/z = 361.1217.

a

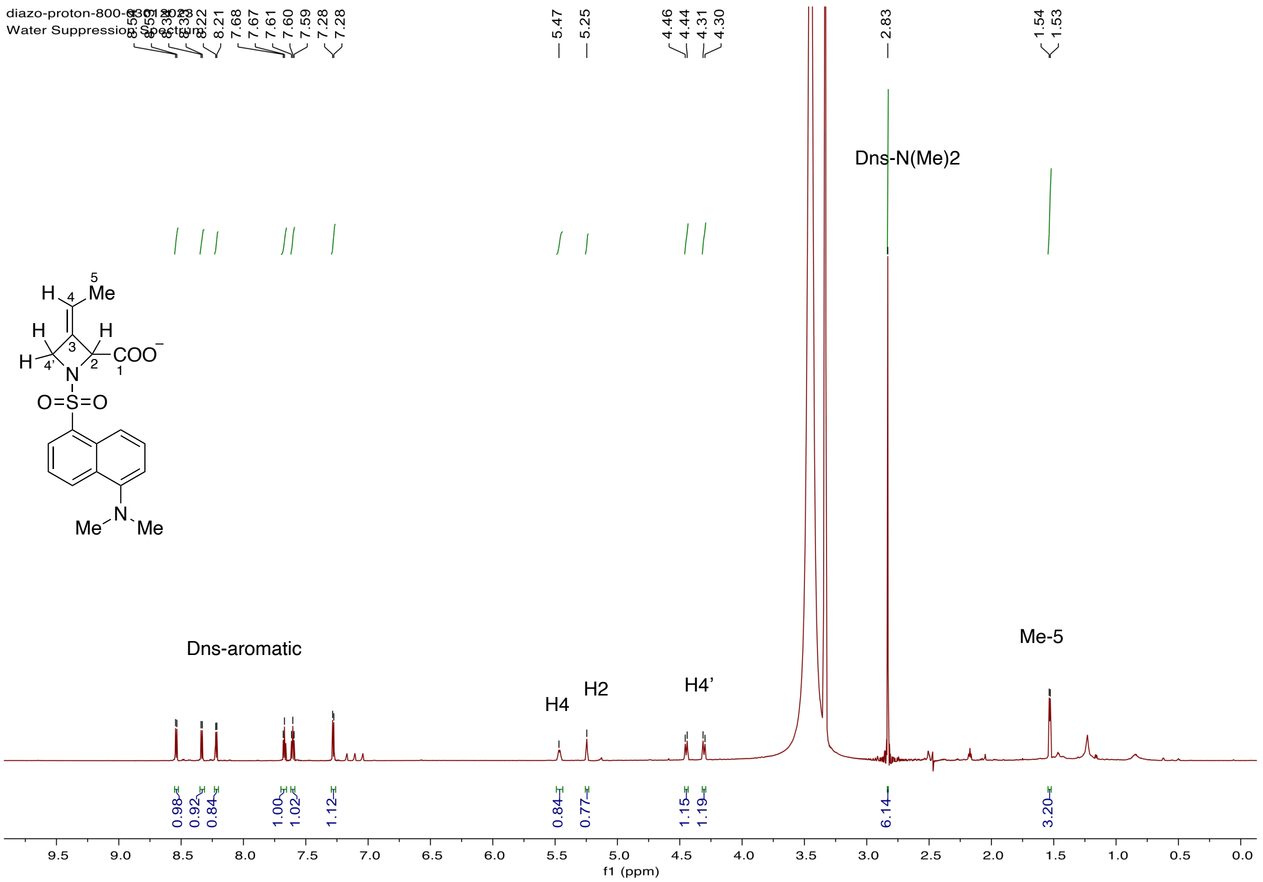
Water Suppression Spectrum
5.47
5.25
4.46
4.44
4.31
4.30
2.83
1.54
1.53
Dns-N(Me)2
Dns-aromatic
H4
H2
H4'
Me-5
0.98
0.92
0.84
1.00
1.02
1.12
0.84
0.77
1.15
1.19
6.14
3.20
f1 (ppm)

b

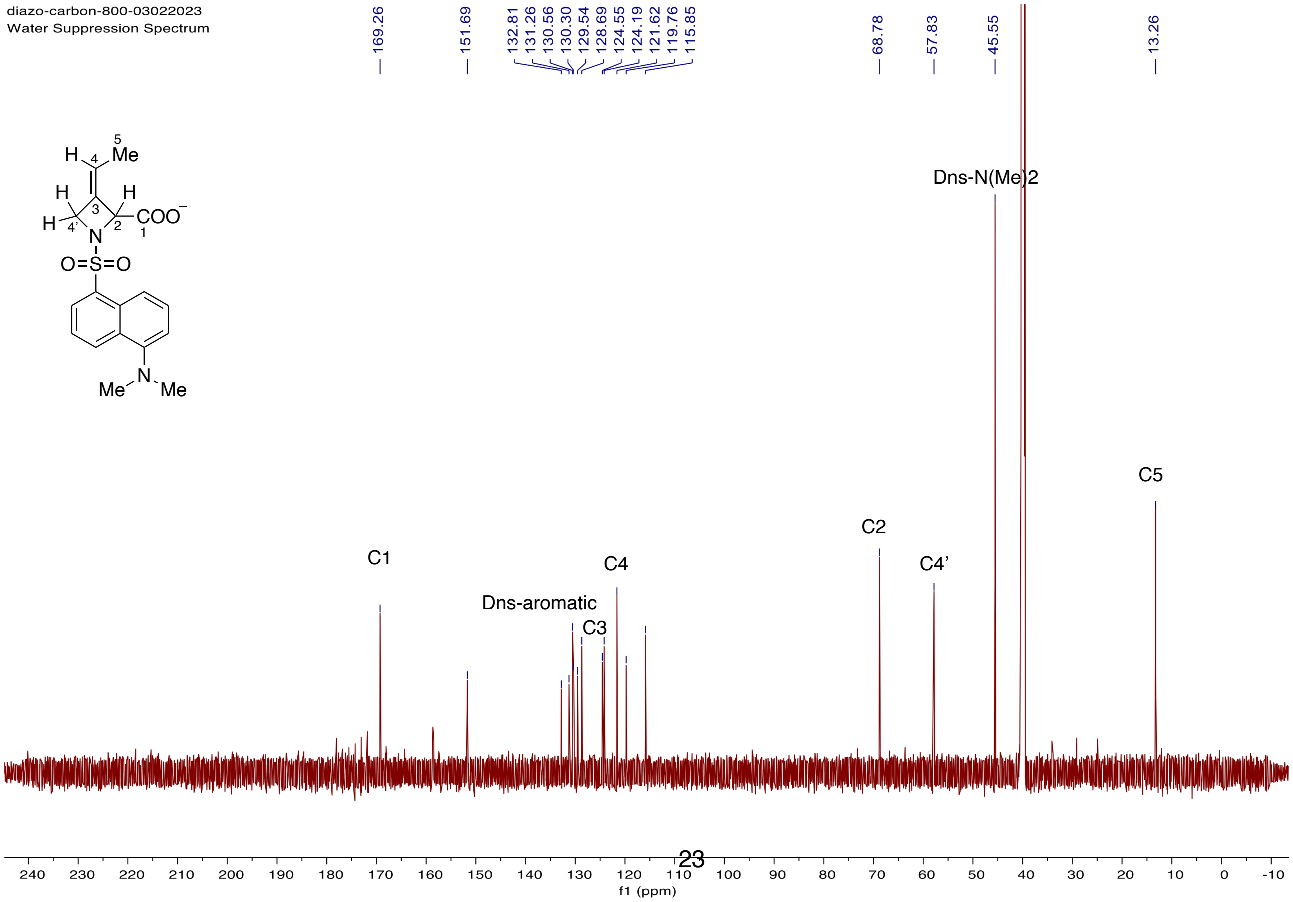
diazo-carbon-800-03022023
Water Suppression Spectrum
169.26
151.69
132.81
131.26
130.56
130.30
129.54
128.69
124.55
124.19
121.62
119.76
115.85
68.78
57.83
45.55
13.26
Dns-N(Me)2
C1
Dns-aromatic
C3
C4
C2
C4'
C5
f1 (ppm)

c

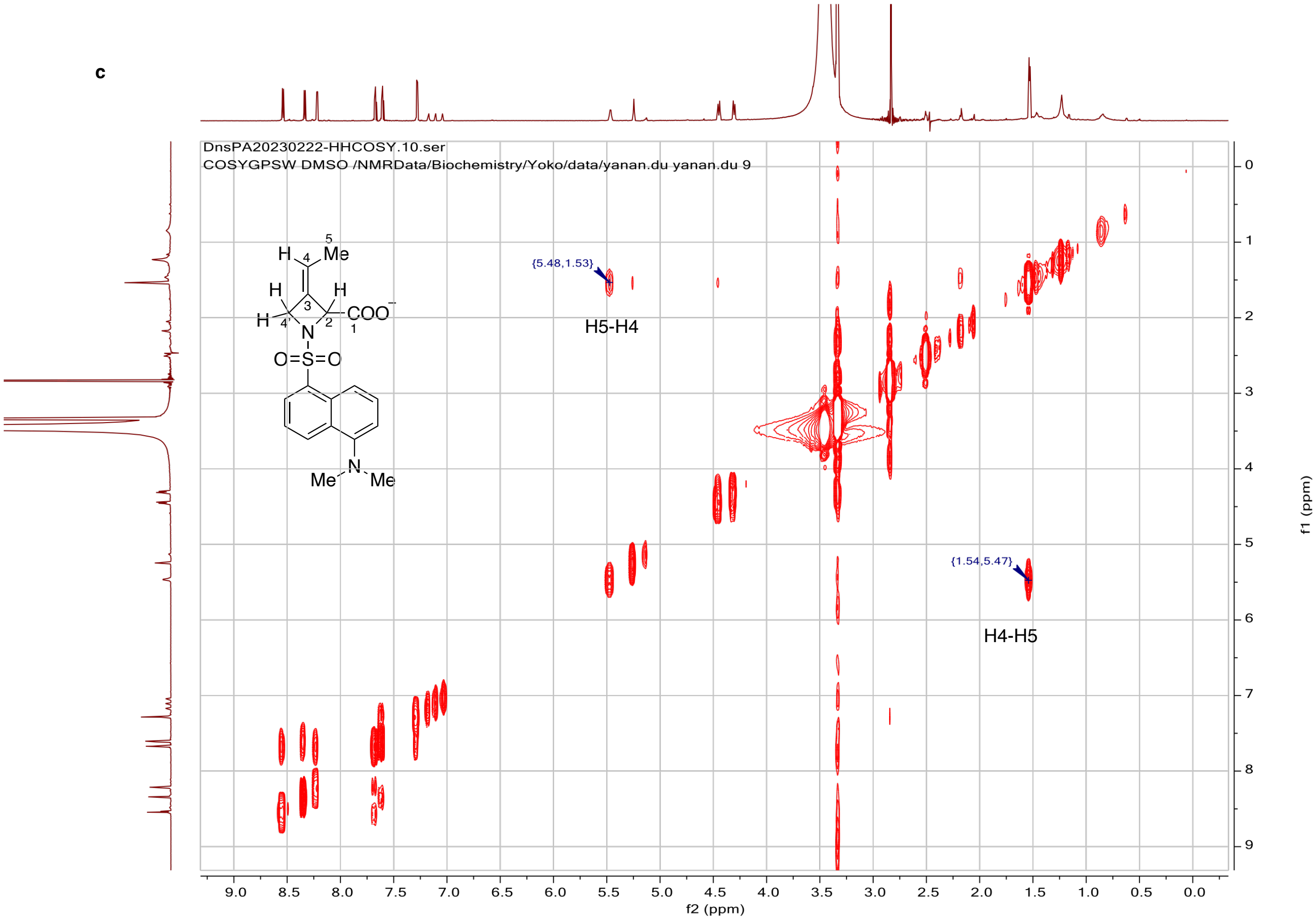
DnsPA20230222-HHCOSY.10.ser
COSYGPSW DMSO /NMRData/Biochemistry/Yoko/data/yanan.du yanan.du 9
{5.48,1.53}
H5-H4
{1.54,5.47}
H4-H5
f2 (ppm)
f1 (ppm)

d

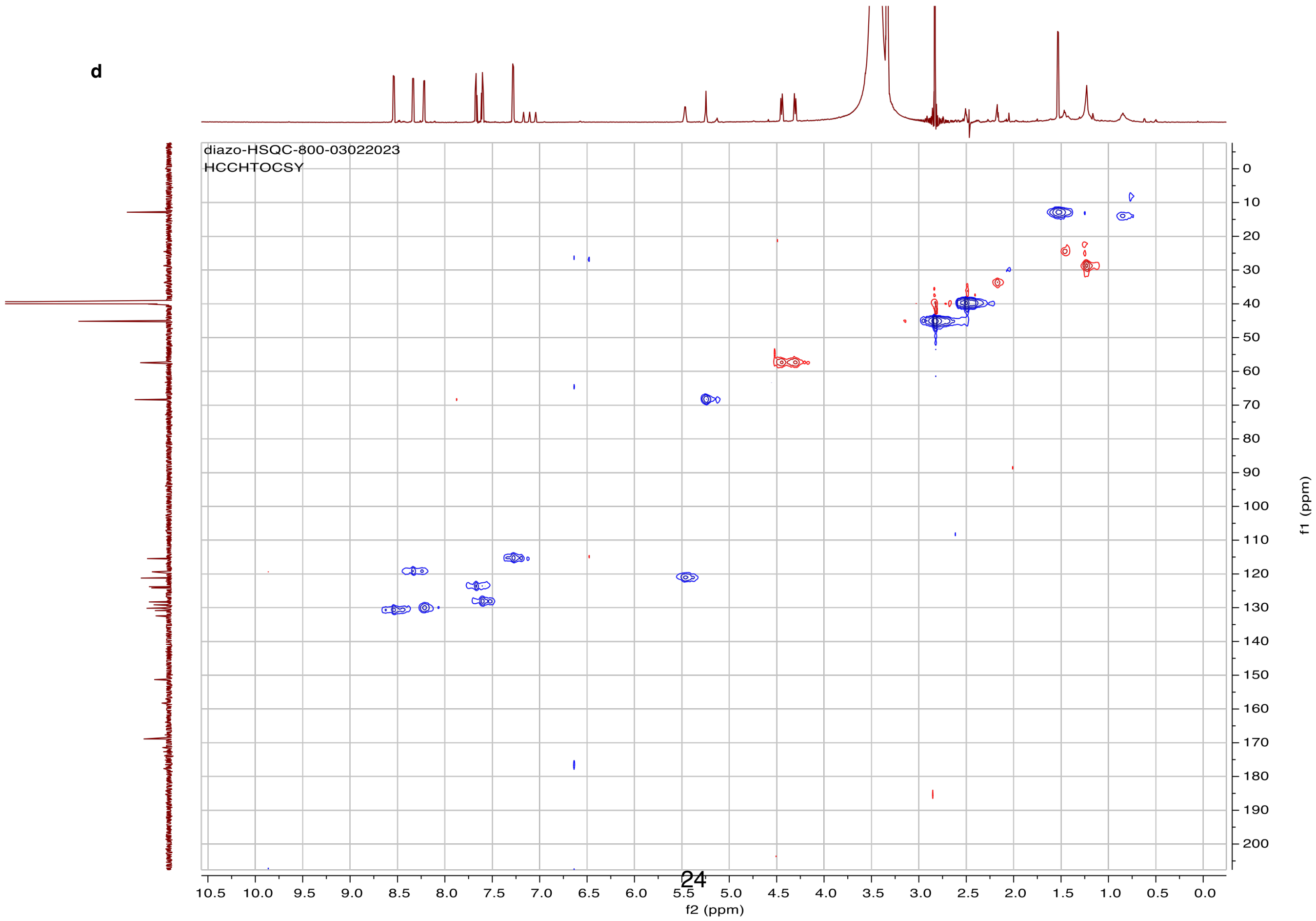
diazo-HSQC-800-03022023
HCCHTOCSY
f2 (ppm)
f1 (ppm)

e

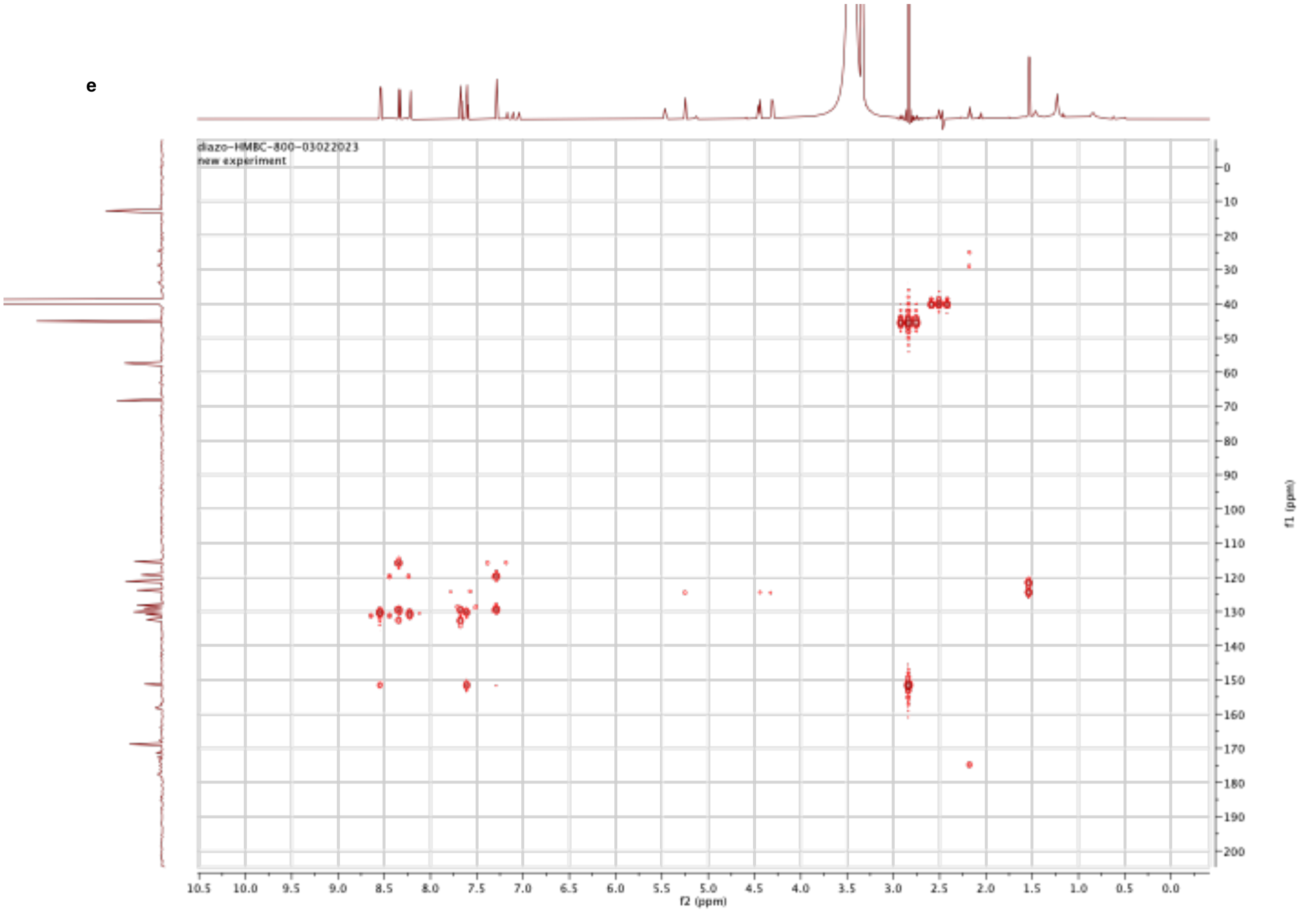

Figure S6. NMR characterization of Dns-PA at 800 MHz in d6-DMSO. **a.** ^{1}H NMR spectrum, **b.** ^{13}C NMR spectrum, **c.** ^{1}H-^{1}H COSY spectrum, **d.** ^{1}H-^{13}C HSQC spectrum, **e.** ^{1}H-^{13}C HMBC spectrum.

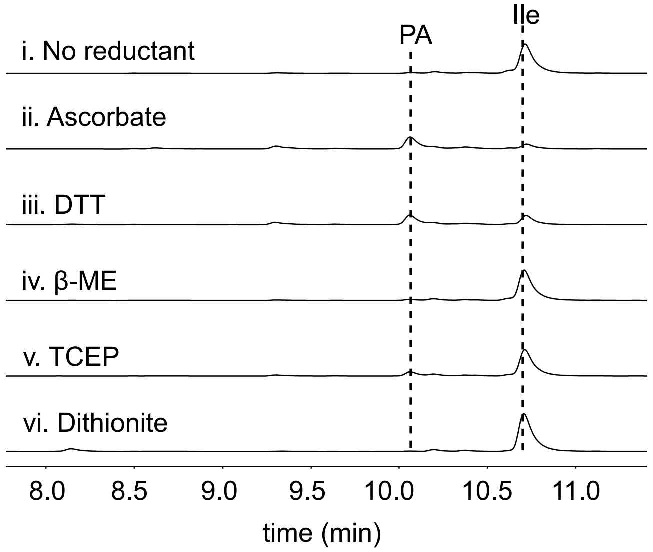

Figure S7. HPLC UV chromatograms (325 nm) of PolF assays with different reductants. The assays were performed with 300 μM L-Ile, 30 μM PolF (apo form), 100 μM Fe(II) and ~0.5 mM O_2, either without any reductant (i), with 1 mM ascorbate (ii), with 1 mM dithiothreitol (DTT) (iii), with 1 mM β-mercaptoethanol (β-ME) (iv), with 1 mM tris(2-carboxyethyl)phosphine (TCEP) (v), and with 1 mM sodium dithionite (vi).

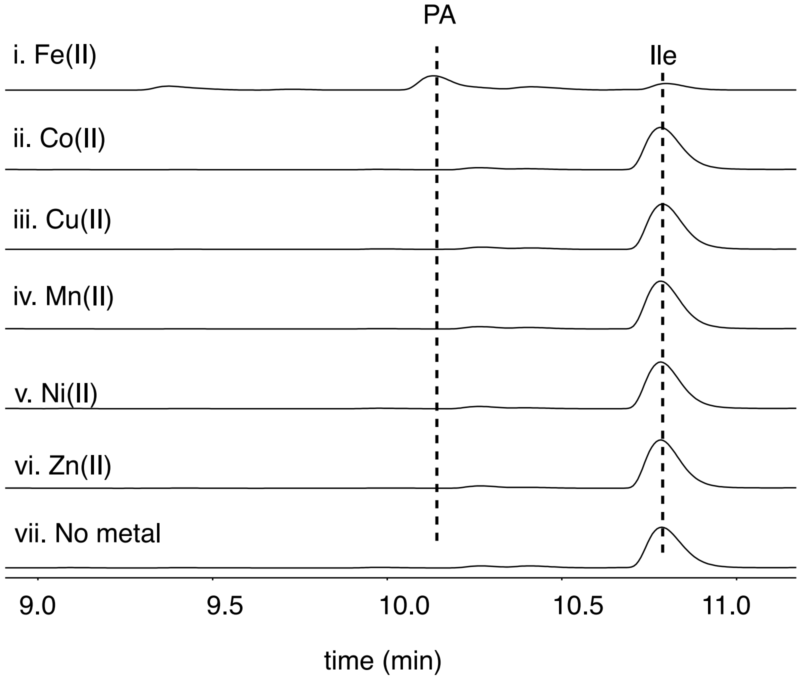

Figure S8. HPLC UV chromatograms (325 nm) of PolF assays with different metal ions. (i) Fe(II), (ii) Co(II), (iii) Cu(II), (iv) Mn(II), (v) Ni (II), and (vi) Zn(II). The assays were performed with 300 μM L-Ile, 30 μM apo-PolF, 100 μM Fe(II) and ~0.5 mM O_2 and 1 mM ascorbate.

a

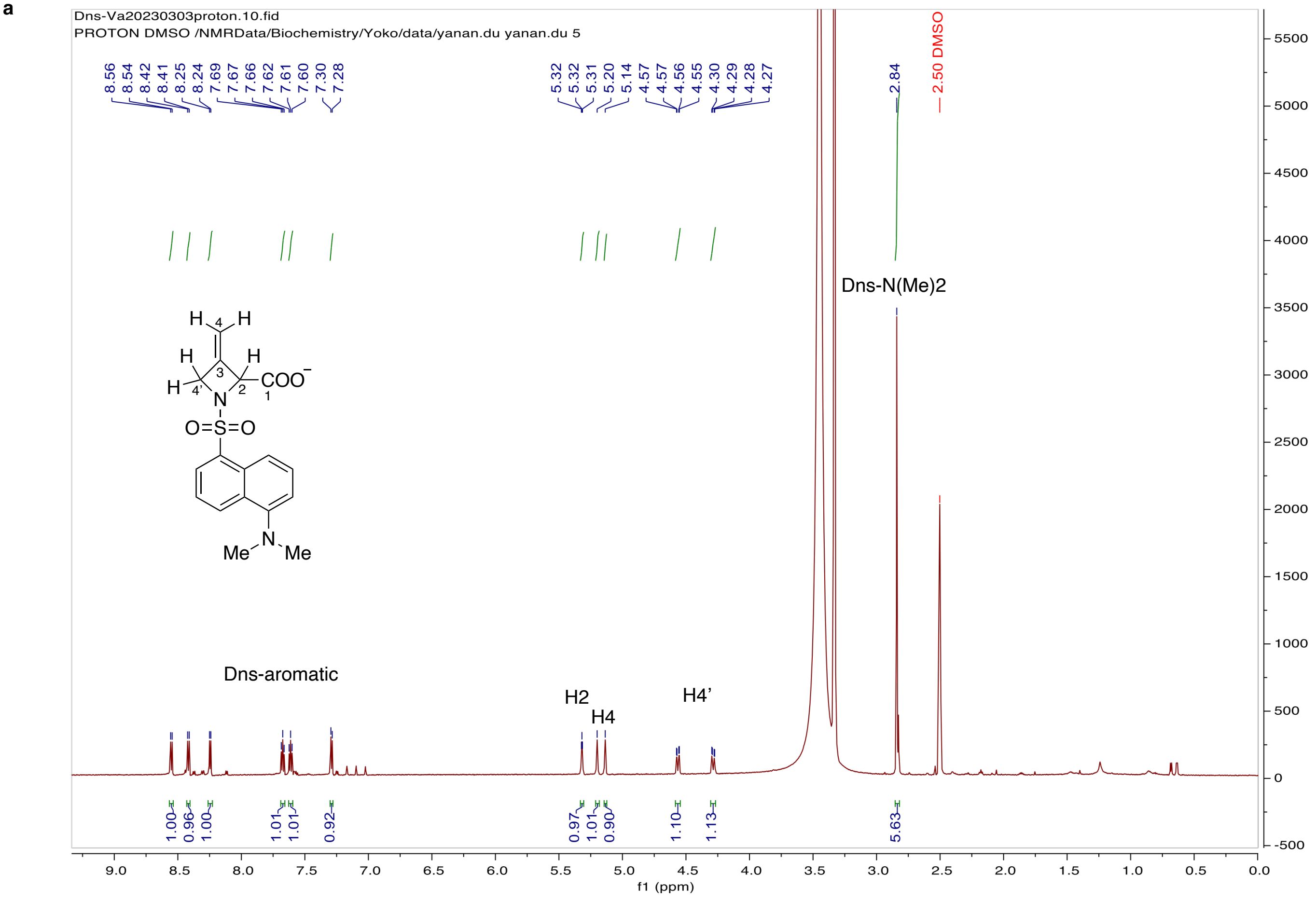
Dns-Va20230303proton.10.fid
PROTON DMSO /NMRData/Biochemistry/Yoko/data/yanan.du yanan.du 5
8.56 8.54 8.42 8.41 8.25 8.24 7.69 7.67 7.66 7.62 7.61 7.60 7.30 7.28
5.32 5.32 5.31 5.20 5.14 4.57 4.57 4.56 4.55 4.30 4.29 4.28 4.27
2.84
2.50 DMSO
Dns-N(Me)2
Dns-aromatic
H2
H4
H4'
1.00 0.96 1.00 1.01 1.01 0.92 0.97 1.01 0.90 1.10 1.13 5.63
f1 (ppm)

b

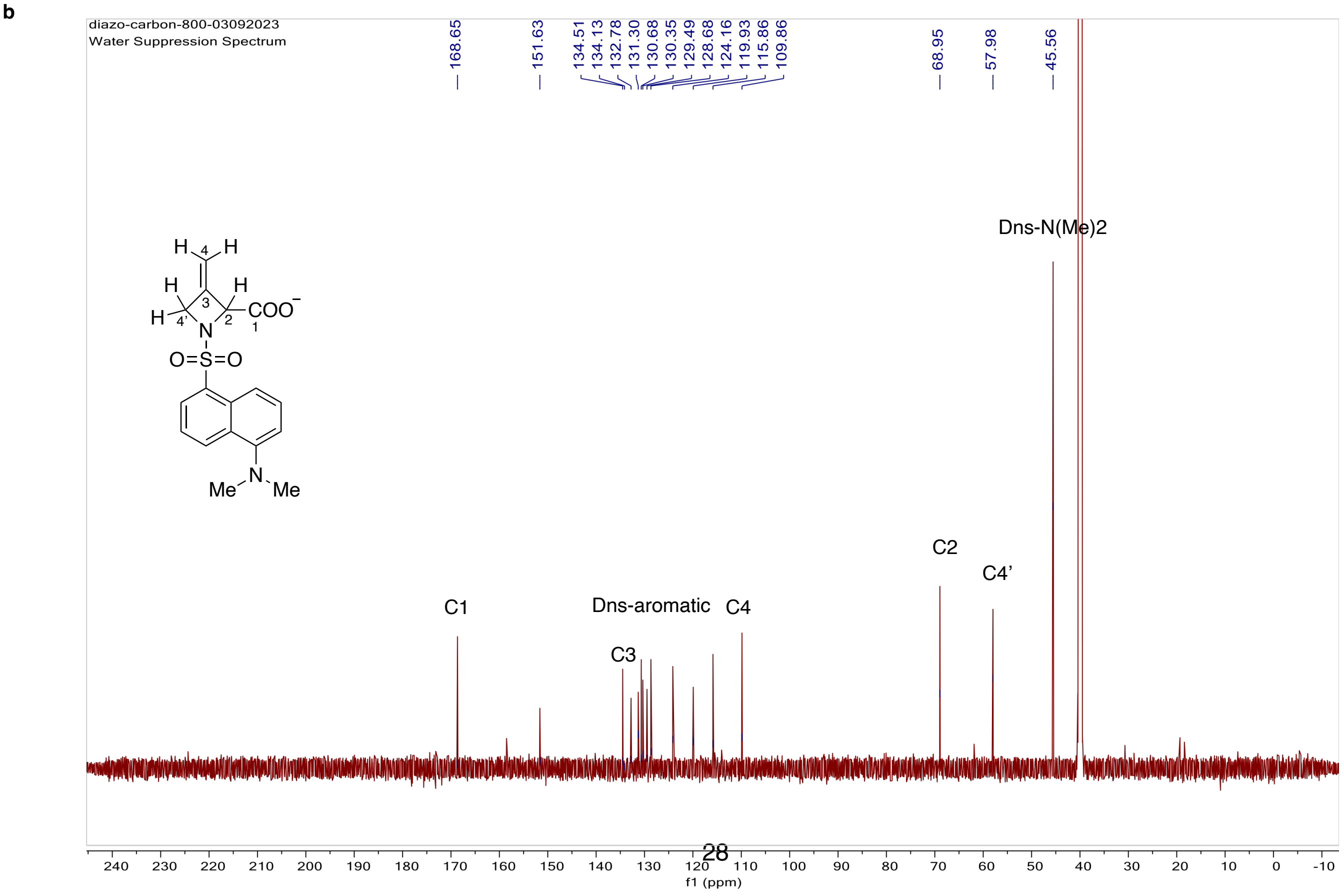
diazo-carbon-800-03092023
Water Suppression Spectrum
168.65
151.63
134.51 134.13 132.78 131.30 130.68 130.35 129.49 128.68 124.16 119.93 115.86 109.86
68.95
57.98
45.56
Dns-N(Me)2
C2
C4'
C1
Dns-aromatic
C4
C3
f1 (ppm)

c

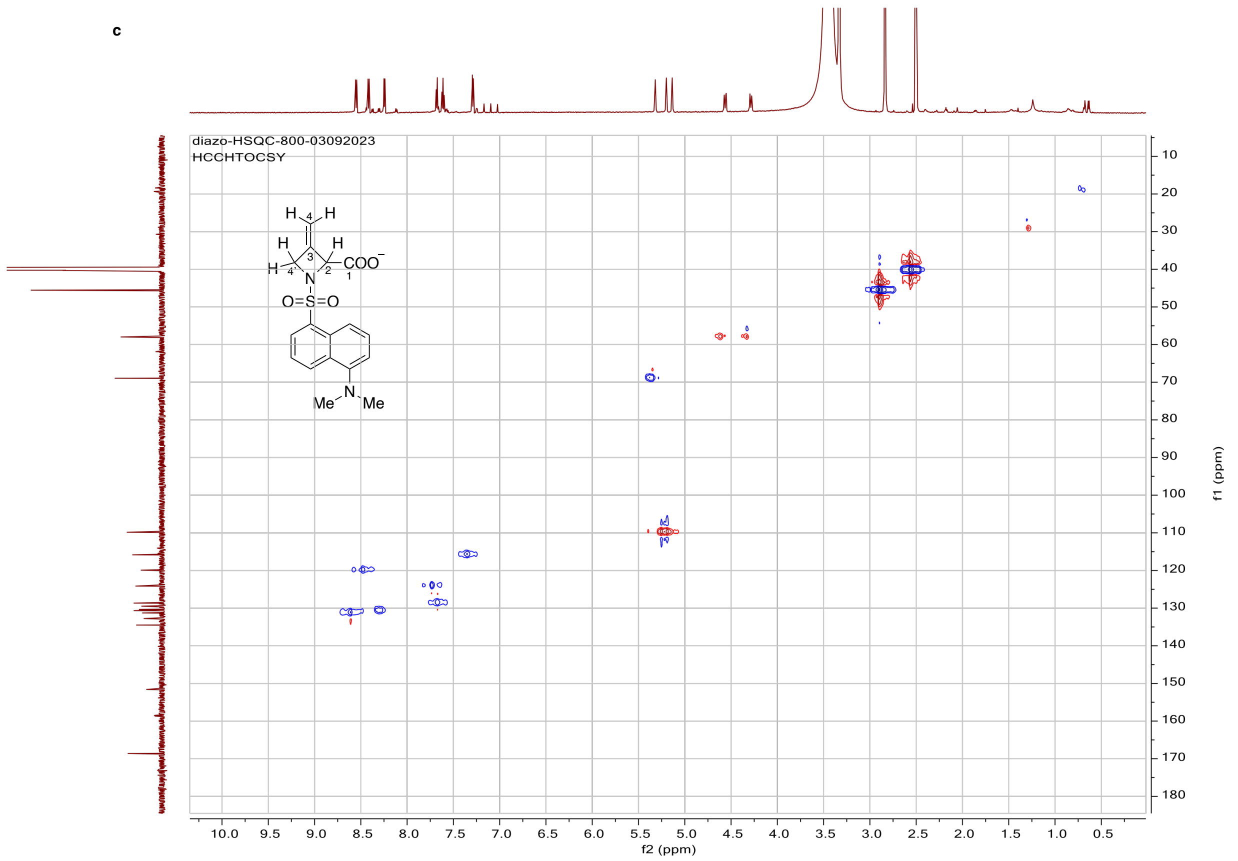

d

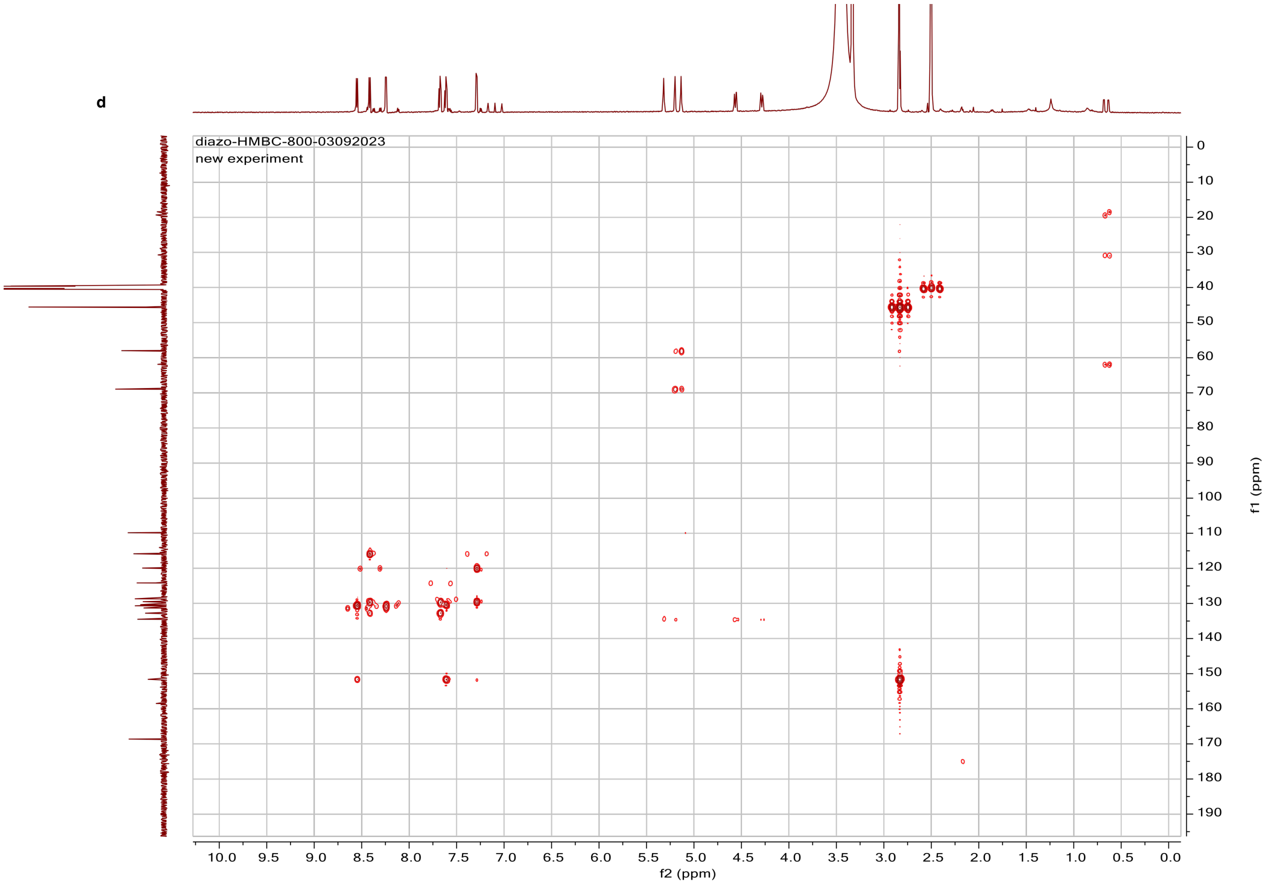

Figure S9. NMR characterization of Dns-MAA at 800 MHz in d6-DMSO. **a.** ^{1}H NMR spectrum, **b.** ^{13}C NMR spectrum, **c.** ^{1}H-^{13}C HSQC spectrum, **d.** ^{1}H-^{13}C HMBC spectrum.

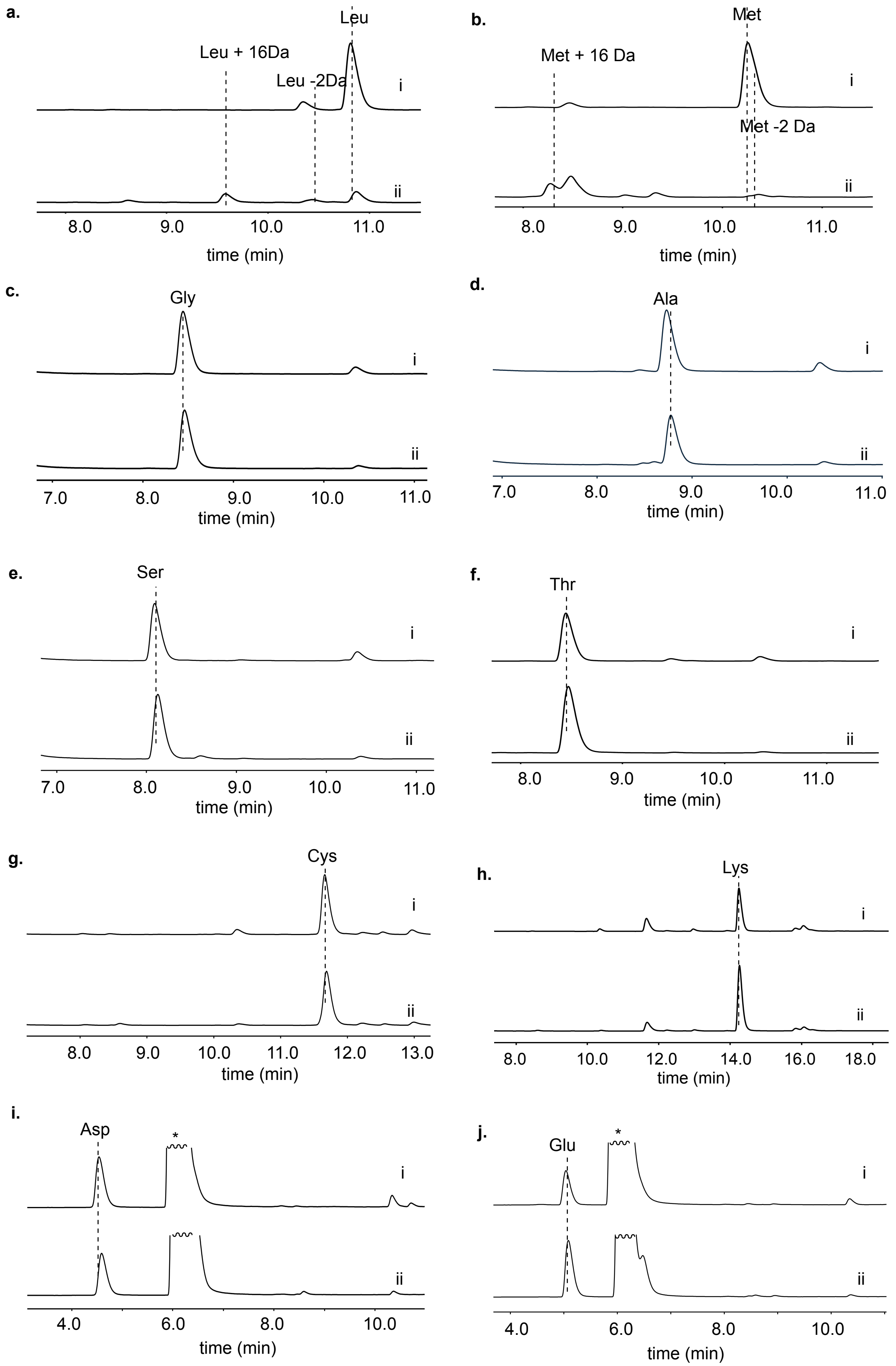

a.
Leu
Leu + 16Da
Leu -2Da
i
ii
8.0
9.0
10.0
11.0
time (min)
b.
Met
Met + 16 Da
Met -2 Da
i
ii
8.0
9.0
10.0
11.0
time (min)
c.
Gly
i
ii
7.0
8.0
9.0
10.0
11.0
time (min)
d.
Ala
i
ii
7.0
8.0
9.0
10.0
11.0
time (min)
e.
Ser
i
ii
7.0
8.0
9.0
10.0
11.0
time (min)
f.
Thr
i
ii
8.0
9.0
10.0
11.0
time (min)
g.
Cys
i
ii
8.0
9.0
10.0
11.0
12.0
13.0
time (min)
h.
Lys
i
ii
8.0
10.0
12.0
14.0
16.0
18.0
time (min)
i.
Asp
*
i
ii
4.0
6.0
8.0
10.0
time (min)
j.
Glu
*
i
ii
4.0
6.0
8.0
10.0
time (min)

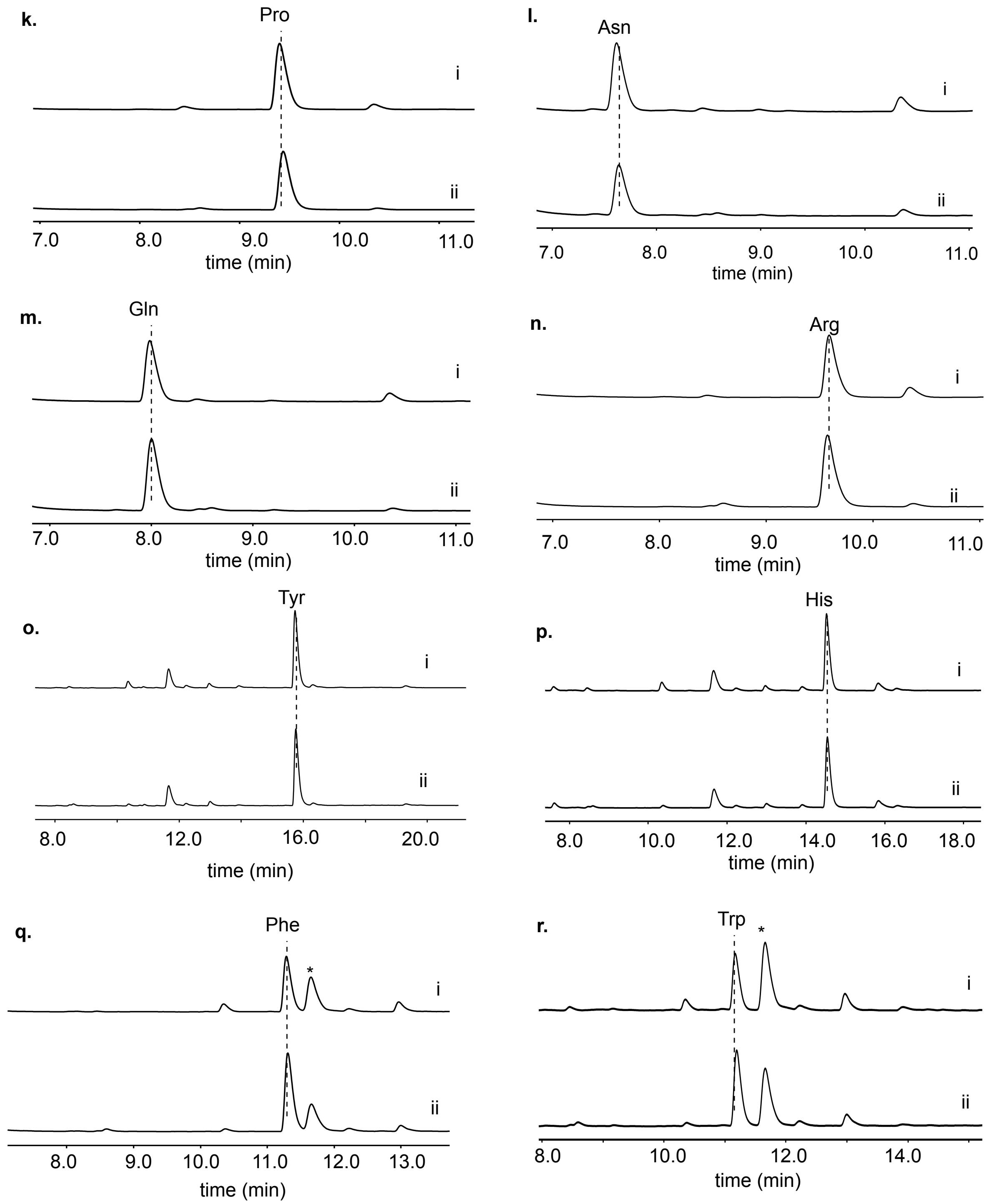

Figure S10. HPLC UV chromatograms (325 nm) of PolF assays with proteogenic amino acids. L -Leu (**a**), L -Met (**b**), L-Gly(**c**), L-Ala(**d**), L-Ser(**e**), L-Thr(**f**), L-Cys(**g**), L-Lys(**h**), L-Asp(**i**), L-Glu(**j**), L-Pro(**k**), L-Asn(**l**), L-Gln(**m**), L-Arg(**n**), L-Tyr(**o**), L-His(**p**), L-Phe(**q**) and L-Trp(**r**). Trace (i) in each panel shows the analysis of an assay with 300 μM L-amino acid, 30 μM apo-PolF, 100 μM Fe(II), and ~0.5 mM O_2 and 1 mM ascorbate. Traces (ii) are controls with boiled PolF. The peaks at 6.5 min are decomposed DnsCl, and the peaks at 11.6 min derives from the PolF sample unrelated to the PolF activity.

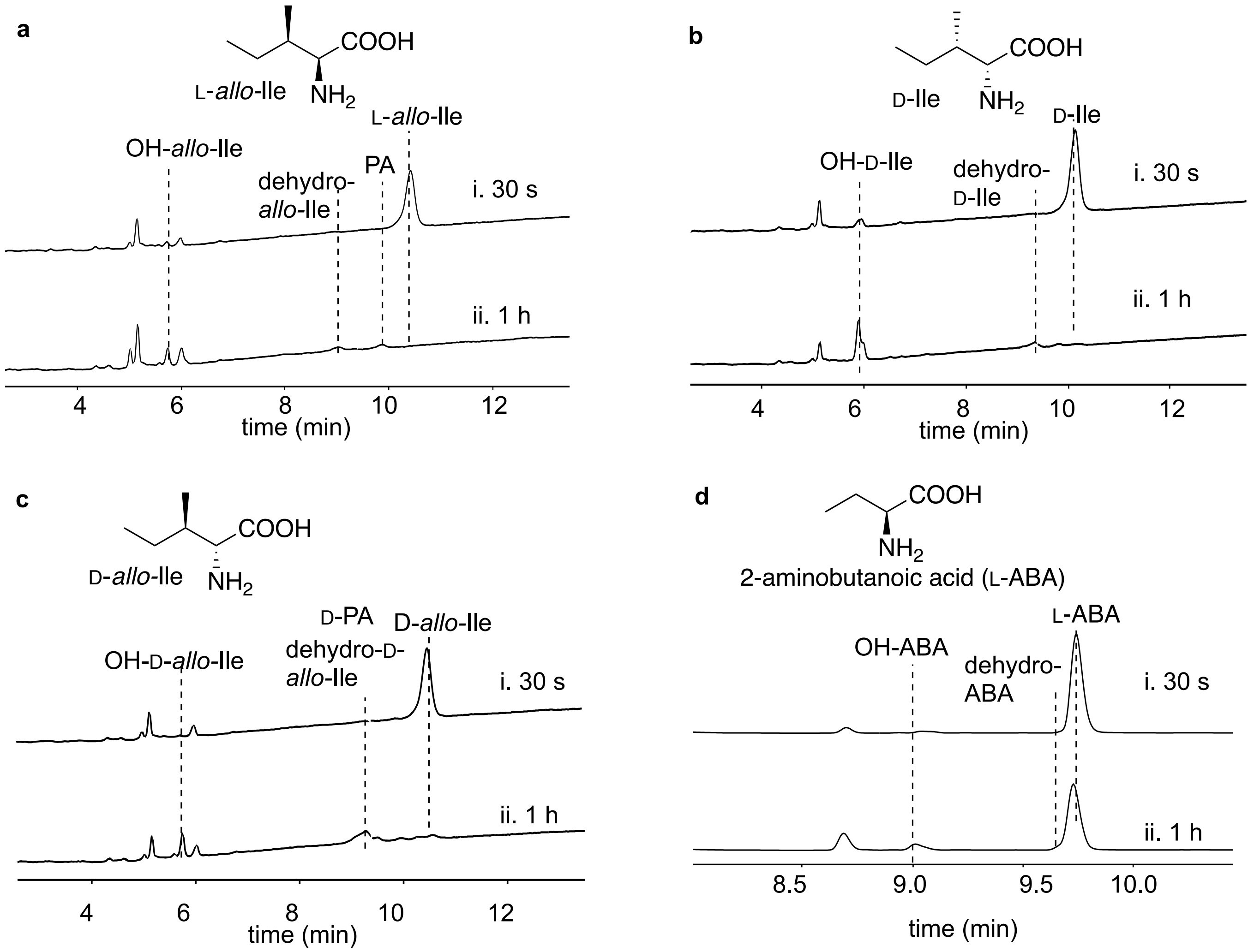

Figure S11. HPLC UV chromatograms (325 nm) of PolF reaction with Ile stereoisomers. L-*allo*-Ile (**a**), D-Ile (**b**), D-*allo*-Ile (**c**), L-ABA (**d**). PolF assays were performed with 300 μM substrate, 30 μM apo-PolF, 100 μM Fe(II), ~0.5 mM O_2, and 1 mM ascorbate. Shown are the chromatograms of the reactions quenched after 30 s (i) and 1 h (ii).

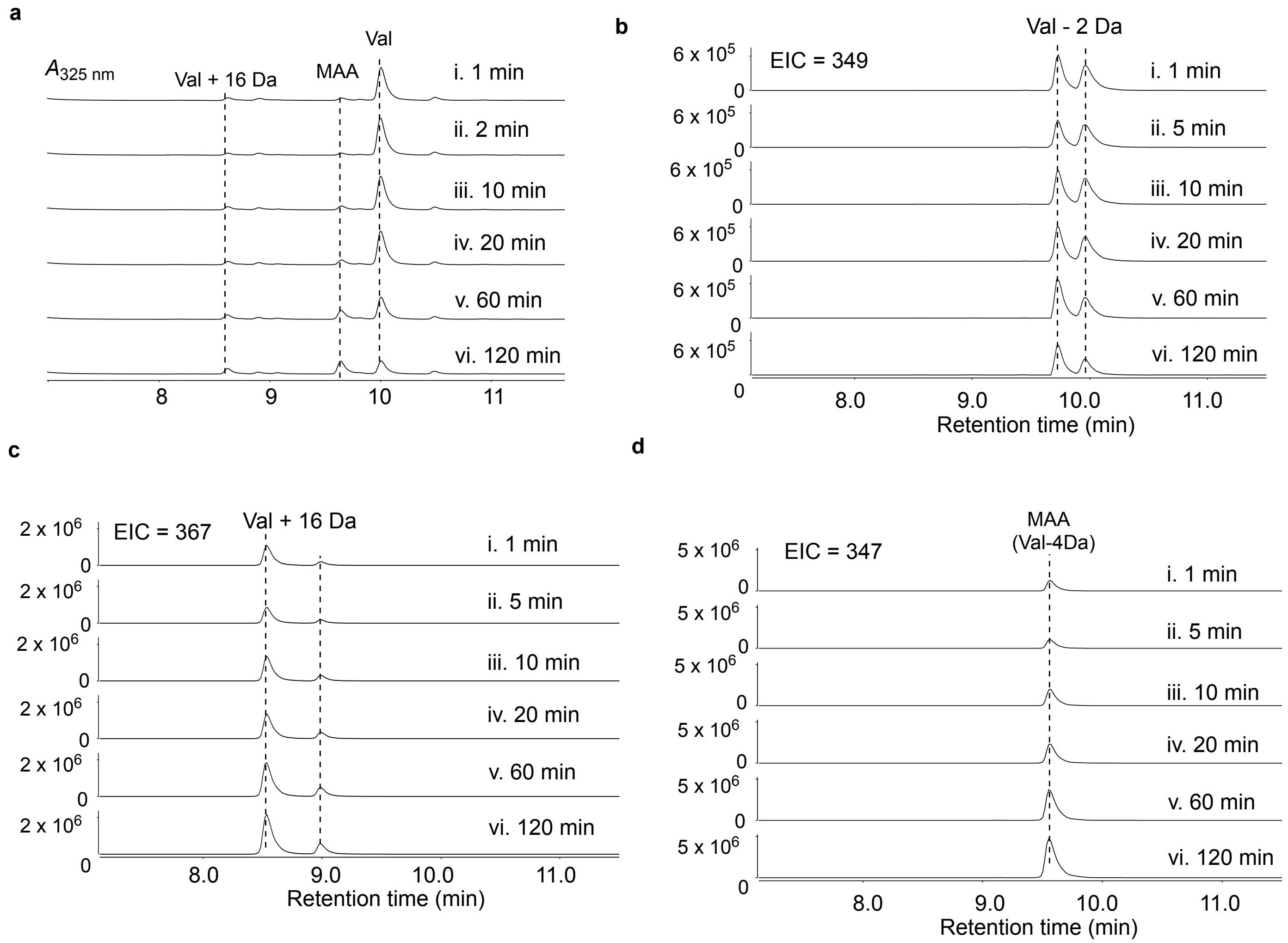

Figure S12. HPLC analysis of a time course of PolF reaction with L-Val under multiple turnover conditions. Shown are chromatograms (**a**) UV chromatograms A = 325 nm, (**b**) EIC = 349.1217, (**c**) EIC = 367.1323, (**d**) EIC = 347.1066. PolF reactions with L-Val quenched at 1 min (i), 5 min (ii), 10 min (iii), 20 min (iv), 60 min (v), and 120 min (vi). PolF reaction was performed with 300 μM L-Val, 30 μM PolF (apo form), 100 μM Fe(II), ~0.5 mM O_2, and 1 mM ascorbate.

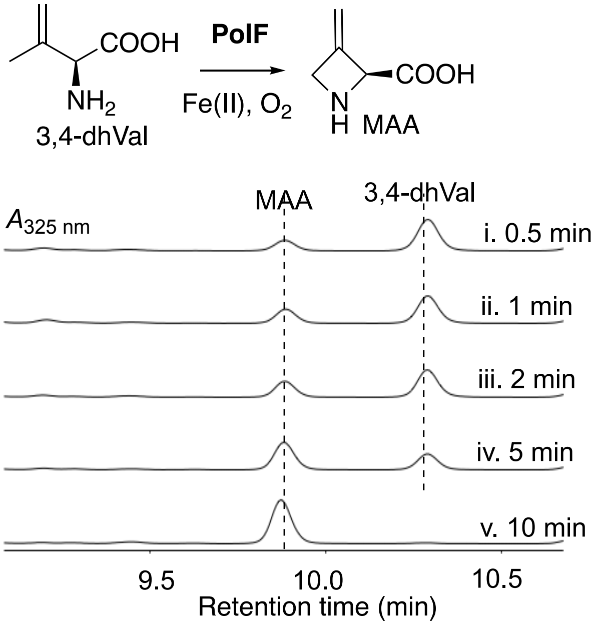

Figure S13. HPLC analysis of a PolF reaction with 3,4-dhVal. Shown are chromatograms monitoring absorbance at 325 nm for a PolF reaction with 3,4-dhVal quenched at 30 s (i), 1 min (ii), 2 min (iii), 5 min (iv), and 10 min. The assay was performed with 300 μM 3,4-dhVal, 30 μM PolF (apo form), 100 μM Fe(II), ~0.5 mM O_2, and 1 mM ascorbate.

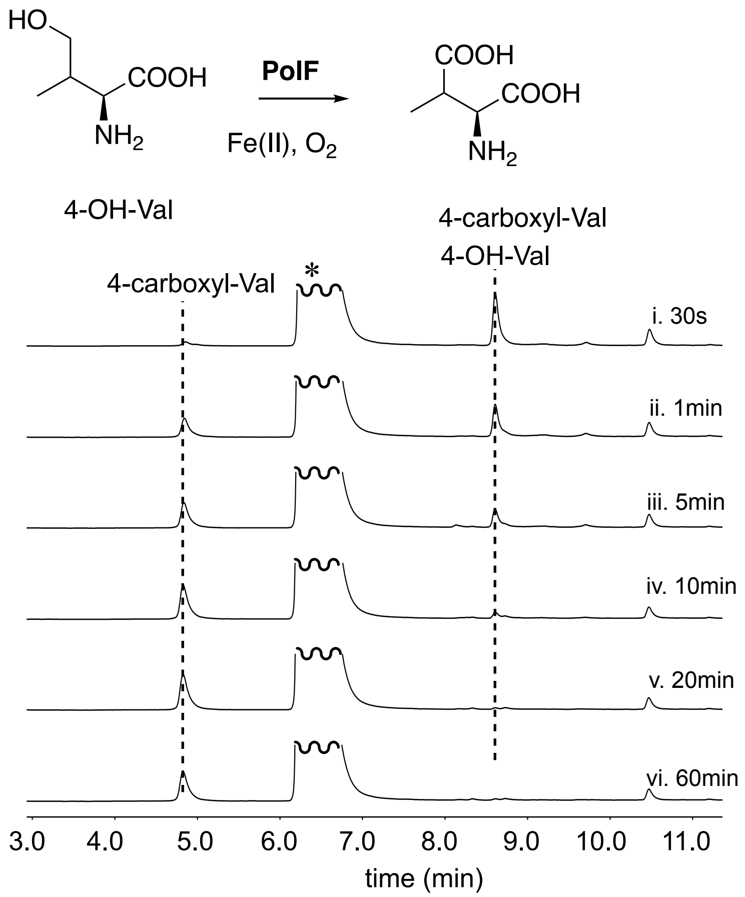

Figure S14. HPLC analysis of a PolF reaction with 4-OH-Val. Shown are chromatograms monitoring absorbance at 325 nm for a PolF reaction with 4-OH-Val quenched at 30 s (i), 1 min (ii), 5 min (iii), 10 min (iv), 20 min (v) and 60 min (vi). The assay was performed with 100 μM 4-OH-Val, 30 μM PolF (apo form), 100 μM Fe(II), ~0.5 mM O_2, and 1 mM ascorbate. * Unrelated peaks from DnsCl.

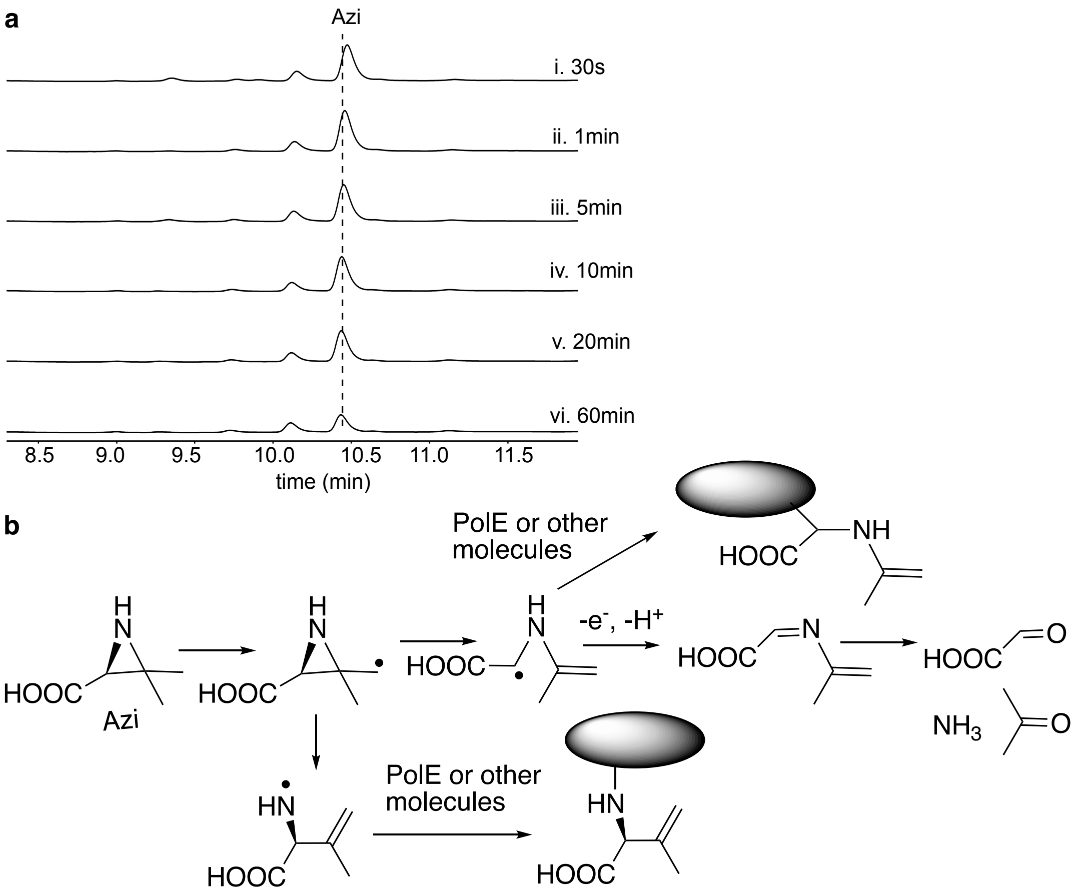

Figure S15. Characterization of a PolF reaction with Azi. **a.** Shown are HPLC chromatograms monitoring absorbance at 325 nm for a PolF reaction with AZI quenched at 30 s (i), 1 min (ii), 5 min (iii), 10 min (iv), 20 min (v) and 60 min (vi). The PolF reaction was performed with 300 μM Azi, 30 μM PolF (apo form), 100 μM Fe(II), ~0.5 mM O_2, and 1 mM ascorbate. **b.** Possible mechanisms of oxidative degradation of Azi. While we looked for these products by LCMS with or without derivatization by carbonyl reactive reagents, we have not been able to detect any of them.

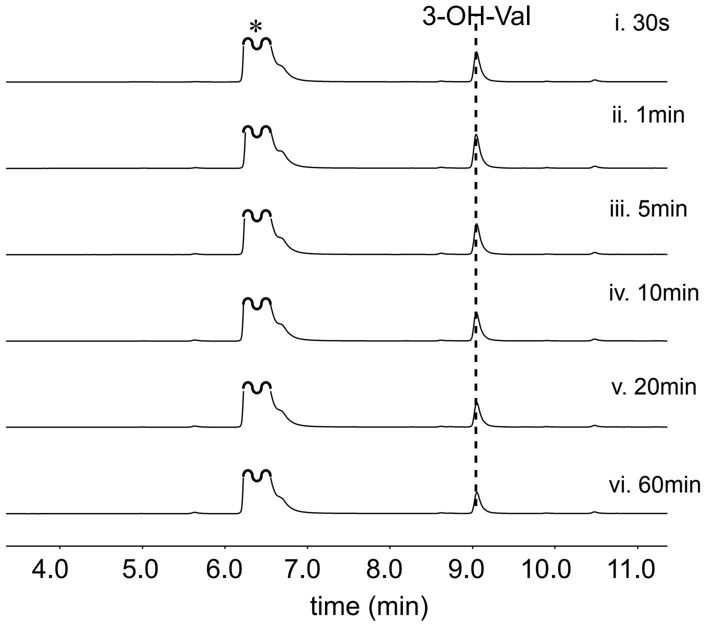

Figure S16. HPLC analysis of a PolF reaction with 3-OH-Val. Shown are chromatograms monitoring absorbance at 325 nm for PolF reactions with 3-OH-Val quenched at 30 s (i), 1 min (ii), 5 min (iii), 10 min (iv), 20 min (v), and 60 min (vi). The assay was performed with 100 μM 3-OH-Val, 30 μM PolF (apo form), 100 μM Fe(II), ~0.5 mM O_2, and 1 mM ascorbate. * Unrelated peaks from DnsCl.

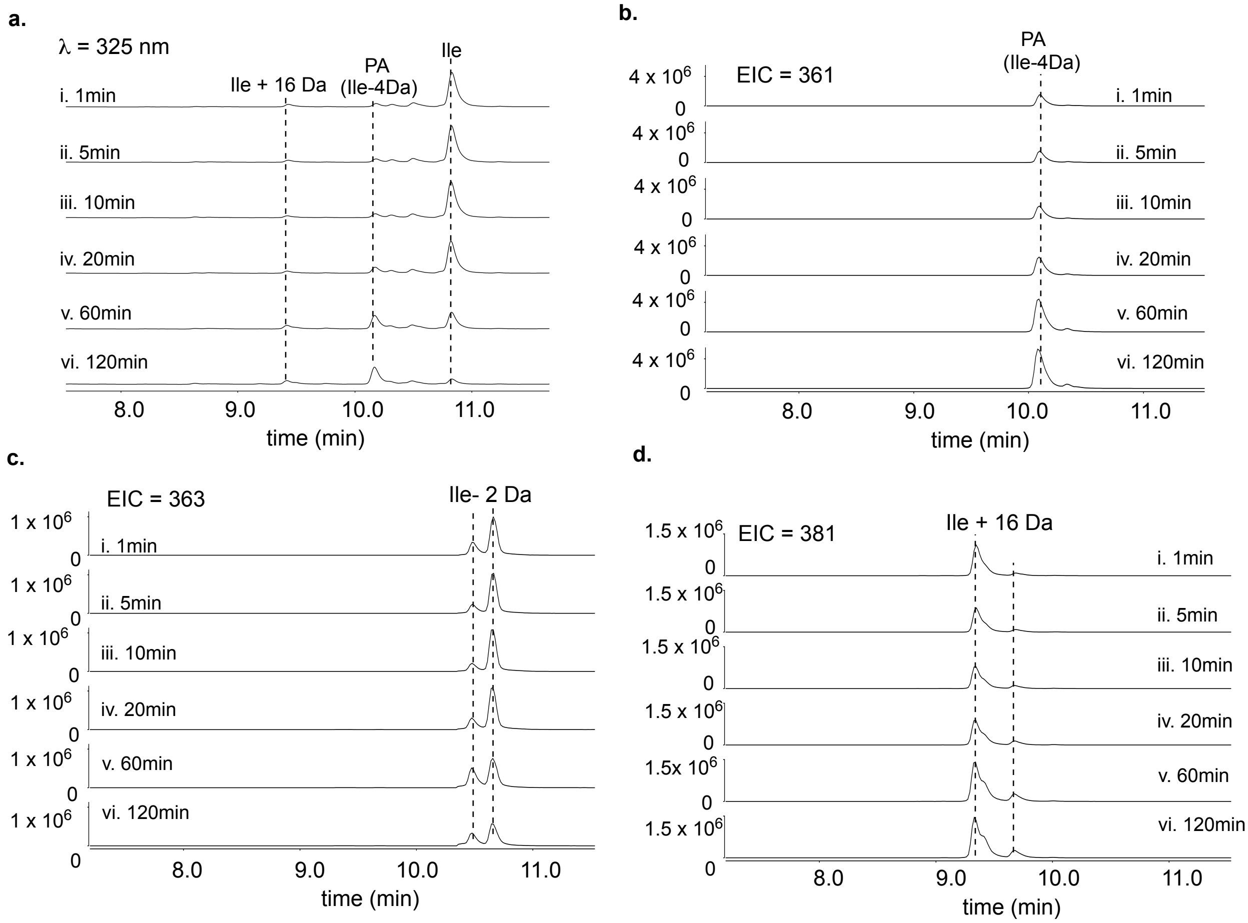

Figure S17. HPLC analysis of a PolF reaction with L-Ile under multiple turnover conditions. Shown are UV chromatograms monitoring absorbance at 325 nm (**a**), EIC = 361.1217 (**b**), EIC = 363.1372 (c) and EIC = 381.1485 (**d**) for PolF reactions with L-Ile quenched at 1 min (i), 5 min (ii), 10 min (iii), 20 min (iv), 60 min (v) and 120 min (vi). PolF full reaction was performed with 300 μM L-Ile, 30 μM PolF (apo form), 100 μM Fe(II) and ~0.5 mM O_2 and 1 mM ascorbate. Since the corresponding L-Ile derivatives (aziridine, desaturation, and hydroxylation) are not readily available and the amounts of enzyme assay products were too small for isolation and structural characterization by NMR, we characterized the products based on the kinetic behaviors and comparison with the PolF reaction with L-Val. The most abundant product had the -2 Da molecular weight with a retention time close to L-Ile, a similar relationship between L-Val and dh-Val. Under multiple turnover conditions, this -2 Da product accumulated in the early time points and was consumed as the reaction progressed. Thus, we propose this product as 3,4-dehydro-Ile (3,4-dhIle). This structural assignment is later confirmed by our PolE characterization. The second most abundant product in the single turnover reaction had a +16 Da molecular weight, corresponding to a hydroxylation. The position of hydroxylation remains ambiguous, but we tentatively assigned it to 4-hydroxy-Ile based on the formation of 4-OH-Val as the major side product in the L-Val reaction. The third most abundant product had -2 Da, which we assigned as an aziridine derivative because this compound migrated earlier than 3,4-dhIle in our LC-MS analysis and accumulated minimally under multiple turnover conditions, behaviors similar to Azi in L-Val

reaction. Finally, the second +16 Da product was assigned to 3-OH-Ile based on the minimal formation in both single and multiple turnover conditions, and the formation of 3-OH-Val in the L-Val reaction.

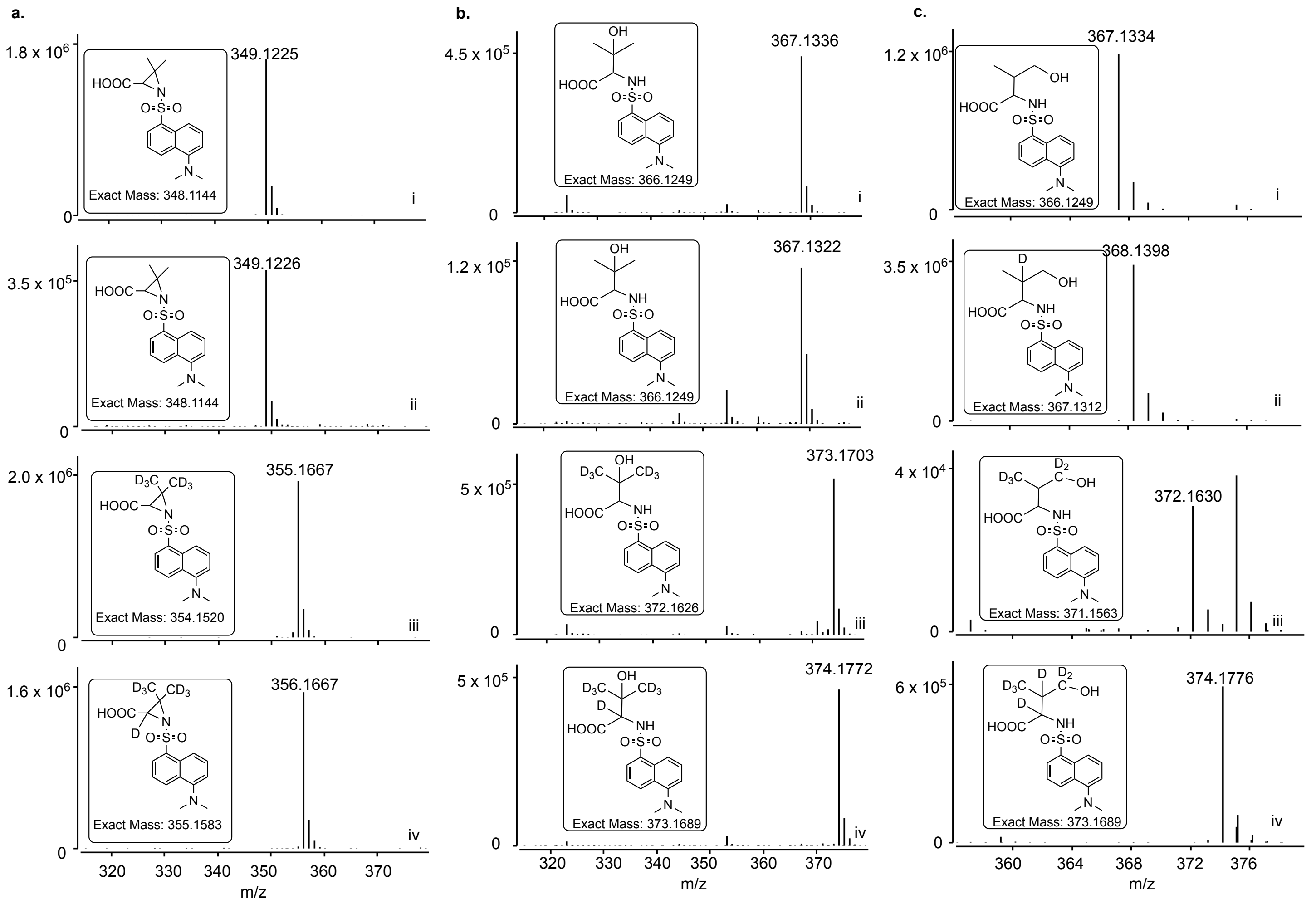

Figure S18. LC-MS analysis of products from PolF reaction with L-Val and its isotopologs. Shown are the mass spectra of Azi (**a**), 3-OH-Val (**b**), and 4-OH-Val (**c**) in the reactions with L-Val (i), with [3-D]Val (ii), with [4,4'-D_6]Val (iii), and with [U-D_8]Val (iv).

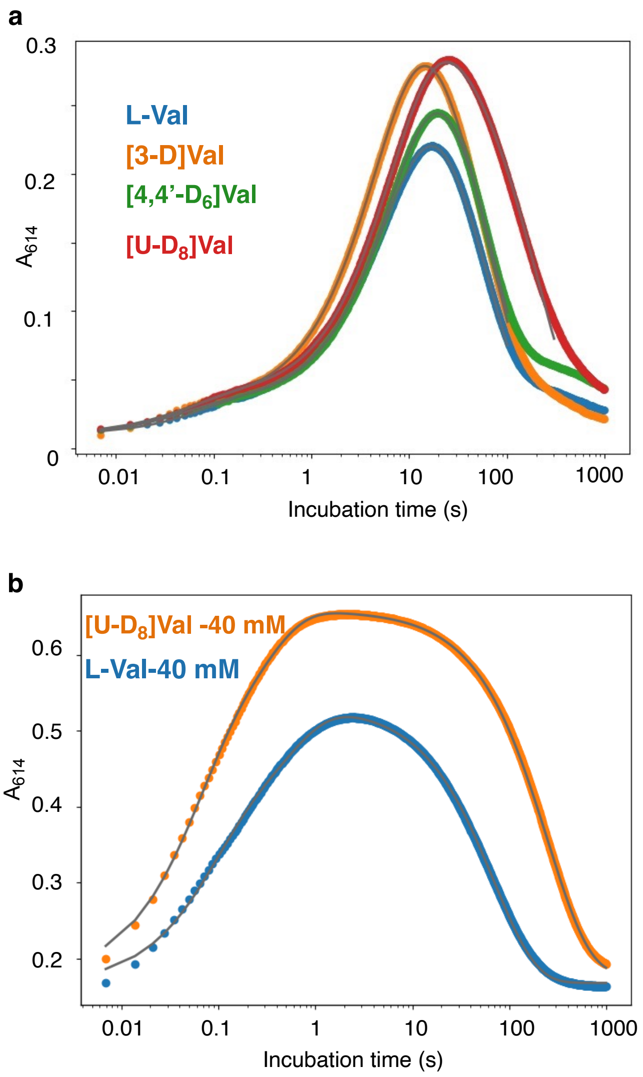

Figure S19. Stopped-flow analysis of O_2 activation by PolF. **a.** 1 mM L-Val or deuterated Val was used as the substrate. **b.** 40 mM L-Val or [U-D_8]Val was used as the substrate. The solid lines are non-linear curve fit of equation 2 (see Methods).

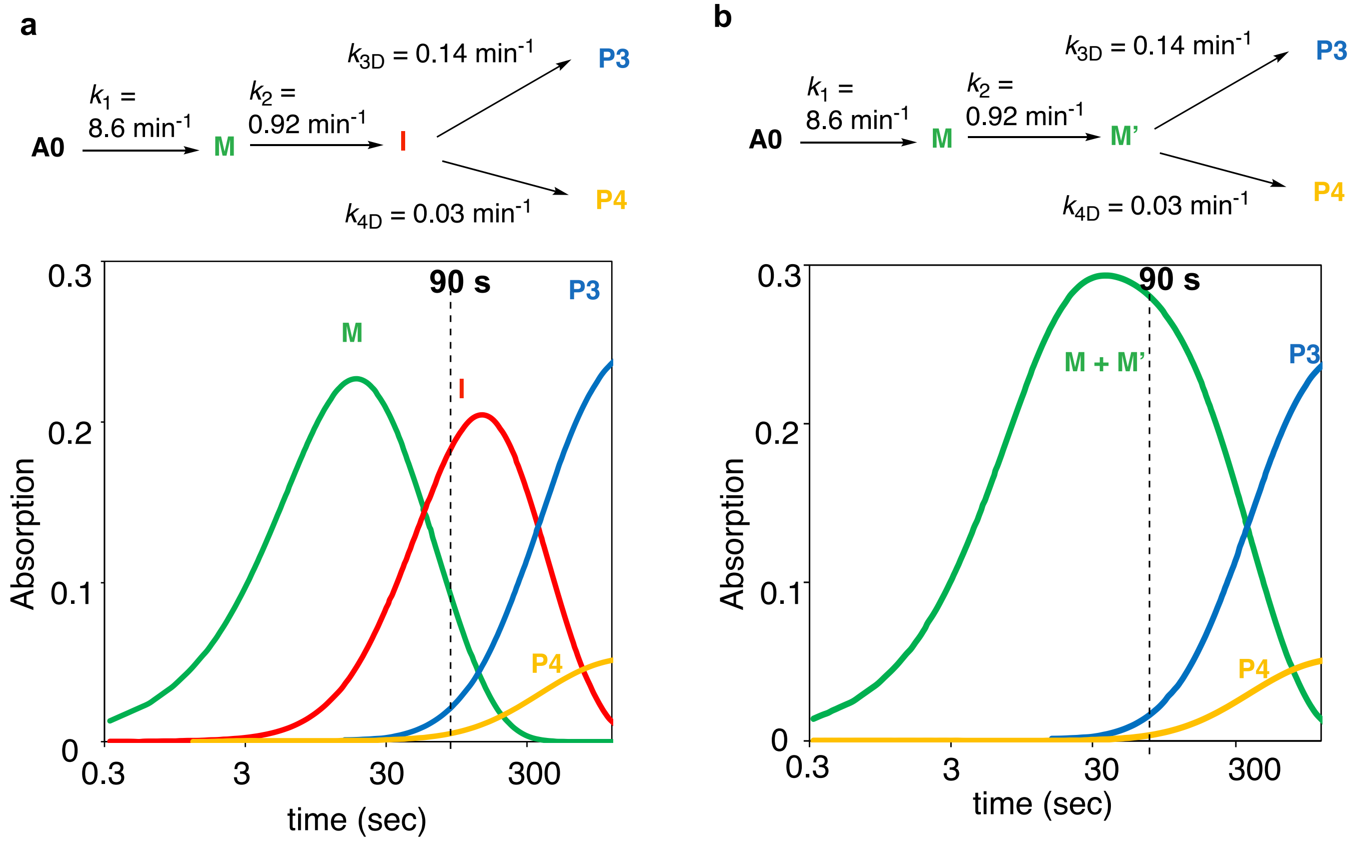

Figure S20. Kinetic simulation of the PolF reaction with [U-D_8]Val under the conditions for the Stopped-Flow experiments: 0.3 mM PolF and 1 mM [U-D_8]Val. **a.** Kinetic simulation of a reaction model with an oxidative intermediate (I) spectroscopically distinct from μ-peroxo-Fe(III)$_2$ (**M**). In this model, PolF with a Fe(II)$_2$ cluster (**A0**) is first converted into μ-peroxo-Fe(III)$_2$ (**M**) and then to a putative oxidative intermediate responsible for the H-atom abstraction (I) to yield products through 3-H and 4-H abstraction (**P3** and **P4**, respectively). **b.** Kinetic simulation of a reaction model, in which Fe(II)$_2$ (**A0**) is first converted into μ-peroxo-Fe(III)$_2$ (M) and then to a spectroscopically indistinguishable another μ-peroxo-Fe(III)$_2$ (**M′**) to yield products from 3-H and 4-H abstraction (**P3** and **P4**, respectively). This model implies that μ-peroxo-Fe(III)$_2$ is responsible for the H-atom abstraction and the slow step preceding the H-atom abstraction (k_2, **M** → **M′**) does not alter the chemical structure of μ-peroxo-Fe(III)$_2$. Both simulations were performed with the rate constants for the formation of μ-peroxo-Fe(III)$_2$ with 1 mM L-Val determined by stopped-flow (k_1), the rate-determining step preceding the H-atom abstraction determined by stopped-flow with unlabeled L-Val (k_{obs}), and the D-atom abstraction in the reaction with [U-D_8]Val (k_{3D} and k_{4D}) (see Supplementary Table 4). The kinetic simulation was performed using KinTek Explorer 11.1.1. Dashed lines indicate the 90-sec time point characterized by Mössbauer spectroscopy in Extended Data Fig. 4.

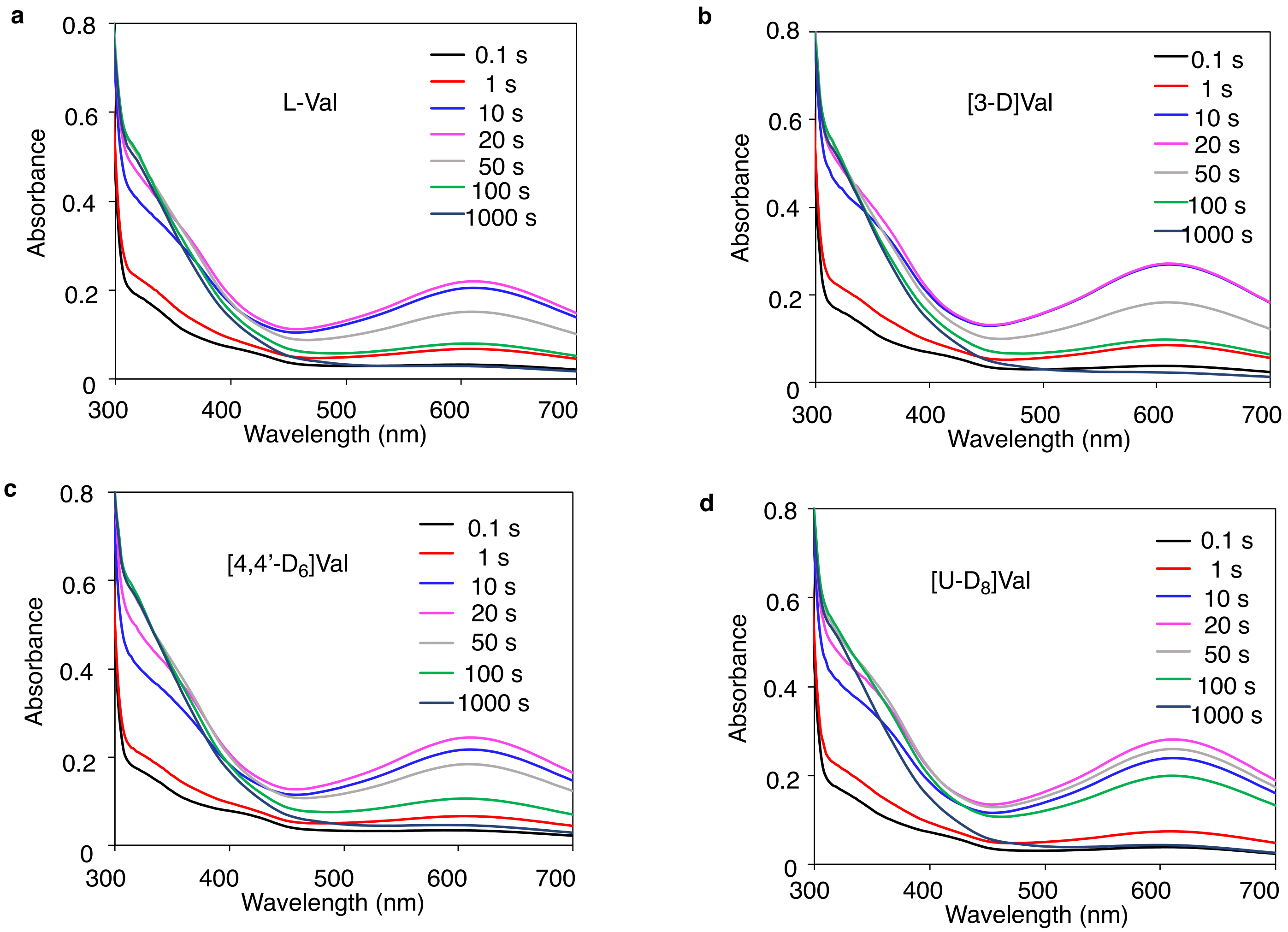

Figure S21. Stopped-flow analysis of the PolF reaction with deuterated L-Val. Absorption spectra acquired after a rapid mixing at 5 °C of an anoxic solution of PolF (0.3 mM) and Fe(II) (0.6 mM, 2 molar equivalent) in the presence of 1 mM L-Val (**a**), 1 mM [3-D]Val (**b**), 1 mM [4,4'-D_6]Val (**c**) or 1 mM [U-D_8]Val (**d**) with an equal volume of O_2-saturated buffer.

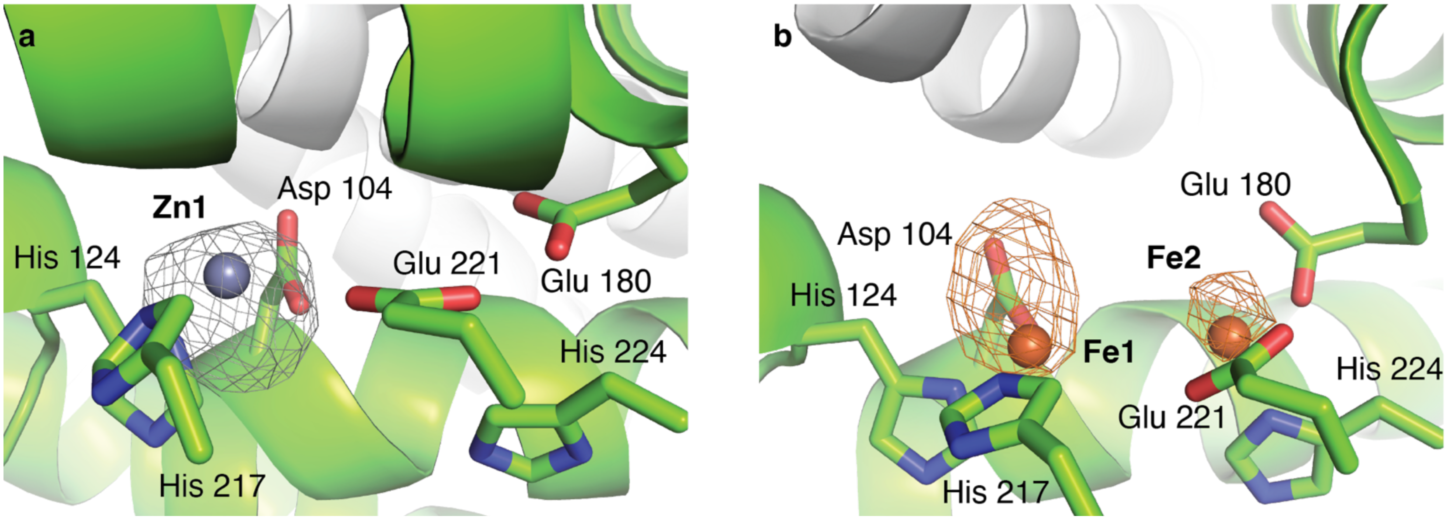

Figure S22. Anomalous diffraction electron density difference maps of PolF. (**a**) Anomalous diffraction electron density difference map (gray mesh, contoured at 5.0σ) collected at the Zn x-ray absorption edge.
(**b**) Anomalous diffraction electron density difference map (gray mesh, contoured at 3.2s) collected at the Fe x-ray absorption edge.

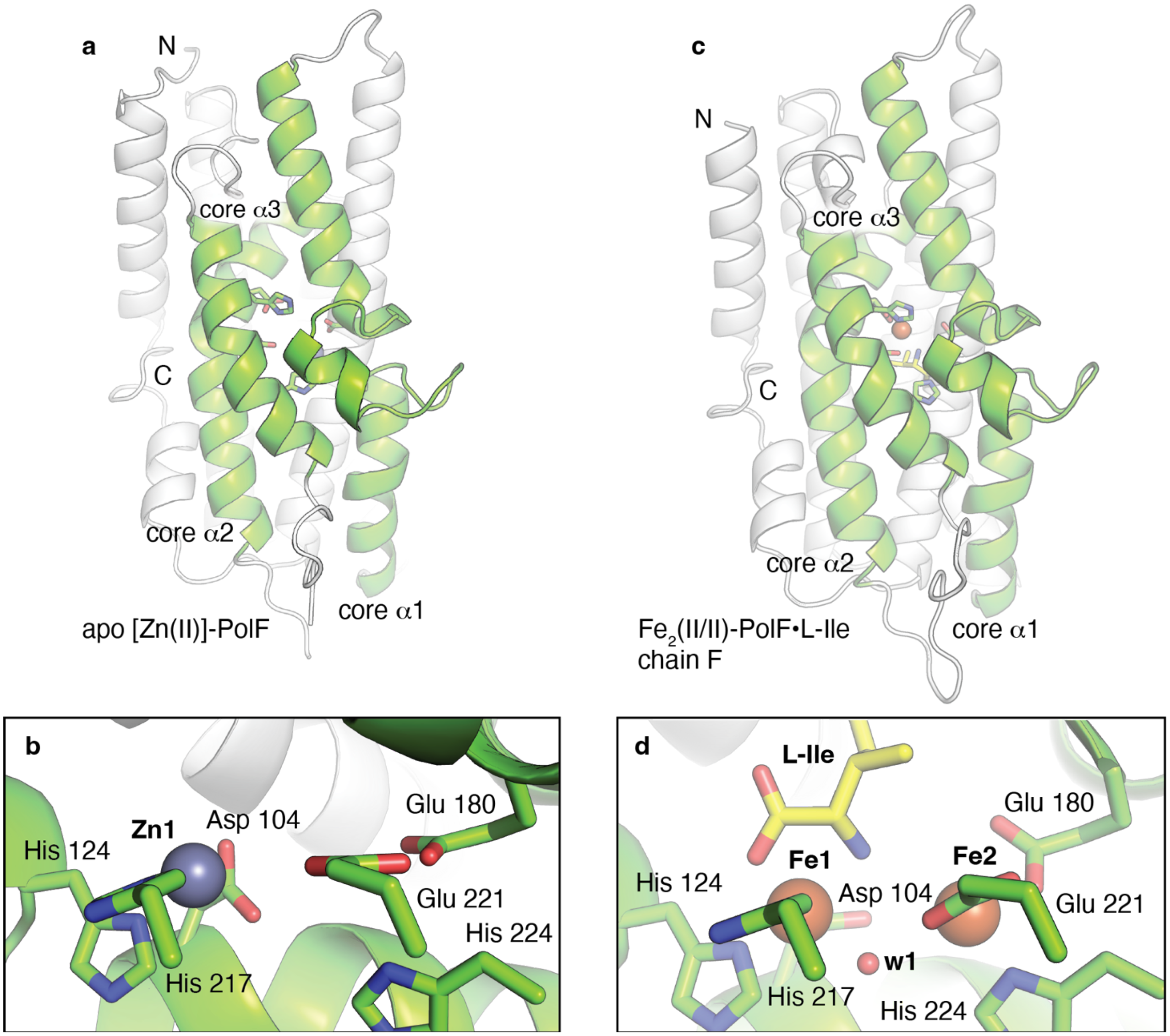

Figure S23. Comparison of apo [Zn(II)]-PolF and Fe_2(II/II)-PolF•L-Ile structures. (**a**), (**c**) The overall structures shows no significant differences between the mono-metallated and holo structures. (**b**), (**d**) A comparison of the metal-binding sites shows only minor changes in amino acid position between the two structures. Based on occupancy of the substrate binding site by glycerol in certain chains of the Fe_2(II/II)-PolF•L-Ile complex, we hypothesize that glycerol in the PolF storage buffer may induce folding of core a3. Glycerol occupancy of the active site may also be a factor in retention of Zn(II) in metal-binding site 1 in the apo preparation used to solve the initial structures.

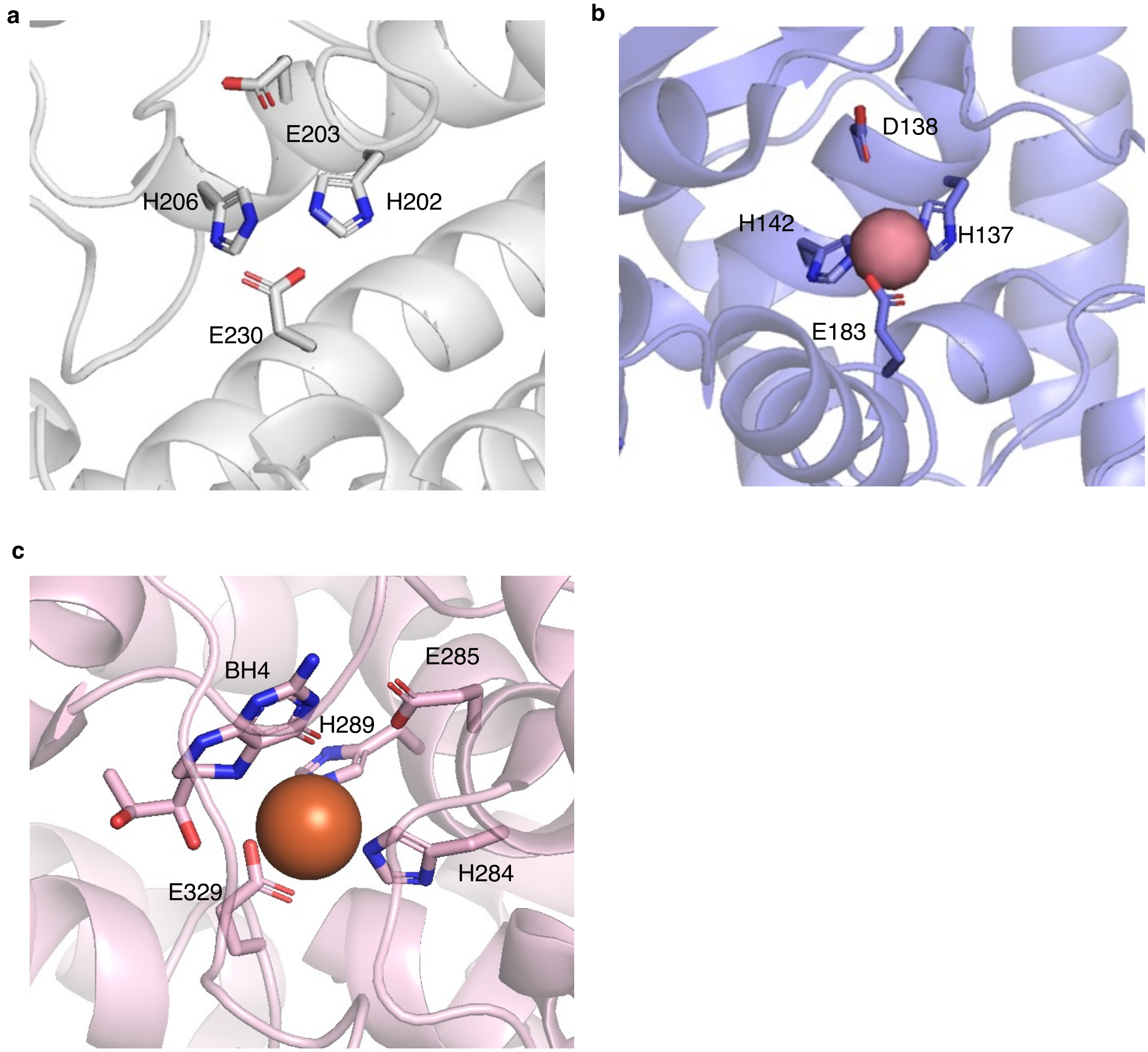

Figure S24. Active sites of PolE AlphaFold model and pterin/Fe dependent hydroxylase. (**a**) Predicted metal binding sites of PolE by AlphaFold 2[16], (**b**) Metal binding site of bacterial phenylalanine hydroxylase (PDB: 3TCY)[17], (**c**) Metal and pterin binding of human phenylalanine hydroxylase (PDB: 1kw0)[18].

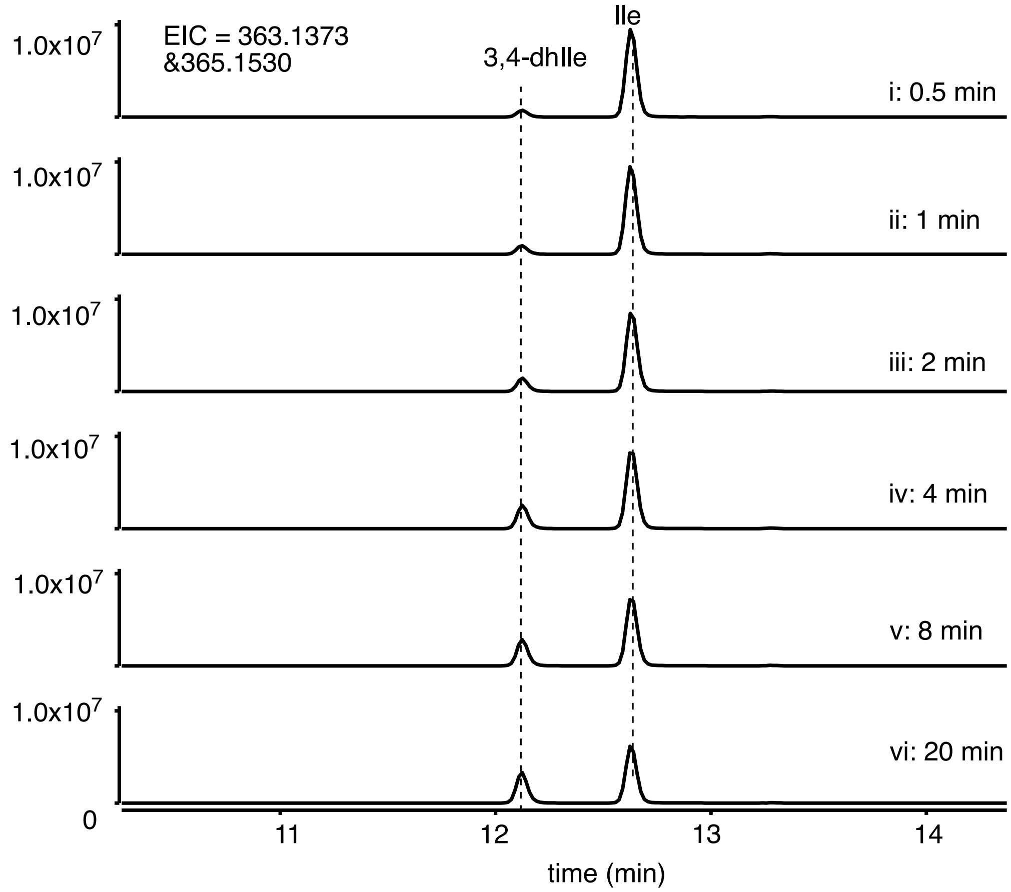

Figure S25. LC-MS analysis of the PolE time course assay. LC-MS analysis (EIC at *m/z* 363.1373 and 365.1530) of the PolE reaction quenched at 0.5 min (i), 1 min (ii), 2 min (iii), 4 min (iv), 8 min (v) and 20 min (vi). The PolE assay was performed with 150 μM L-Ile, 15 μM PolE, 100 μM Fe(II), 1 mM ascorbate, 100 μM BH_4, 0.5 μM QDPR, 1mM NADH and ~0.5 mM O_2.

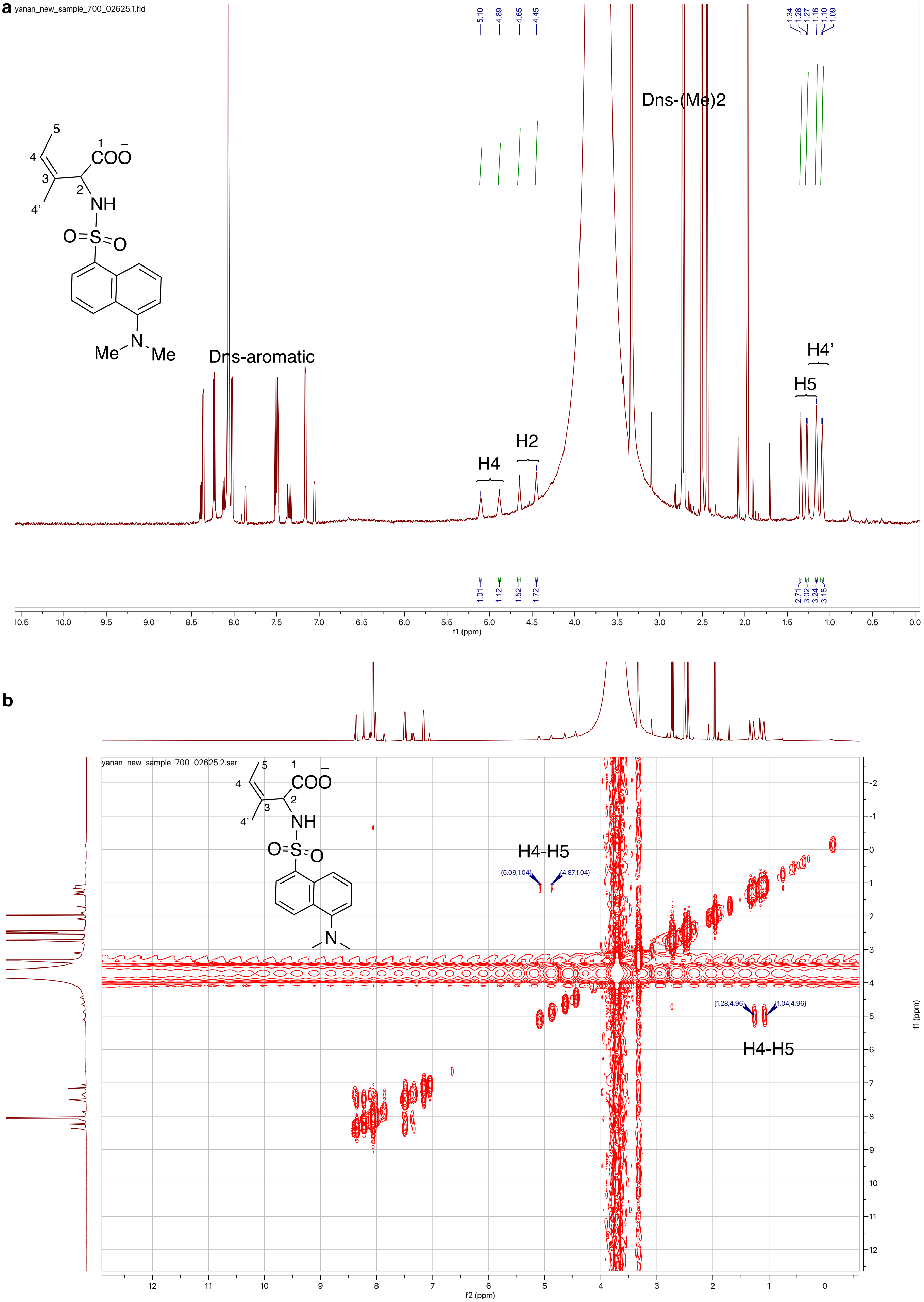
a
yanan_new_sample_700_02625.1.fid
Dns-(Me)2
Dns-aromatic
H4
H2
H5
H4'
COO
NH
Me
f1 (ppm)
b
yanan_new_sample_700_02625.2.ser
H4-H5
{5.09,1.04}
{4.87,1.04}
{1.28,4.96}
{1.04,4.96}
f2 (ppm)
f1 (ppm)

c

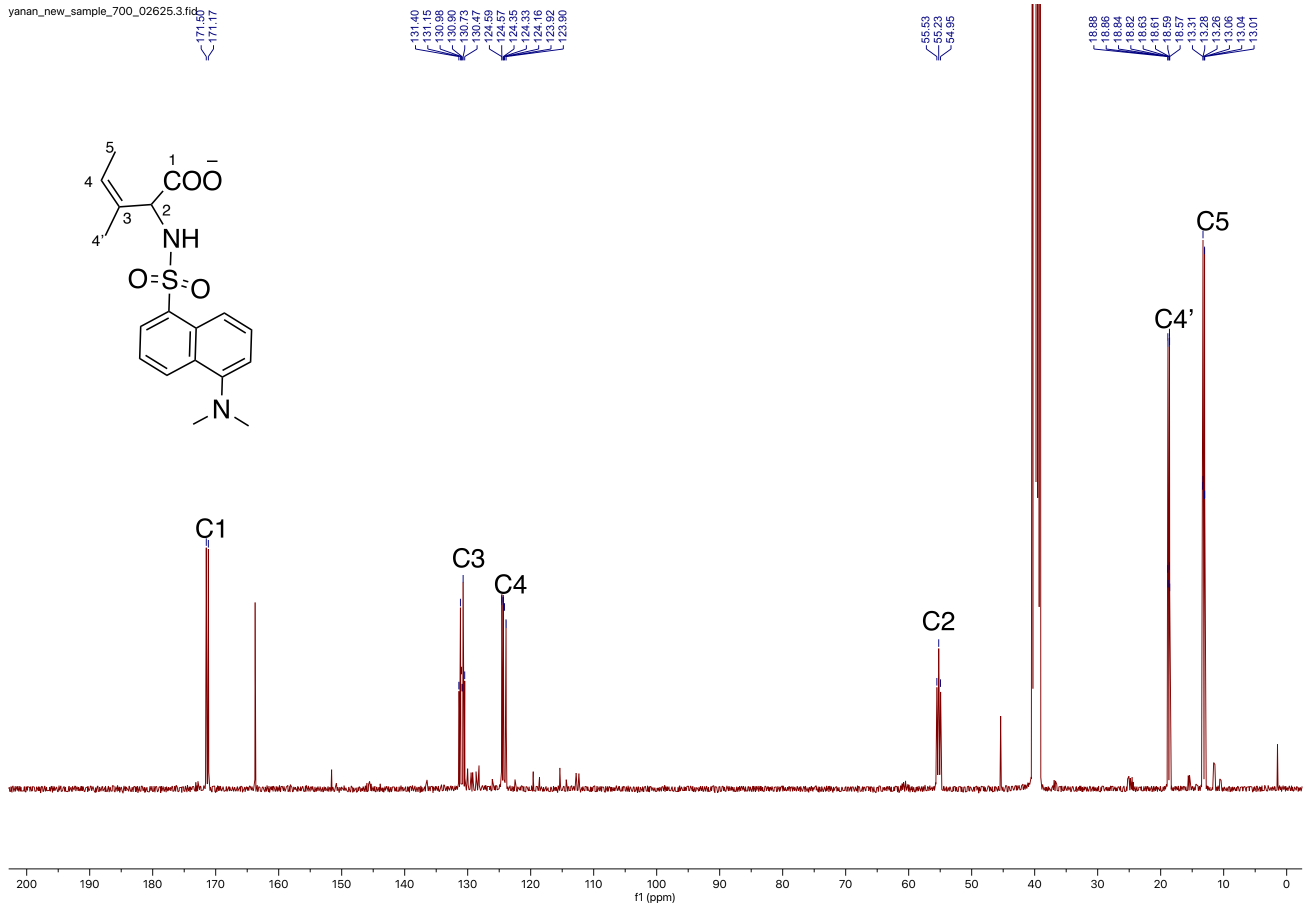
yanan_new_sample_700_02625.3.fid
C1
C3
C4
C2
C4'
C5
f1 (ppm)

d

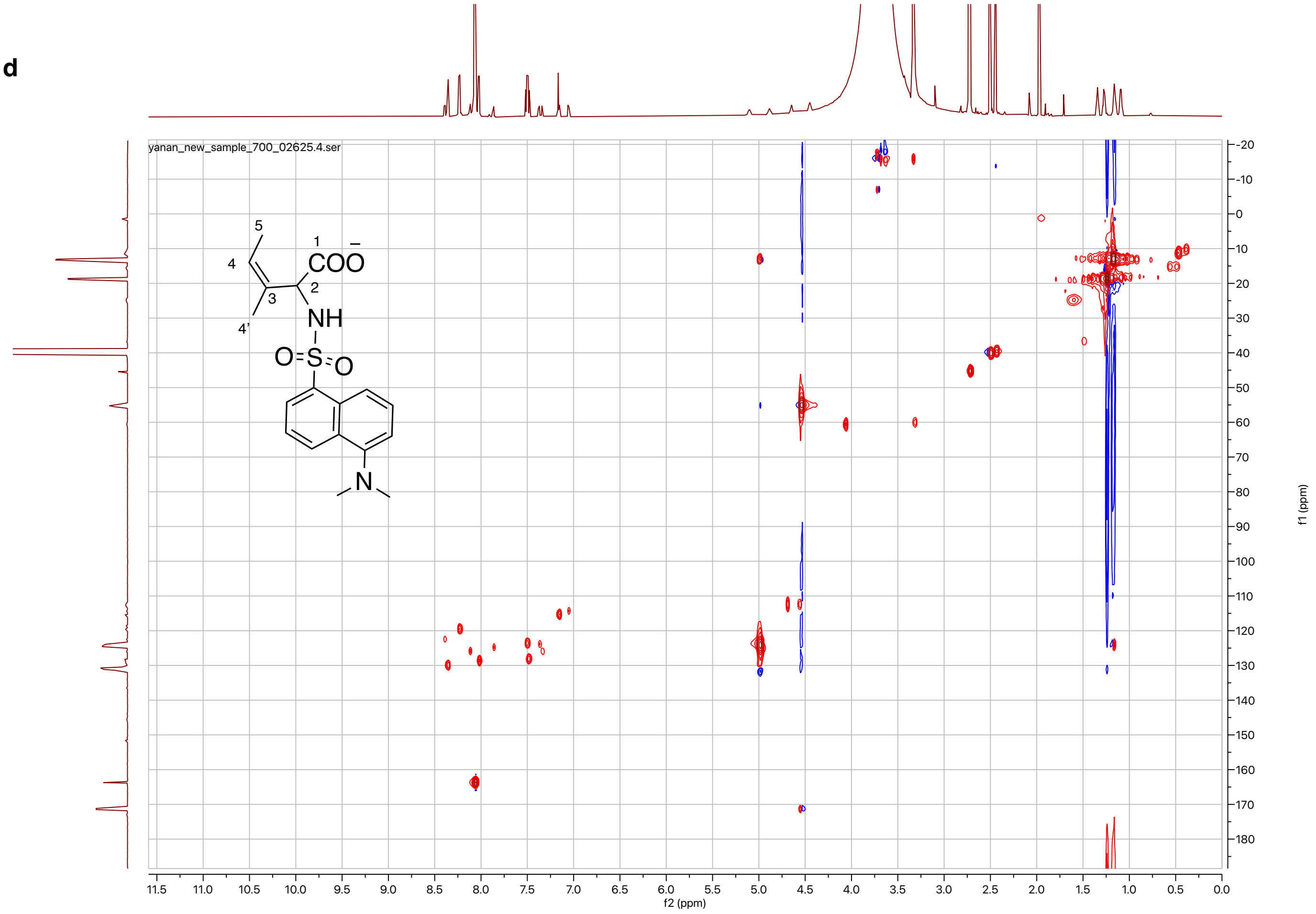
yanan_new_sample_700_02625.4.ser
f2 (ppm)
f1 (ppm)

e

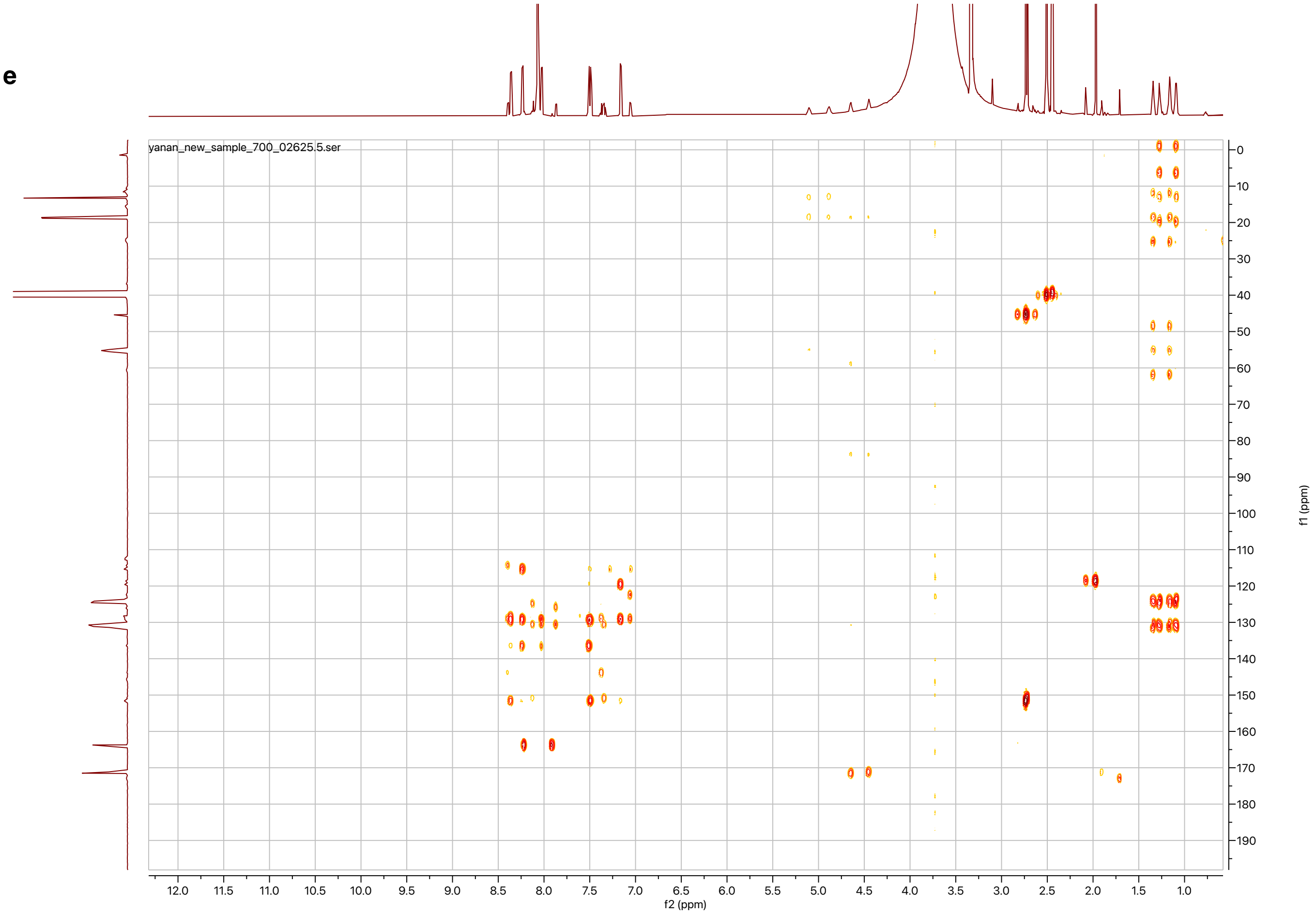

Figure S26. NMR characterization of Dns-3,4-dhIle(13C) at 700 MHz in d6-DMSO. **a.** ^{1}H NMR spectrum, **b.** ^{1}H-^{1}H COSY spectrum, **d.** ^{1}H-^{13}C HSQC spectrum, **e.** ^{1}H-^{13}C HMBC spectrum.

a.

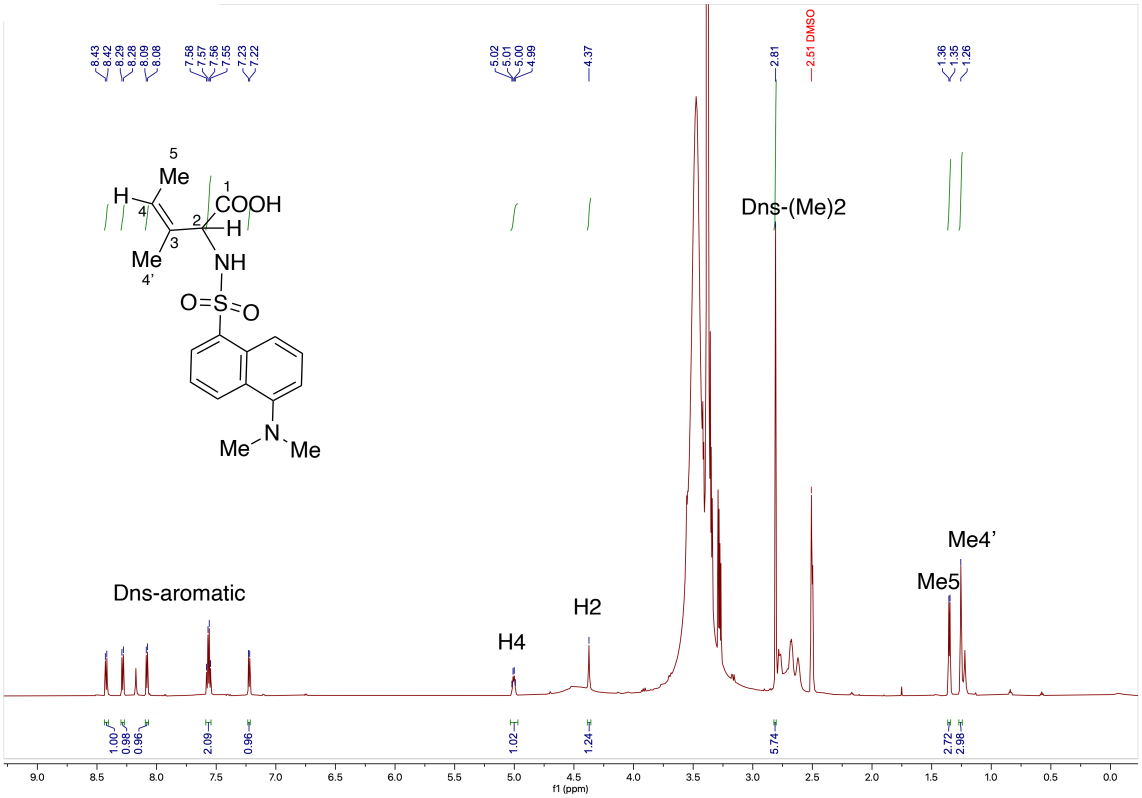

b.

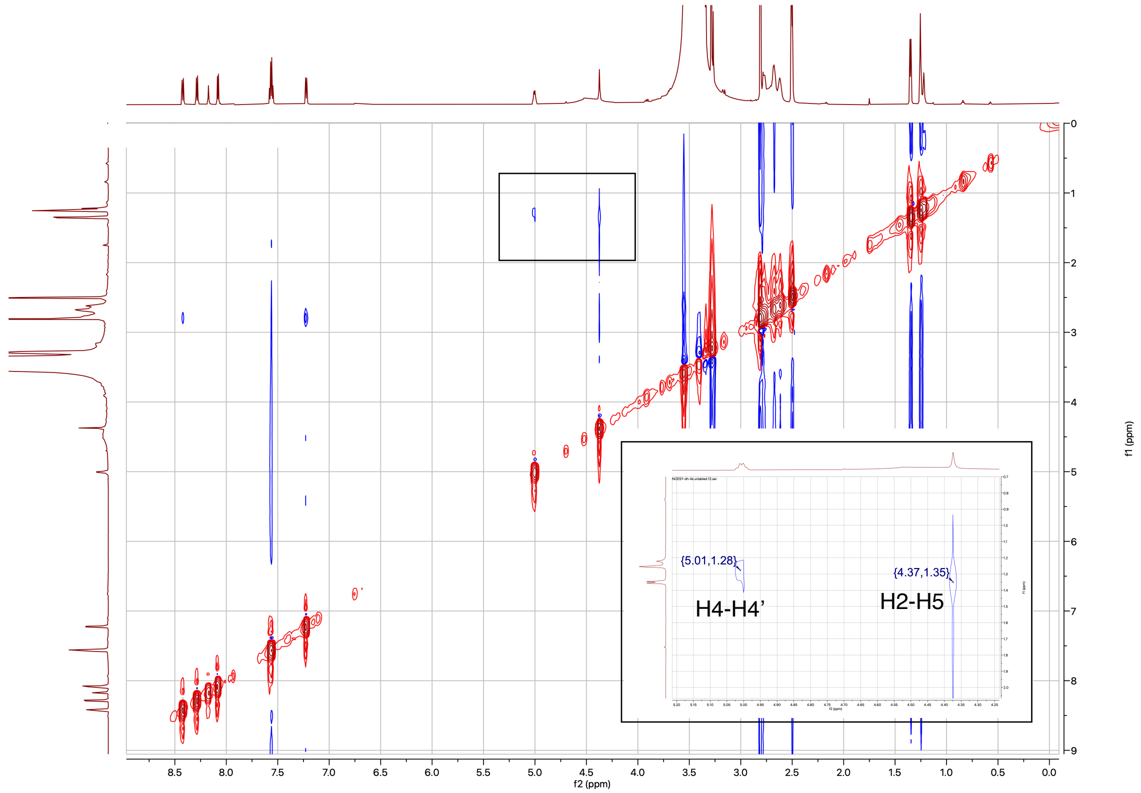

Figure S27. NMR characterization of Dns-dh-Ile at 700 MHz in d6-DMSO. **a.** ^{1}H NMR spectrum, **b.** ^{1}H-^{1}H NOESY NMR spectrum. The chemical shift changes of H2 and H4’ are attributed to pH differences caused by the presence of less formic acid in the mixture.

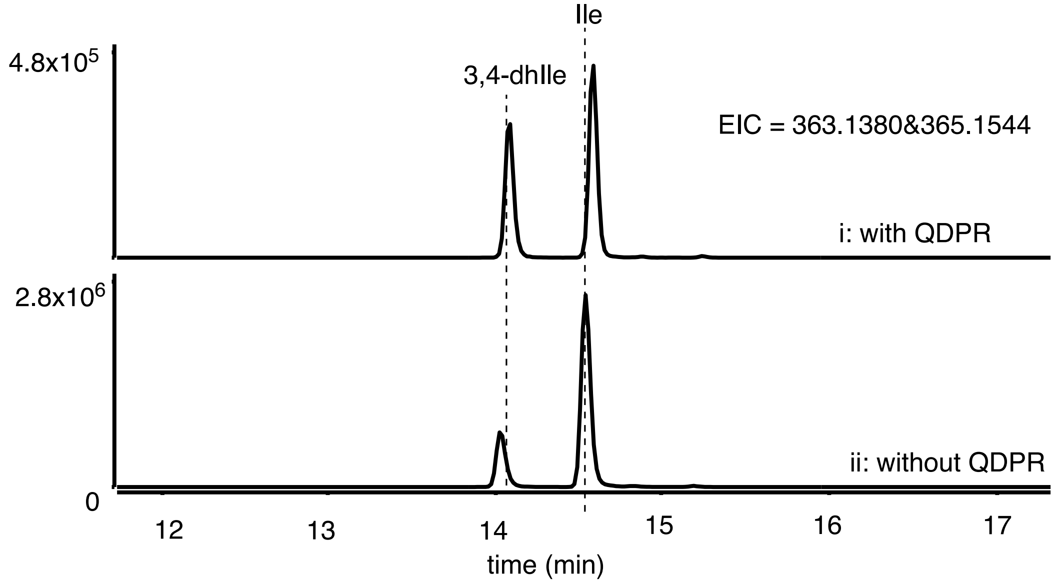

Figure S28. LC-MS analysis of PolE assays with QDPR (EIC = 363.1373 and 365.1530). The assays were performed with 150 μM L-Ile, 15 μM PolE, 100 μM Fe(II), 1mM ascorbate and ~0.5 mM O_2, with 1 mM NADH, 100 μM BH_4 and 0.5 μM QDPR (i) and with 1 mM BH_4 (ii).

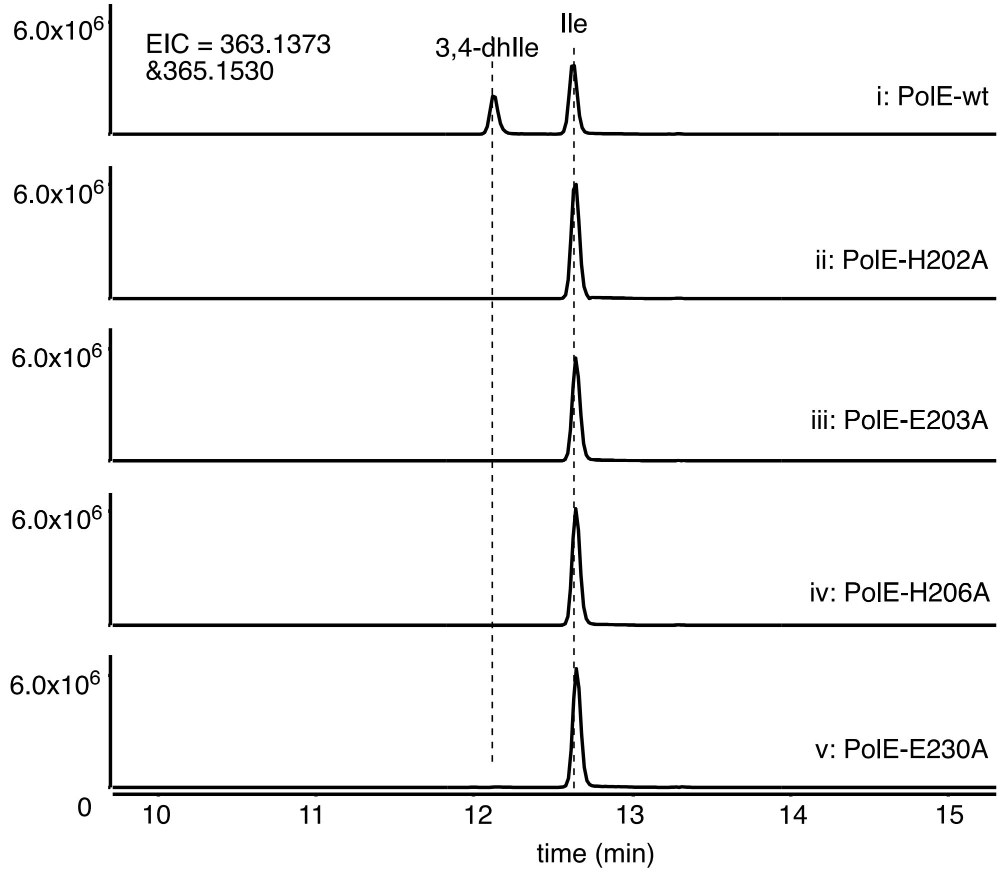

Figure S29. LC-MS analysis of PolE assays with different PolE mutants (EIC = 363.1373 and 365.1530). The assays were performed with 150 μM L-Ile, 15 μM PolE mutants, 100 μM Fe(II), 1 mM ascorbate, and ~0.5 mM O_2, with PolE-WT (i), with PolE-H202A (ii), with PolE-E203A (iii), with PolE-H206A (iv), and with PolE-E230A (v).

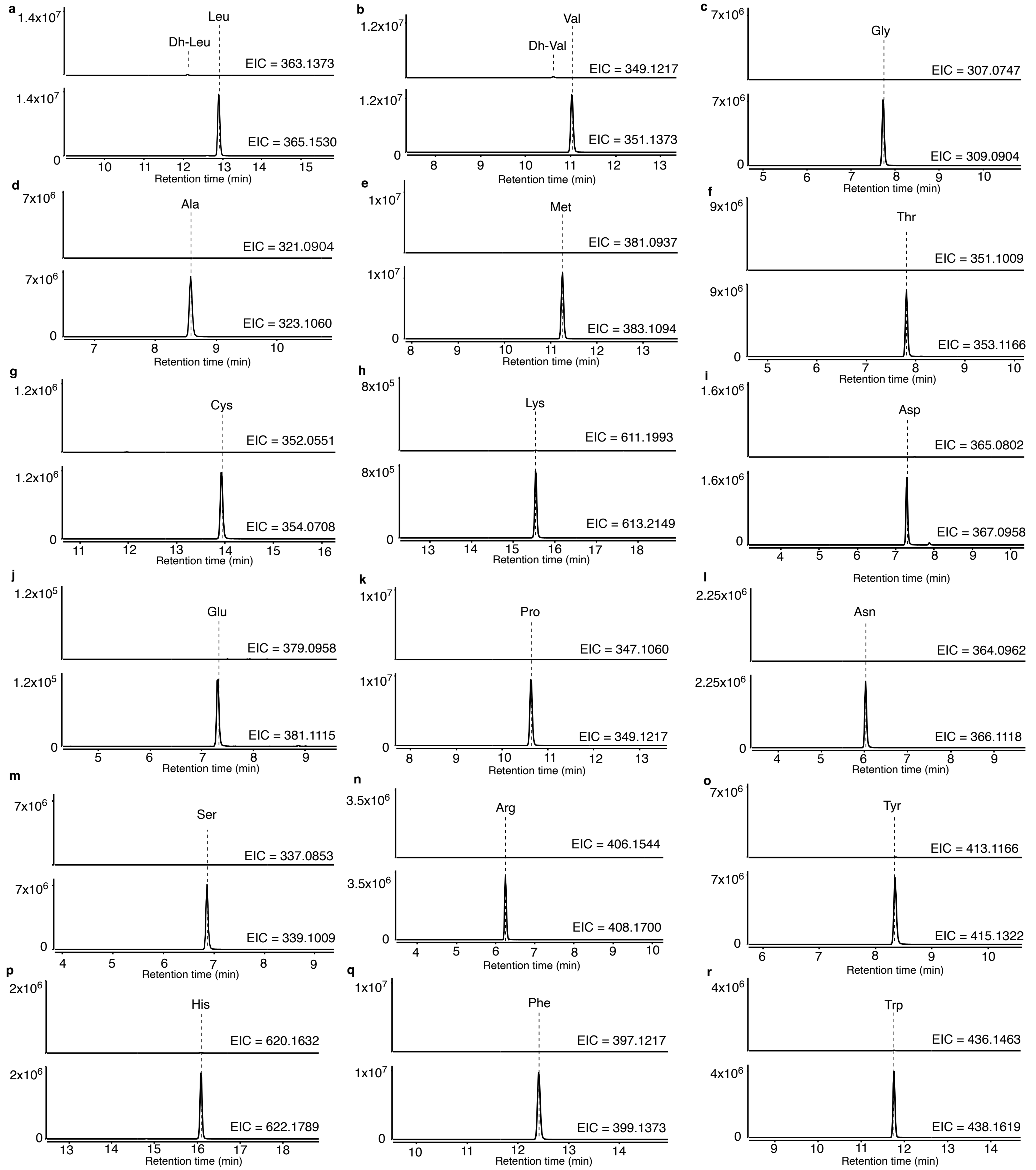

Figure S30. LC-MS analysis of PolE assays with proteogenic amino acids. L -Leu (**a**), L -Val (**b**), L-Gly(**c**), L-Ala(**d**), L-Met (**e**), L-Thr(**f**), L-Cys(**g**), L-Lys(**h**), L-Asp(**i**), L-Glu(**j**), L-Pro(**k**), L-Asn(**l**), L-Ser(**m**), L-Arg(**n**), L-Tyr(**o**), L-His(**p**), L-Phe(**q**) and L-Trp(**r**). Trace (i) in each panel shows the analysis of an assay with 150 μM L-amino acid, 15 μM PolE, 100 μM Fe(II), 1 mM ascorbate, 100 μM BH_4, 0.5 μM QDPR, 1mM NADH and ~0.5 mM O_2.

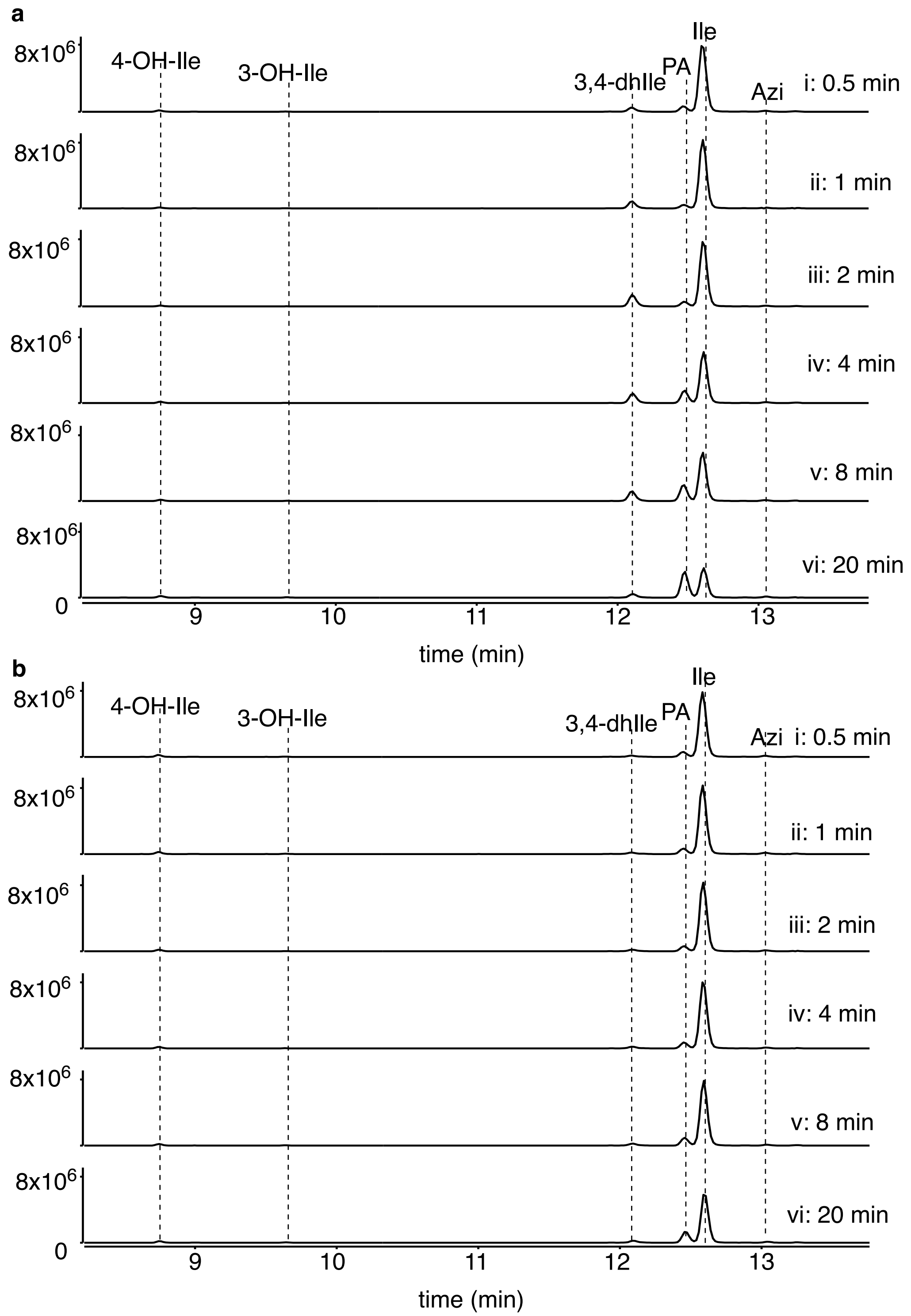

Figure S31. Time course analysis of PolE/PolF coupled assay. LC-MS analysis of the time course analysis (EIC = 361.1217, 363.1373, 365.1530 and 381.1479) of the PolEF coupled reaction (**a**) or PolF reaction alone (**b**) quenched at 0.5 min (i), 1 min (ii), 2 min (iii), 4 min (iv), 8 min (v) and 20 min (vi). PolEF coupled assay contains 150 μM L-Ile, 15 μM PolE, 15 μM PolF, 100 μM Fe(II), 100 μM BH_4, 1 mM ascorbate, 1mM NADH, 0.5μM QDPR and ~0.5 mM O_2. PolF assay contains all the above, except for 15 μM PolE.

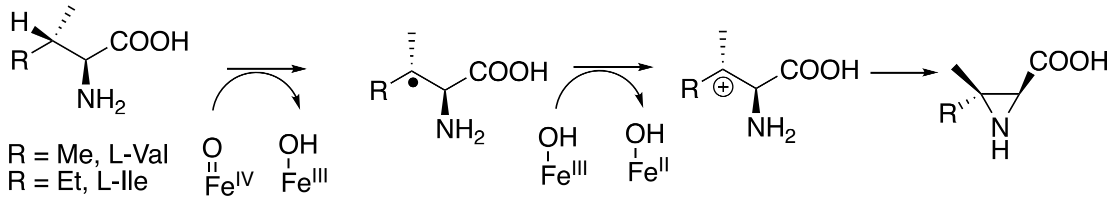

Figure S32. Proposed mechanism of Aziridine formation by TqaL[19].

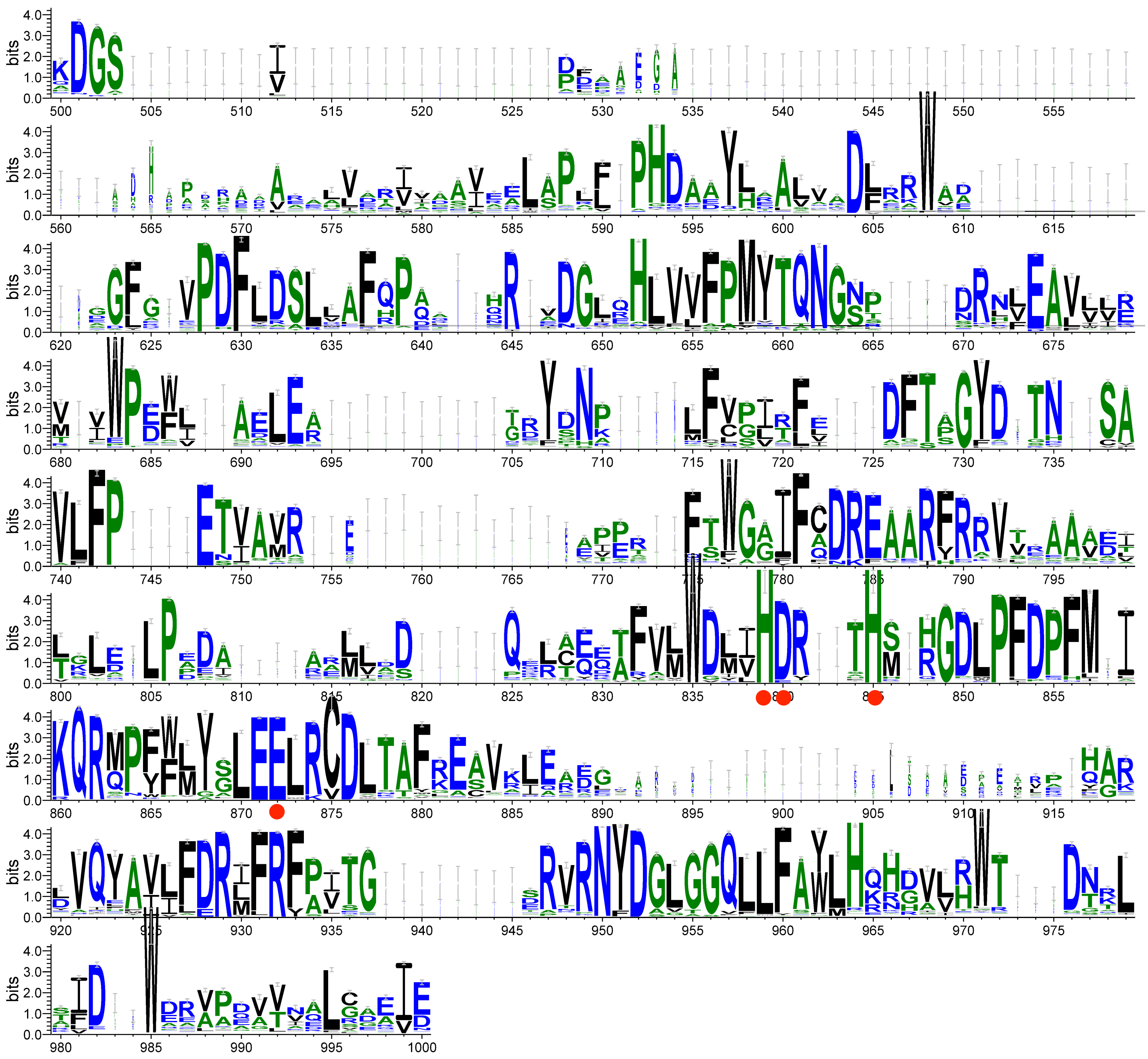

Figure S33. Sequence logo of DUF6421. The UniRef90 sequences of DUF6421 were obtained from UniProt database. The HD/ExxHx$_n$E motif is highlighted with red circles.

References

1. Ng, T. L., Rohac, R., Mitchell, A. J., Boal, A. K. & Balskus, E. P. An N -nitrosating metalloenzyme constructs the pharmacophore of streptozotocin. *Nature* **566**, 94–99 (2019).
2. McBride, M. J. *et al.* Substrate-triggered μ-peroxodiiron(III) intermediate in the 4-chloro-l-lysine-fragmenting heme-oxygenase-like diiron oxidase (HDO) BesC: substrate dissociation from, and C4 targeting by, the intermediate. *Biochemistry* **61**, 689–702 (2022).
3. Zhang, B. *et al.* Substrate-triggered formation of a peroxo-Fe2(III/III) intermediate during fatty acid decarboxylation by UndA. *J. Am. Chem. Soc.* **141**, 14510–14514 (2019).
4. McBride, M. J. *et al.* Structure and assembly of the diiron cofactor in the heme-oxygenase–like domain of the N-nitrosourea–producing enzyme SznF. *Proc. Natl. Acad. Sci.* **118**, e2015931118 (2021).
5. Zhao, Y. & Xia, W. Recent advances in radical-based C–N bond formation via photo-/electrochemistry. *Chem. Soc. Rev.* **47**, 2591–2608 (2018).
6. Raps, F. C., Rivas-Souchet, A., Jones, C. M. & Hyster, T. K. Emergence of a distinct mechanism of C–N bond formation in photoenzymes. *Nature* **637**, 362–368 (2025).
7. Draelos, M. M., Thanapipatsiri, A., Sucipto, H. & Yokoyama, K. Cryptic phosphorylation in nucleoside natural product biosynthesis. *Nat. Chem. Biol.* **17**, 213–221 (2021).
8. Paget, M. S. B., Chamberlin, L., Atrih, A., Foster, S. J. & Buttner, M. J. Evidence that the extracytoplasmic function sigma factor ςE is required for normal cell wall structure in streptomyces coelicolor A3(2). *J. Bacteriol.* **181**, 204–211 (1999).
9. MacNeil, D. J. *et al.* Analysis of *streptomyces avermitilis* genes required for avermectin biosynthesis utilizing a novel integration vector. *Gene* **111**, 61–68 (1992).
10. Aínsa, J. A. Practical streptomyces genetics. T. Kieser, M. J. Bibb, M. J. Buttner, K. F. Chater, D. A. Hopwood. *Int. Microbiol.* **3**, 260–261 (2000).
11. Thanapipatsiri, A., Claesen, J., Gomez-Escribano, J.-P., Bibb, M. & Thamchaipenet, A. A streptomyces coelicolor host for the heterologous expression of type III polyketide synthase genes. *Microb. Cell Factories* **14**, 145 (2015).
12. fish, W. W. [27] rapid colorimetric micromethod for the quantitation of complexed iron in biological samples. in *Methods in Enzymology* vol. 158 357–364 (Academic Press, 1988).
13. LESIV, A. V. *et al.* Magnetic resonance imaging drug containing a deuterated natural branched-chain amino acid, and diagnostic method using said drug. (2022).
14. Bierman, M. *et al.* Plasmid cloning vectors for the conjugal transfer of DNA from *escherichia coli* to *streptomyces* spp. *Gene* **116**, 43–49 (1992).
15. Hong, H.-J., Hutchings, M. I., Hill, L. M. & Buttner, M. J. The role of the novel fem protein VanK in vancomycin resistance in streptomyces coelicolor*. *J. Biol. Chem.* **280**, 13055–13061 (2005).
16. Jumper, J. *et al.* Highly accurate protein structure prediction with AlphaFold. *Nature* **596**, 583–589 (2021).
17. Ronau, J. A. *et al.* An additional substrate binding site in a bacterial phenylalanine hydroxylase. *Eur. Biophys. J.* **42**, 691–708 (2013).
18. Andreas Andersen, O., Flatmark, T. & Hough, E. Crystal structure of the ternary complex of the catalytic domain of human phenylalanine hydroxylase with tetrahydrobiopterin and 3-(2-thienyl)-l-alanine, and its implications for the mechanism of catalysis and substrate activation. *J. Mol. Biol.* **320**, 1095–1108 (2002).
19. Cha, L. *et al.* Mechanistic studies of aziridine formation catalyzed by mononuclear non-heme iron enzymes. *J. Am. Chem. Soc.* **145**, 6240–6246 (2023).

Uncropped gels

a.

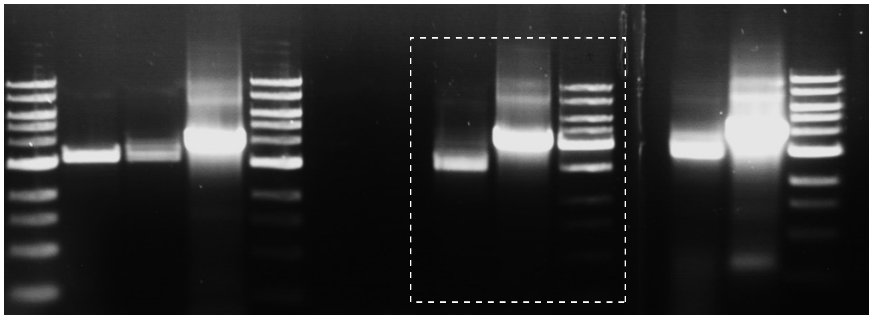

b.

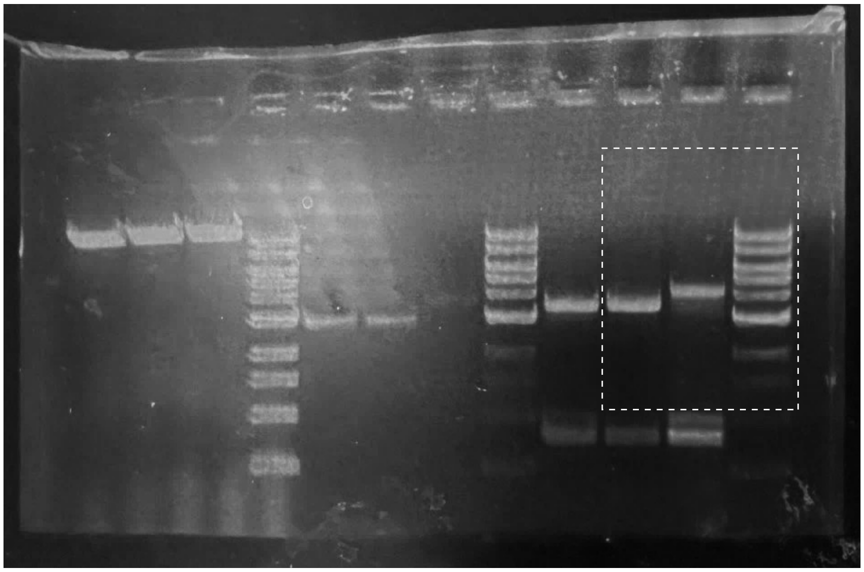

Uncropped scans of DNA gels in Supplementary Figure 1 b.

a.

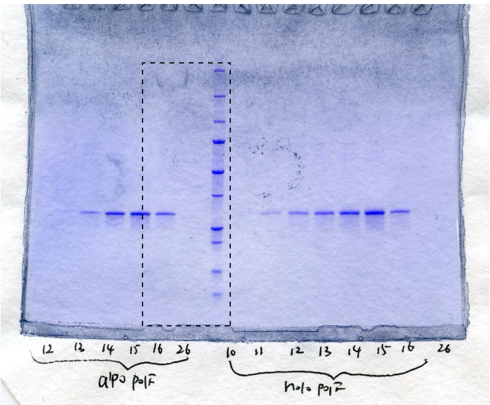

b.

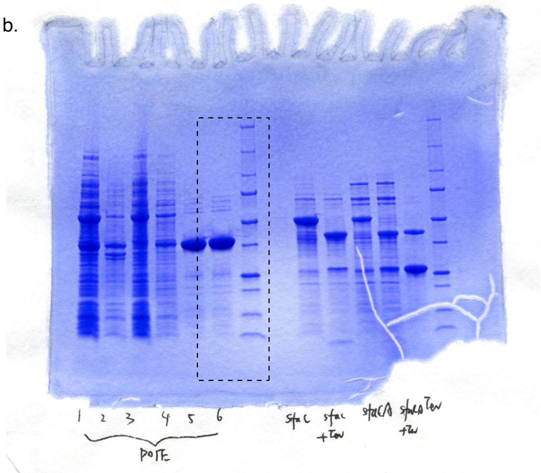

c.

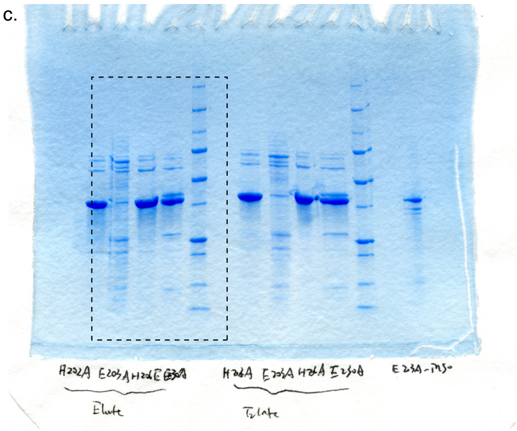

Uncropped scans of SDS gels in Supplementary Figure 3 b.
