# Supplementary figures and images for: Azetidine amino acid biosynthesis by non-haem iron-dependent enzymes
Source: Nat Chem. 2025 Oct 21;18(3):492–501. doi: 10.1038/s41557-025-01958-x (PMC12614342; doi:10.1038/s41557-025-01958-x)

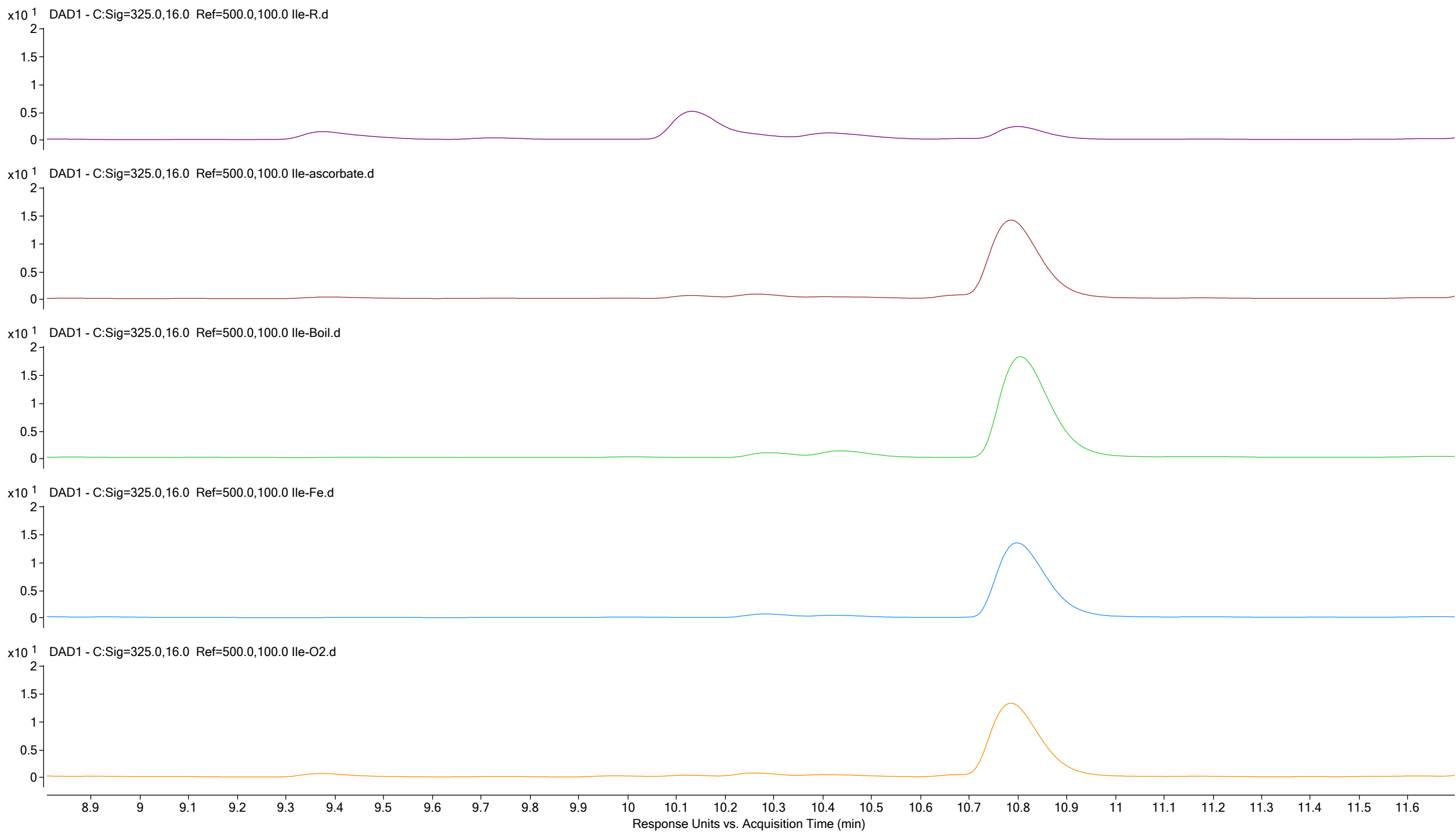

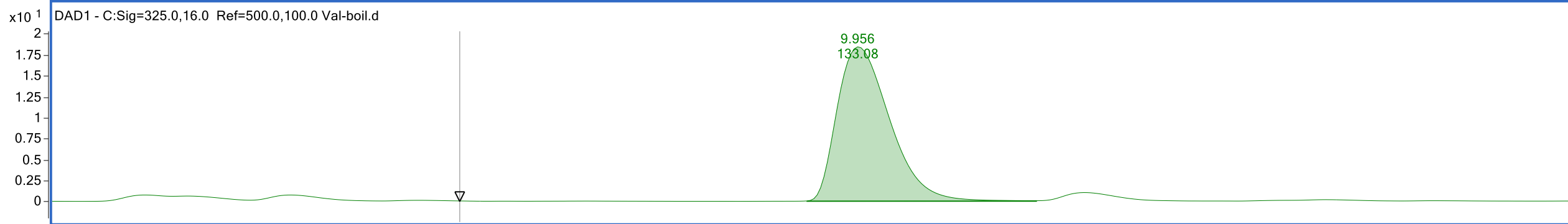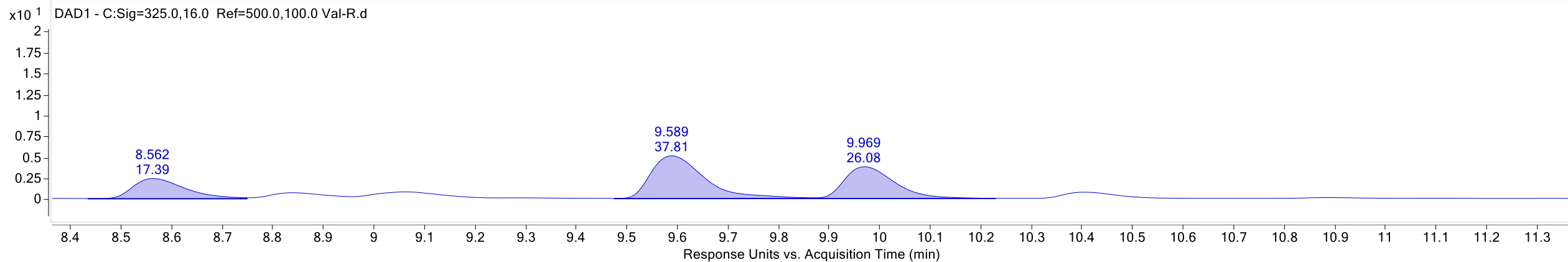

Supplement: Supplementary file 8 — Fig. 1a–c original HPLC figures. [file 41557_2025_1958_MOESM8_ESM.pdf]

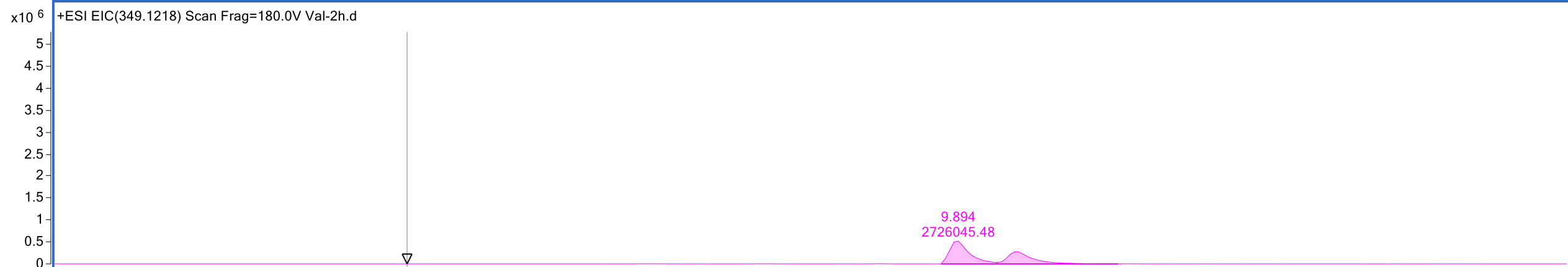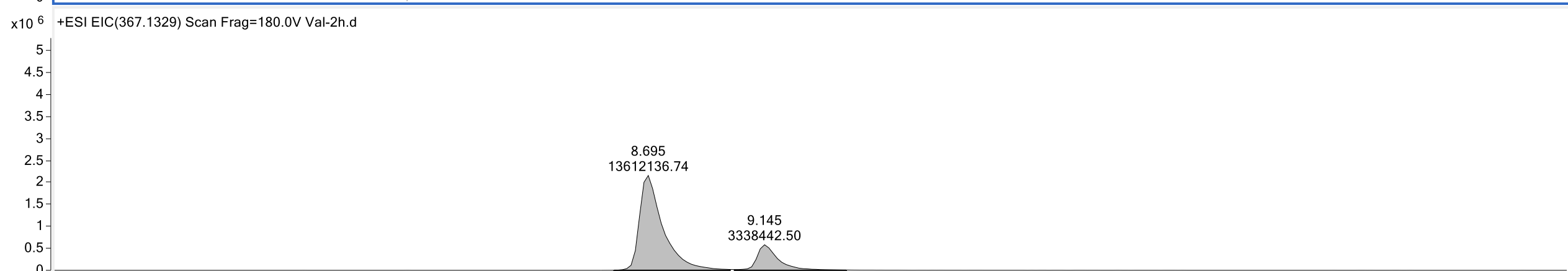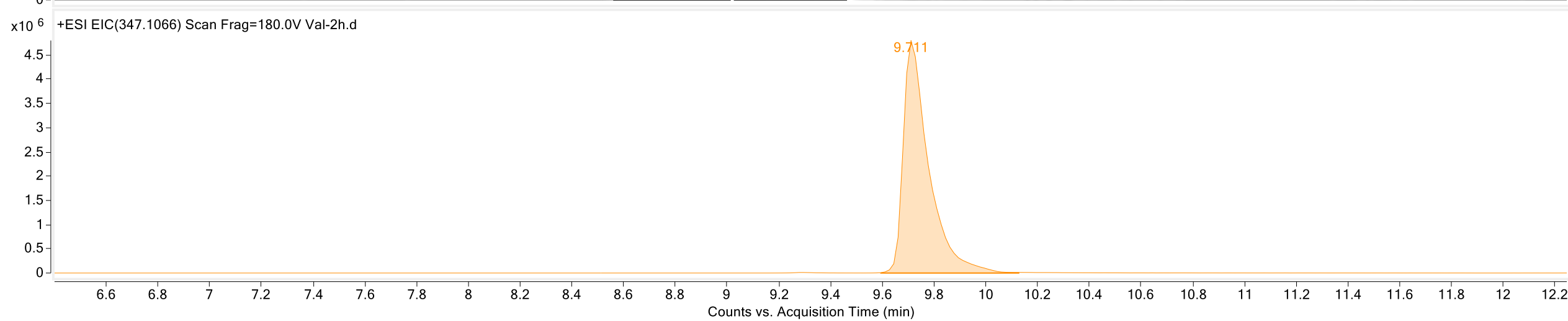

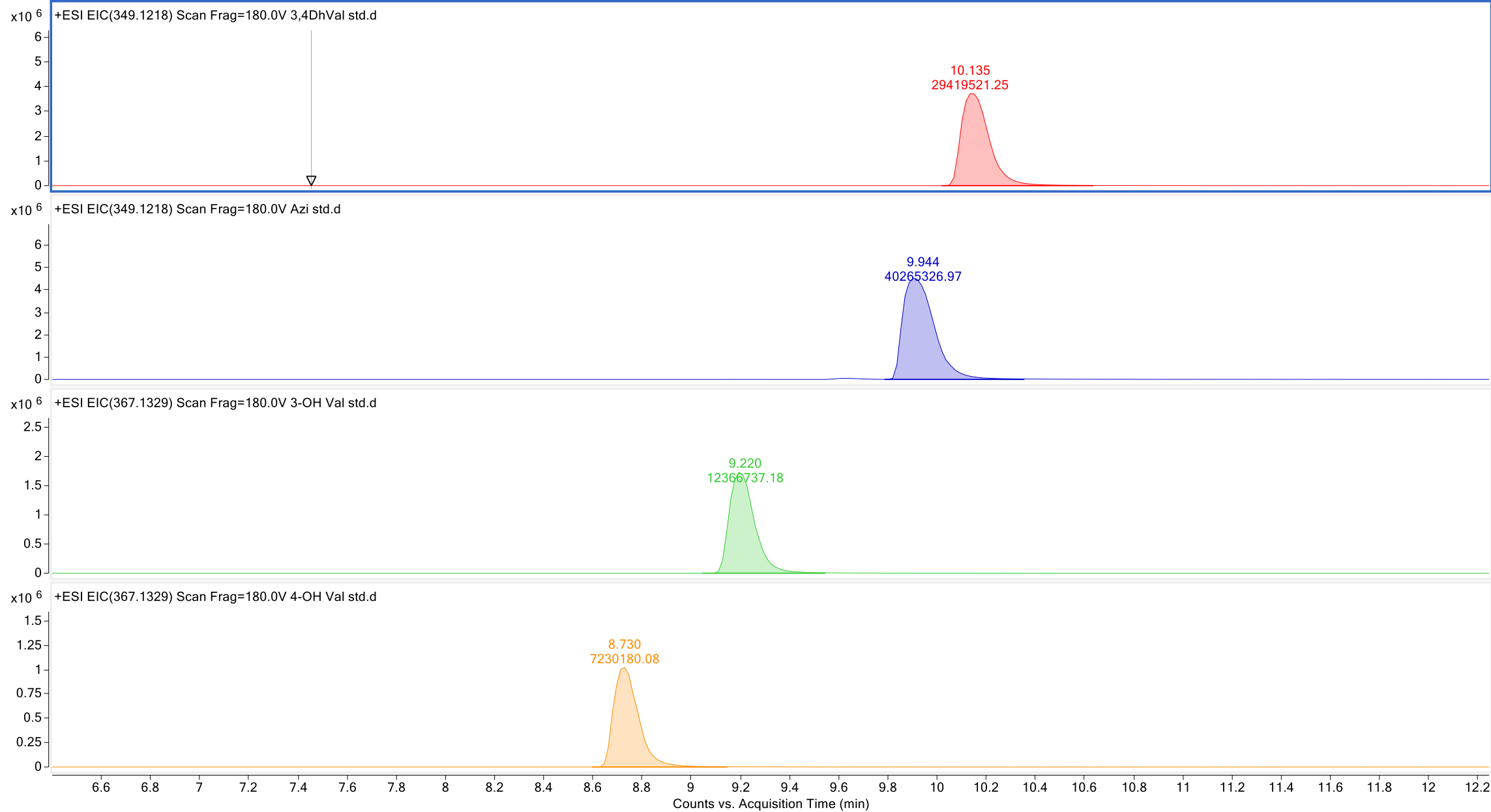

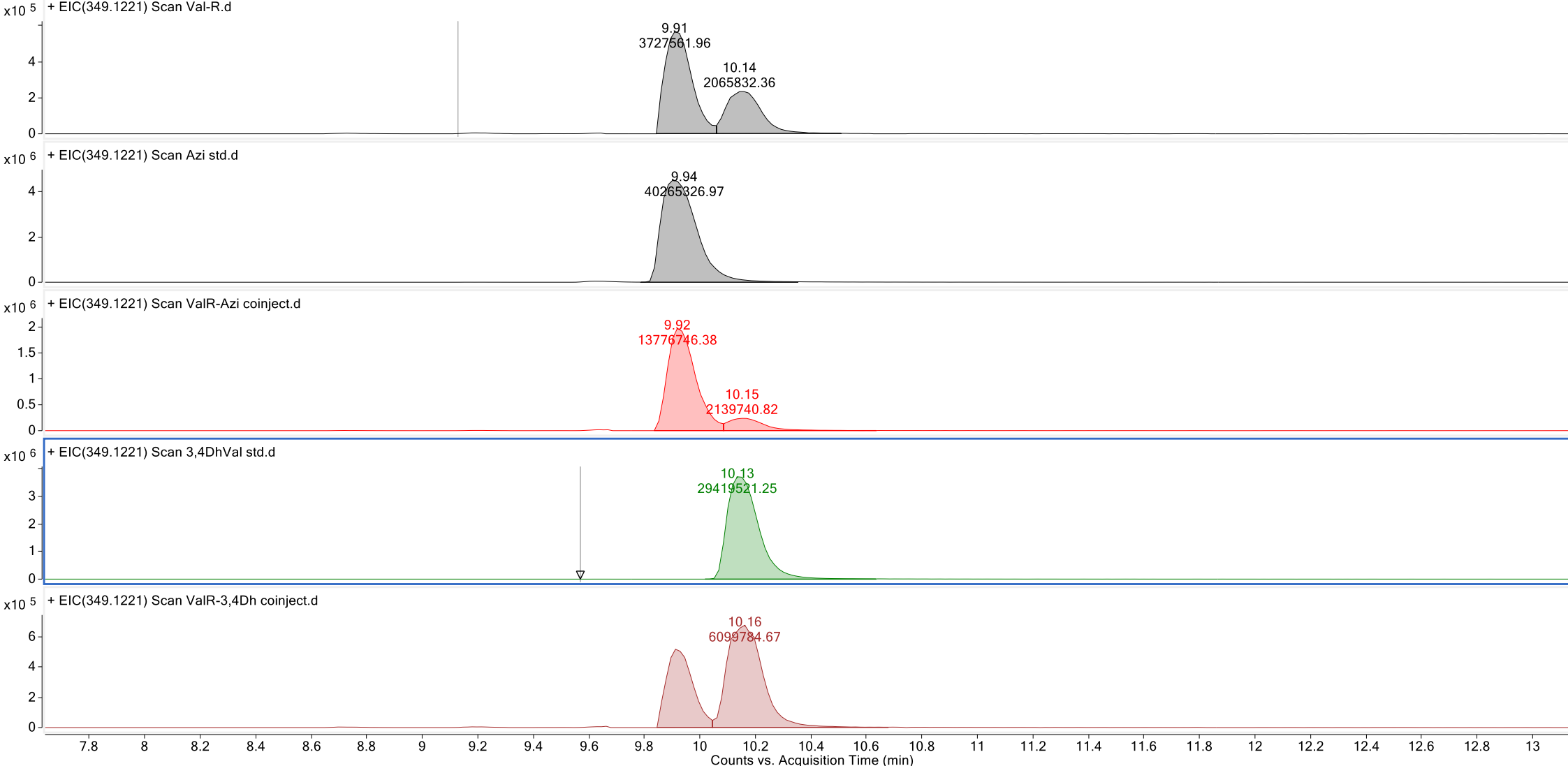

Supplement: Supplementary file 9 — Extended Data Fig. 2a,b original HPLC figures. [file 41557_2025_1958_MOESM9_ESM.pdf]

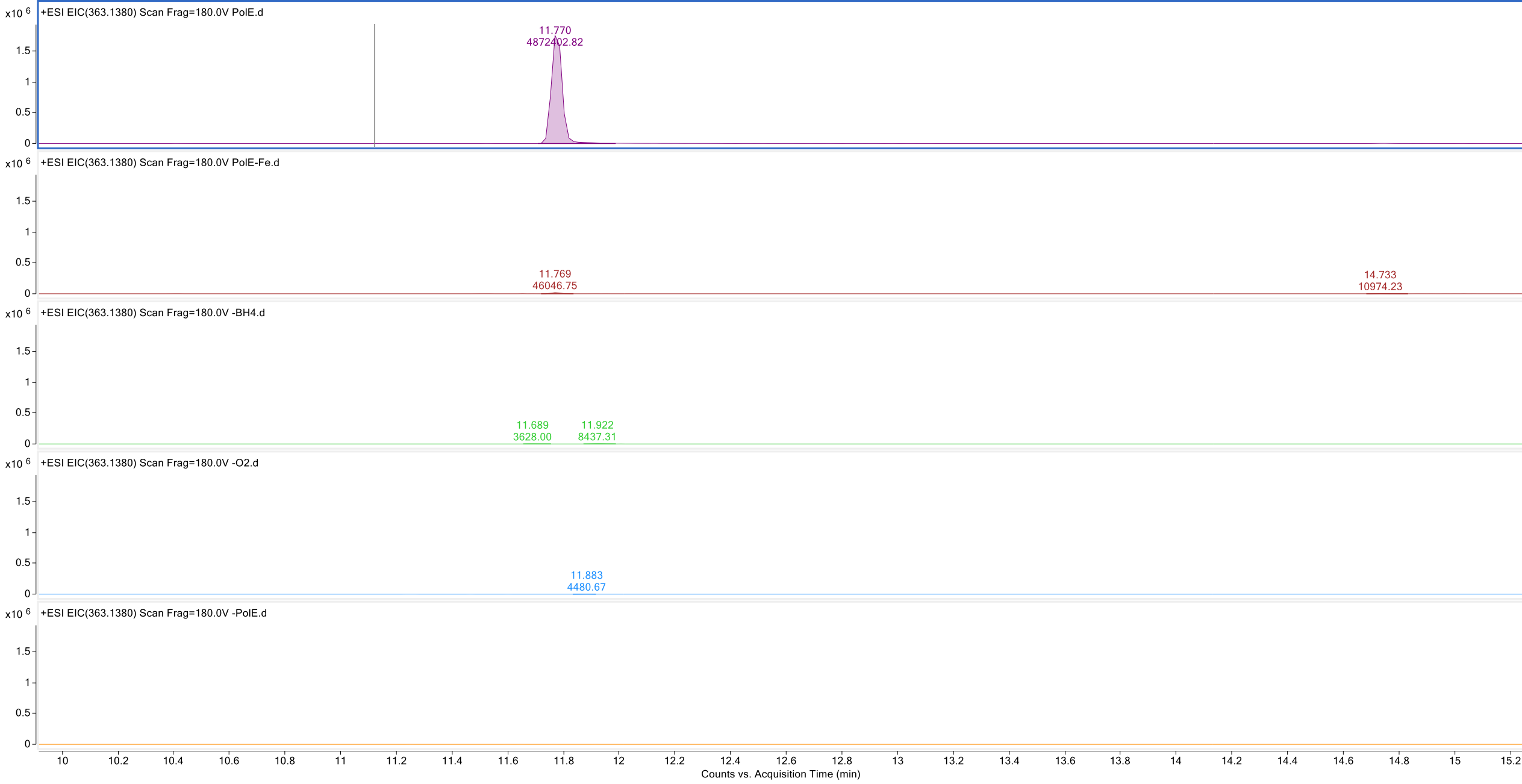

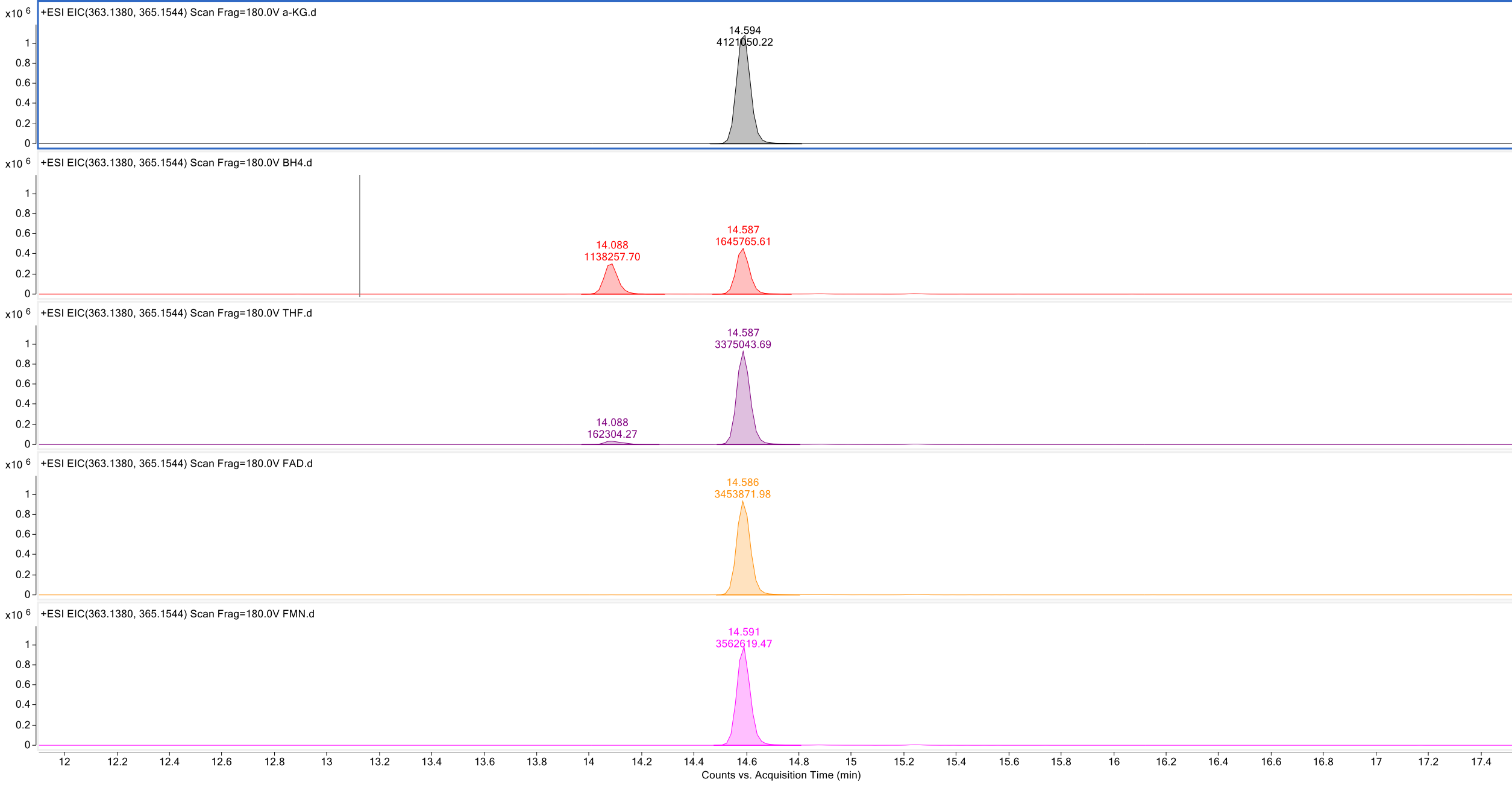

Supplement: Supplementary file 11 — Extended Data Fig. 7a,b original HPLC figures. [file 41557_2025_1958_MOESM11_ESM.pdf]

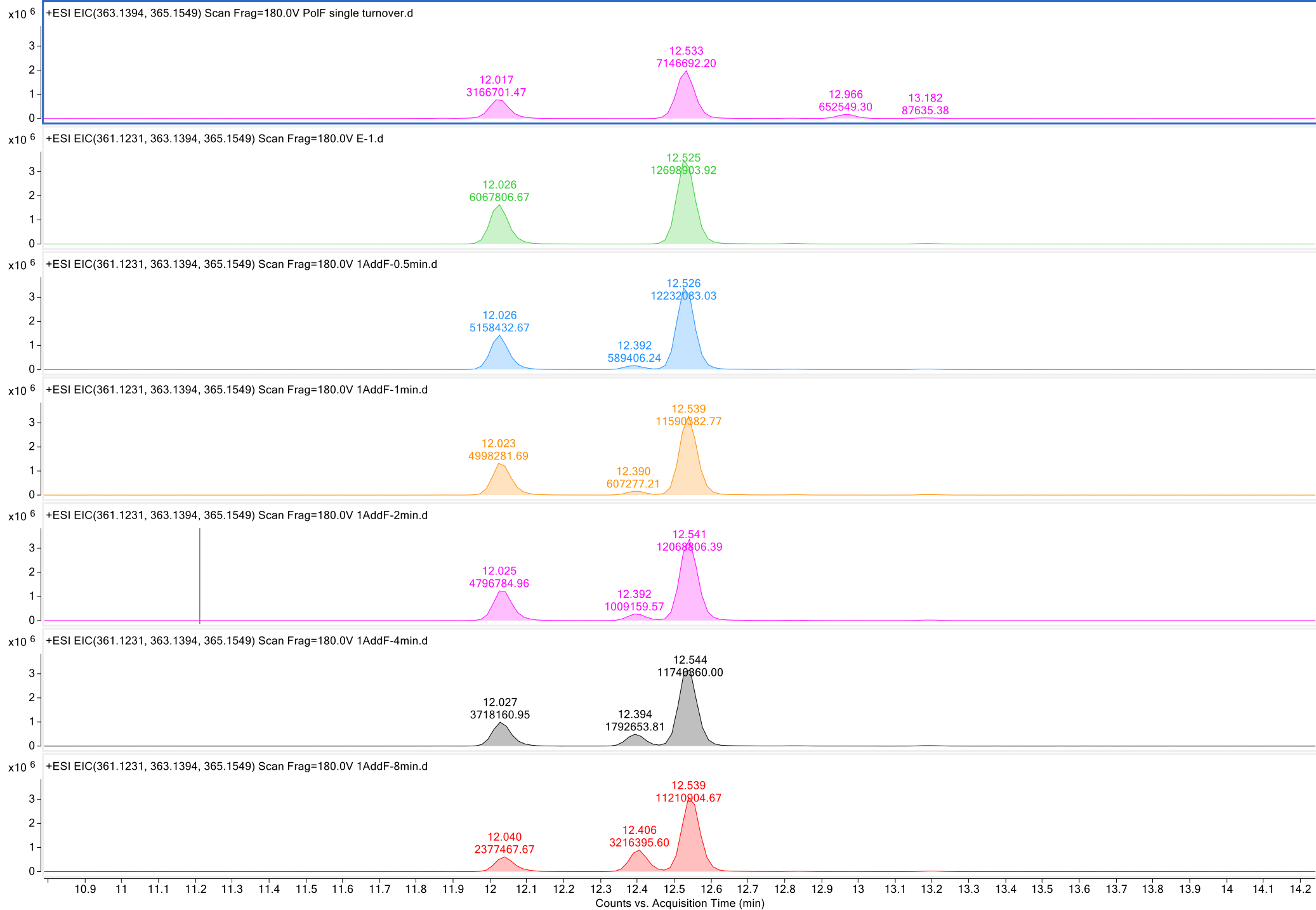

Supplement: Supplementary file 12 — Extended Data Fig. 8 original HPLC figures. [file 41557_2025_1958_MOESM12_ESM.pdf]
